# CDW19S coordinates phasic end processing via distinct enzymatic activities

**DOI:** 10.1093/nar/gkag681

**Published:** 2026-07-06

**Authors:** Ming Zeng, Zizhi Tang, Chunyi Li, Laifeng Ren, Xiaojun Wang, Antony Carr, Cong Liu

**Affiliations:** Department of Experimental Research, Precision Radiation in Oncology Key Laboratory of Sichuan Province, Sichuan Cancer Hospital & Institute, Sichuan Cancer Center, University of Electronic Science and Technology of China, Chengdu 610041, China; Department of Paediatrics, Key Laboratory of Birth Defects and Related Diseases of Women and Children (Ministry of Education), West China Second University Hospital, Sichuan University, Chengdu 610041, China; Department of Paediatrics, Key Laboratory of Birth Defects and Related Diseases of Women and Children (Ministry of Education), West China Second University Hospital, Sichuan University, Chengdu 610041, China; Central Laboratory, Shanxi Hospital Affiliated to Cancer Hospital, Chinese Academy of Medical Sciences, Taiyuan 030000, China; Department of Paediatrics, Key Laboratory of Birth Defects and Related Diseases of Women and Children (Ministry of Education), West China Second University Hospital, Sichuan University, Chengdu 610041, China; Genome Damage and Stability Centre, School of Life Sciences, University of Sussex, Brighton BN1 9RQ, United Kingdom; Department of Paediatrics, Key Laboratory of Birth Defects and Related Diseases of Women and Children (Ministry of Education), West China Second University Hospital, Sichuan University, Chengdu 610041, China; Genome Damage and Stability Centre, School of Life Sciences, University of Sussex, Brighton BN1 9RQ, United Kingdom

## Abstract

In response to DNA double-strand breaks (DSBs), 5′-3′ resection is required for production of single-stranded DNA (ssDNA) and commitent of homologous recombination (HR). Here, we demonstrated that CDW19S, a DSB-bound 19S proteasome variant, coordinates phasic control of long-range resection in a spatial-separated manner. Phase I exploits a panel of ubiquitin modifications on RAP80 (6Kub) as a hesitation mechanism to restrain BRCA1 loading. The deubiquitinase POH1, an integral component of CDW19S, removes 6Kub to allow BRCA1 assembly with BRCA1–A complex and firing of extensive resection. Following the action of phase I apparatus that is metazoan-specific, evolutionarily conserved phase II takes the relay in more distal compartments by imposing CDW19S-engaged CRL4^WDR70^ E3 ligase to degrade ADRM1, leading to the full-range ssDNA production dedicated for HR activation. The phasic regulation stimulates the repositioning of 53BP1-dependent resection barriers: 6Kub removal and BRCA1 loading overcome the 53BP1/PTIP barrier in phase I, and the demolition of ADRM1 antagonizes 53BP1/RIF1. Importantly, the phasic control of extensive resection serves for the tight control of ssDNA production, securing HR activation and preventing toxic repair mechanisms. Aggregately, our work reveals a coordinative function of CDW19S facilitating sufficient end resection that is crucial for error-free DNA repair.

## Graphical Abstract

**Graphical Abstract:**
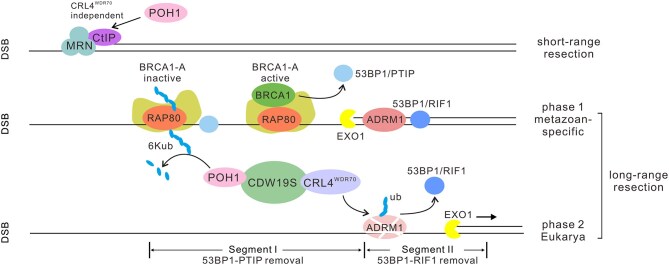

## Introduction

DNA double-strand breaks (DSBs) are highly toxic to cells, and failed or incorrect DSB repair leads to chromosomal instability and carcinogenesis [1, 2]. Two primary mechanisms, non-homologous end joining (NHEJ) and homologous recombination (HR), are key to DSB repair. NHEJ requires minimal DNA end processing but is intrinsically error-prone. HR requires extensive 5′-to-3′ end resection to generate a region of RPA-coated single-stranded DNA (ssDNA). This enables a homology search for the undamaged template DNA that is used for error-free repair [3]. Multiple considerations, such as cell cycle stage and chromatin context, influence the choice between NHEJ and HR, which is mainly controlled by the initiation and progression of resection [4, 5]. Many pathway choice mechanisms impinge on the occupancy of two key proteins, 53BP1 and BRCA1, at the chromatin surrounding the DSB site [6–8]. 53BP1 and its effector proteins RIF1 [9] and PTIP [10] suppress resection and thus promote NHEJ. In contrast, BRCA1 and associated protein factors displace 53BP1 from loci proximal to the DSB ends, thus allowing long-range resection and the commitment of HR [11]. NHEJ and HR are competitive and their proper usage is crucial for the maintenance of genome stability [12, 13].

The DSB loading of BRCA1 requires the formation of the BRCA1–A complex (Abraxas-RAP80-BRCA1-BRCC36-MERIT40/NBA1), which depends on RAP80 reading of ubiquitin signals at DNA breaks [14]. RAP80 binds to K63-linked ubiquitin chains on histone H2A and nearby proteins at DNA damage sites, modifications catalyzed by the sequential actions of the RNF8/RNF168 ubiquitin ligases [15, 16]. While RAP80 is crucial for recruiting BRCA1 to the damage site, it paradoxically sequesters BRCA1 to prevent the premature assembly and activity of other pro-resection BRCA1-containing complexes, such as the BRCA1–CtIP resection machinery, thus limiting end processing [17]. This creates a transient braking mechanism, ensuring end resection is controllable and preventing toxic hyper-recombination [18]. Therefore, the BRCA1–A complex helps fine-tune the initial chromatin environment around DNA breaks, but the mechanism is not entirely understood.

Our previous study identified an HR regulatory complex, coined CDW19S (CULLIN4A-DDB1-WDR70-19S) [19] and consisted of 19S RP scaffold and CULLIN4-DDB1 E3 ubiquitin ligase (CRL4^WDR70^). CDW19S orchestrates both MRE11 and EXO1 nucleases through well-defined functional subdomains: the MRE11-regulatory module on 19S RP stabilizes MRE11 to initiate end processing at proximity of DNA breaks; subsequently, CRL4^WDR70^ engages to the EXO1-specific module to catalyze the ubiquitin-mediated degradation of a resection barrier (ADRM1) epistatic to 53BP1, thus activating EXO1 and extensive resection. Further, we demonstrated that the HBV-encoded oncoprotein HBx dissociates CUL4–DDB1 from WDR70, resulting in viral E3 enzyme (CRL4^HBx^ [20]) and disintegrated CDW19S. This viral sabotage stabilizes ADRM1 and inhibits long-range resection, eventually producing a viral subtype of homologous recombination defect.

As described above, the resection mechanism involves many factors and pathways, appearing larynthic and lack of a control hub coordinating each step. Here, we discover two-phase regulation of long-range resection as defined by 53BP1-associated proteins PTIP and RIF1, respectively, functioning in separate segments from DSB-distal. Phase I regulation is metazoan-specific and relies on the modification and deubiquitination of polyubiquitinated RAP80 (6Kub), which provides the on–off signal for BRCA1 recruitment to DSB and commencement of extensive resection. The 6Kub signals serve as a hesitation mechanism to suppress hyperactive resection and misuse of toxic repair mechanism of single-strand annealing (SSA), thus safeguarding genome integrity. Phase II is evolutionarily conserved from yeast to human and acts at more distal regions of phase I control, which endorse HR. Importantly, CDW19S plays coordinative roles in each phasic regulation aiming to expel 53BP1 resection barricades via integrated enzymatic modules: the intrinsic DUB activity of 19S RP subunit (POH1) directly scraps 6Kub from RAP80 to overcome 53BP1/PTIP in phase I, and CRL4^WDR70^-dependent degradation of ADRM1 triggers 53BP1/RIF1 elimination in phase II. Together, our findings define CDW19S as a coordinator for DNA end processing, purposing the generation of sufficient and high-quality ssDNA products through a series of actions to antagonize 53BP1.

## Materials and methods

### Cell lines and reagents

Human cell lines (HEK293T, *WDR70^KO^*, L02, and T43) were maintained in Dulbecco’s modified Eagle’s medium (DMEM) supplemented with 10% fetal bovine serum (ExCell). T43 cells with stably integrated HBV-genomes were regularly selected in G418 (200 μg/ml) and examined for titration of HBs and HBe antigens by enzyme-linked immunosorbent assay. All cell lines were tested negative for mycoplasma contamination. Plasmid and siRNA transfections were generally carried out using Lipofectamine 3000 (Invitrogen, L3000015). Plasmids and siRNA used in this study are listed in Supplementary Tables S1 and S2, respectively. For ATM kinase, CHK1, and CHK2 inhibition, 5 μM KU-55933 (Selleck, S1092), 2.5 μM LY2603618 (Selleck, S2626), or 2.5 μM BML-277 (Selleck, S8632) was used for pretreatment for 3 h.

### Plasmids

For cloning of lentiviral vectors for expressing Flag-tagged proteins, PCR (polymerase chain reaction ) fragments were inserted into the EcoRI sites of pLVX-G-Flag plasmid, using the In-Fusion cloning kit (Clontech, 639650). *6KR* mutant of *RAP80* was converted from pLVX-G-Flag-RAP80 or pcDNA3.1-RAP80 using QuikChange Lightning Site-Directed Mutagenesis Kits (stratagene, 200519). The *K48K63* ubiquitin mutant was converted from pcDNA3.1-V5-Ubiquitin-K48-only using a 3′-primer containing a mutation of *R63K* and inserted into BamH1/Xba1 sites.

### Western blot

Western blot was performed using a standard protocol. Briefly, samples were resuspended in 1× sodium dodecyl sulfate (SDS) buffer and boiled for 10 min. Proteins were separated by sodium dodecyl sulfate–polyacrylamide gel electrophoresis (SDS–PAGE) and then transferred onto PVDF membranes (Bio-Rad, 1 620177). The membranes were blocked with 1× Blocking Reagent (Roche, 11921673001) for 1 h at room temperature and then incubated overnight at 4°C with primary antibodies diluted in Blocking reagent. After washing three times with TBST, membranes were incubated with appropriate HRP-conjugated secondary antibodies for 1 h at room temperature. Protein bands were visualized using an enhanced chemiluminescence detection system and imaged with the Bio-Rad ChemiDoc imaging system. Antibodies used in this study were listed in Supplementary Table S3.

### Preparation of metaphase chromosomal spread

Cells were plated in a 6-cm dish and arrested in mitosis by 2-h treatment with colcemid (final concentration of 200 ng/ml). Pre-warmed 75 mM KCl was then added to the trypsinized cells and incubated for 15 min at 37°C, followed by adding four drops of freshly prepared fixative (3:1 methanol:acetic acid). Cells were pelleted, resuspended in 5 ml fixative, and incubated for 20 min at 4°C. After repeating the fixing step three or four times, pellets were resuspended in 0.5 ml of fixative solution. Two or three drops of fluid were precipitated onto a pre-chilled slide from a height of about 18 inches. Slides were thoroughly air-dried and stained using the Giemsa protocol. The mitotic chromosomes were observed and evaluated using an Olympus fluorescence microscope (BX51) at 1000× magnification.

### Pull-down for ubiquitinated RAP80 and identification of ubiquityl-lysine residuals by LC-MS

To detect *in vivo* polyubiquitinated RAP80, cells co-transfected with pcDNA3.1-ubiquitin (in-frame fused with either HA or V5 tag) and pLVX-Flag-RAP80 were harvested 72 h later. Cell pellets were lysed in IP2 buffer (10 mM Tris–HCl, pH 7.4, 100 mM NaCl, 1% NP40), followed by immunoprecipitation of RAP80 with anti-Flag M2 beads (Sigma, M8823). Enriched RAP80 was released by boiling in SDS sample buffer and resolved by 10% SDS–PAGE for detecting the fraction of ubiquitin-conjugated-RAP80 by rat anti-HA or mouse anti-V5, and total RAP80 by mouse anti-Flag, respectively.

To identify modification sites on ubiquitinated RAP80, pellets from eight 10-cm dishes of *WDR70* knockout 293T cells were subjected to anti-Flag pulldown as described above. Protein bands of 75 kDa (IP1) and >90 kDa (IP2) visualized by Coomassie-blue staining (Sigma, B2025) were excised and subjected to liquid chromatography-mass spectrometry (LC-MS) analysis by PTM Biolabs, Hangzhou. In brief, gel pieces were in-gel digested with 10 ng/μl trypsin at 37°C overnight. Peptides were sequentially extracted with 50% acetonitrile/5% formic acid and 100% acetonitrile. The tryptic peptides were separated via a home-made reversed-phase analytical column (15 cm length, 75 μm i.d.) and subjected to NSI source followed by tandem mass spectrometry (MS/MS) in Q Exactive™ Plus (Thermo) coupled online to the Ultra Performance Liquid Chromatography (UPLC). The electrospray voltage applied was 2.0 kV. The *m*/*z* scan range was 350–1800 for full scan, and intact peptides were detected in the Orbitrap at a resolution of 70 000. Peptides were then selected for MS/MS using NCE setting as 28 and the fragments were detected in the Orbitrap at a resolution of 17 500. The resulting MS/MS data were processed using Thermo Proteome Discoverer (Version 1.3.0.339).

### Colony formation assay

For the colony formation assay, cells were seeded at 500 cells per 6-cm dish and cultured at 37°C for ~10–14 days with indicated treatment. Subsequently, cells were fixed with 4% paraformaldehyde (PFA) and stained using Crystal Violet Staining Solution (Beyotime, C0121). Images were captured utilizing the Tanon MINI Space system.

### *In vitro* deubiquitination assay

For the *in vitro* deubiquitination assay, 1 × 10^7^ 70KO cells transfected with pLVX-Flag-RAP80 were harvested 48 h post-transfection. Cells were lysed, and Flag-tagged RAP80 was immunoprecipitated using anti-Flag M2 beads. The bead-bound Flag-RAP80 complexes were resuspended in 20 μl of phosphate-buffered saline (PBS), followed by the addition of 1 μg of recombinant GST-tagged POH1 (Sino Biological, Cat# P501-30G). After incubation at 37°C for the indicated time points, the reactions were terminated by adding 5× SDS sample buffer and then subjected to detection by western blot.

### Synchronization-release experiment

Cell cycle synchronization at the G1/S boundary was achieved through a double thymidine block protocol. Briefly, cultured cells were incubated for 16 h with DMEM containing 2 mM thymidine (Selleck, S4803). After the first block, cells were thoroughly washed twice with pre-warmed PBS to remove thymidine and then cultured in fresh medium for 9 h to allow cell cycle progression. Following this release period, cells were exposed again to DMEM with 2 mM thymidine for an additional 16 h. To generate synchronous populations in the S and G2 phases, cells were re-fed with fresh DMEM and harvested at 4 and 8 h, respectively.

### Immunofluorescence

Indirect immunofluorescence was performed as previously described [21]. Briefly, cells grown on coverslips were treated with 2 Gy IR (Precision, X-Rad 320), followed by recovery at indicated time points, then fixed in 4% PFA. Permeabilized sections were incubated with the indicated primary antibodies listed in Supplementary Table S3, followed by appropriate secondary antibodies conjugated to Cy3 (anti-rabbit) or FITC (anti-mouse). Samples were mounted using an anti-fade medium containing DAPI and visualized by an Olympus BX51 fluorescence microscope. For quantitative analysis, ~100 cells per condition were counted from three independent experiments.

For non-denatured BrdU staining, cells on coverslips were pre-labeled with 40 μg/ml BrdU for 2 days and fixed with methanol–acetic acid buffer (3:1) after irradiation. The following procedures were performed as described above.

### Measurement for efficacies of DSB repair

For plasmid-based DSB repair assay of HR, SSA, and NHEJ [22], pCMV plasmids containing an I-SceI restriction site were digested *in vitro*. Digested plasmids (5 µg for NHEJ and SSA, 10 µg for HR) were transfected into the indicated cells and allowed to undergo *in vivo* repair for 48 h. Plasmids recombined via SSA or HR or ligated via NHEJ were subsequently recovered using a HighPure PCR Template Preparation Kit (Roche, 11796828001). The recovered DNA was quantified by quantitative reverse transcription PCR (RT-qPCR) on a Bio–Rad CFX96 system with primers specific to SSA/HR or NHEJ repair products (see Supplementary Table S4). The amount of extracted plasmid DNA was normalized using primers 1/2. The relative frequency of each repair pathway was calculated as a fold change compared to qPCR values obtained from repaired DNA extracted in parallel from wild-type or mock-infected 293T cells.

For alt-EJ analysis, 293T and L02 cells transfected with the EJ2-GFP-PURO plasmid were maintained in medium containing 1 µg/ml puromycin. Following the indicated treatments, cells were transfected with the pCBASce plasmid. The percentage of GFP-positive cells was measured 48 h later, and the relative repair frequency was normalized to that of wild-type cells.

### Chromatin immunoprecipitation

CRISPR-induced DSB and ChIP protocols were described previously with minor modifications [21]. Briefly, CRISPR-gRNA vector (pMC1-1-p84-g1, targeting sequence: 5′-GTCCCCTCCACCCCACAGTGGGG-3′) was transfected 24 h before genomic DNA extraction. An empty CRISPR-gRNA plasmid expressing no targeting gRNA was applied as the negative control. For location analysis by ChIP, ~4 × 10^6^ cells were cross-linked with 1% formaldehyde, lysed with Lysis Buffer [50 mM HEPES (pH 7.4), 140 mM NaCl, 1% Triton X-100, 0.1% NaDeoxycholate, 1 mM ethylenediaminetetraacetic acid (EDTA) supplemented with protease inhibitor cocktail (Roche, 11 836 170 001)] and sonicated at 4°C. Protein G Dynabeads (Invitrogen, 10004D) pre-coated with 2 μg indicated antibodies or anti-Flag M2 beads (Sigma, M8823) were applied to retrieve chromatin-associated proteins. Beads were sequentially washed with lysis buffer, lysis high salt buffer [50 mM HEPES (pH 7.4), 500 mM NaCl, 1% Triton X-100, 0.1% NaDeoxycholate, 1 mM EDTA], wash buffer [10 mM Tris (pH 8.0), 250 mM LiCl, 0.5% NP40, 0.5% NaDeoxycholate, 1 mM EDTA], and TE buffer (pH 8.0). Bound DNA–immune complexes were eluted off the beads in elution buffer [50 mM Tris (pH 8.0), 1% SDS, 10 mM EDTA], and DNA was purified using a Gel Extraction Kit (Omega, D2500-02). Whole cell extracts were treated in parallel. Platinum^®^ Taq DNA Polymerase (Invitrogen, 10966) was used to amplify recovered DNA. All reactions were performed in triplicate.

### Statistics

All histograms were presented as means ± standard deviation. For quantitative analysis, including ChIP assay, image analysis, and repair analysis, at least three independent experiments were carried out. *P*-values were calculated by a two-tailed Student’s *t*-test between two groups, or by a two-way ANOVA test for multiple-group comparison, using GraphPad Prism 9.

## Results

### 53BP1-associated proteins define spatially segregated resection

**Figure 1.**
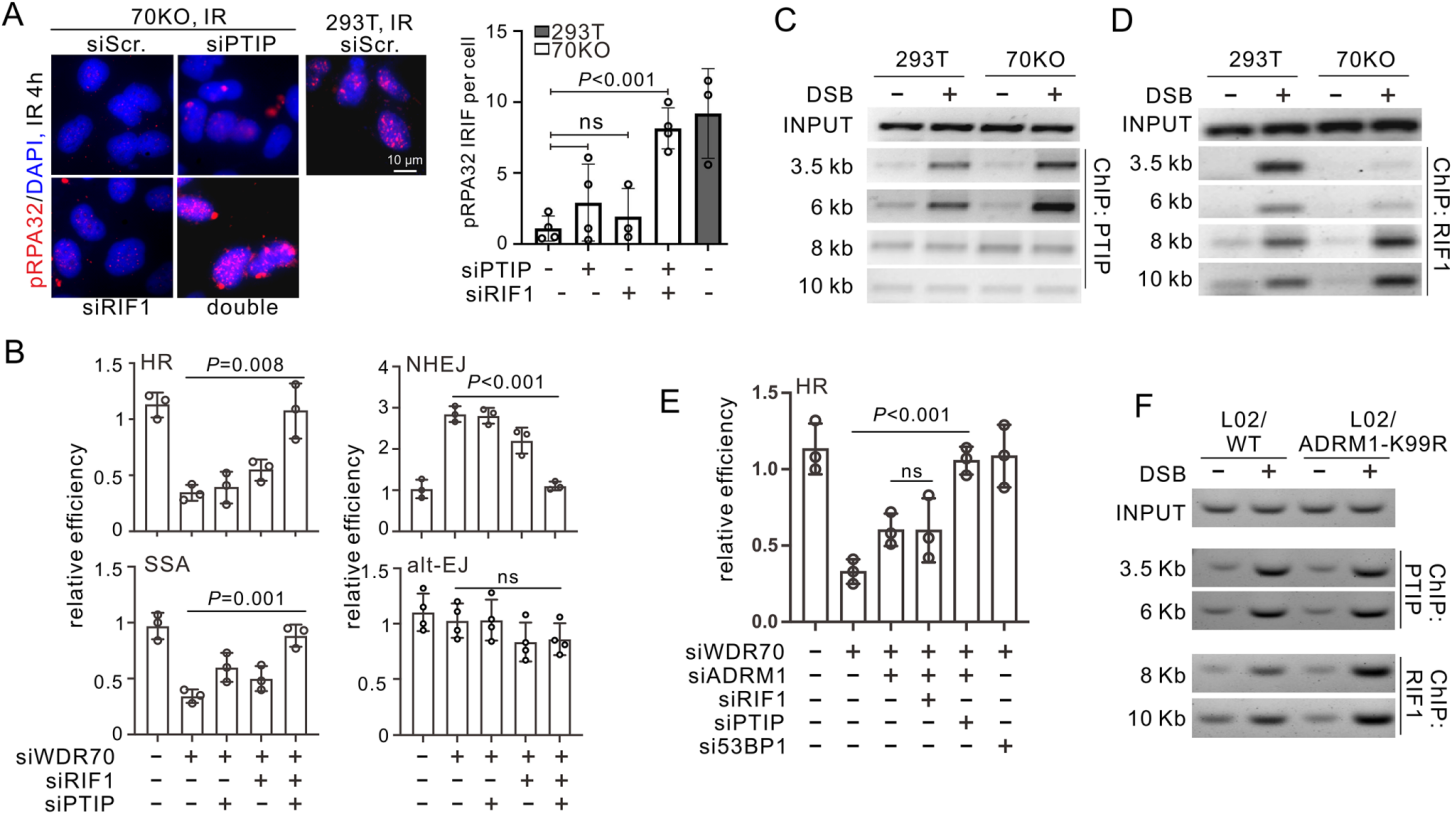
Spatial distribution of 53BP1 effectors defines two-phase regulation for long-range resection. (**A**) Representative images and quantifications of pRPA32 IRIF in *70KO* cells with individual or combinatorial treatment with si*RIF1* and si*PTIP*. Cells were exposed to 2 Gy of irradiation and harvested at the indicated time point. Unless otherwise specified, doses of 2 Gy were used in the following IRIF assays. (**B**) Measurement of HR, SSA, alt-EJ, and NHEJ efficacies using the I-*Sce*I-DSB system in L02 cells treated with si*WDR70*, si*RIF1*, si*PTIP*, or combinations. ChIP analysis for endogenous PTIP (**C**) and RIF1 (**D**) deposited at indicated distances from DSB in 293T and *70KO* cells. (**E**) Measurement of HR in *70KO* cells with indicated treatment. (**F**) ChIP analysis for PTIP and RIF1 in L02 cells upon ADRM1 *WT* or *K99R* mutant expression. All histograms are presented as mean ± s.d. *P*-values were calculated by a two-way ANOVA test.

In CDW19S-deficient cells, depletion of *53BP1* fully restored long-range resection and HR efficiency [19]. 53BP1 inhibits resection and promotes NHEJ by recruiting two effector proteins, RIF1 and PTIP, to the DSB-flanking region via ATM-dependent phosphorylation [23–25] (Supplementary Fig. S1A). We investigated how 53BP1 effectors impact CDW19S-dependent resection, using WDR*70* knockout 293T cells (*70KO*) and an HBV-positive hepatocyte line (T43 [19]). IRIF (irradiation-induced foci) assay demonstrated that co-depletion of both *PTIP* and *RIF1*, rather than ablation of either, achieved complete rescue of resection defects in *70KO* and T43 cells, as evaluated by pRPA32 foci formation, a surrogate signal for ssDNA production (Fig. 1A and Supplementary Fig. S1B). Consistently, we detected a marked restoration of RAD51 recruitment only when both *PTIP* and *RIF1* were concomitantly knocked down in *70KO* cells, dramatically exceeding the impact of individually silenced cells (Supplementary Fig. S1C). Correspondingly, the ISce1-induced DSB reporter system [22] revealed thorough rescue of repair efficiencies of HR and SSA but suppression of NHEJ (Fig. 1B). Of note, as an alternative pathway to compromised homology-dependent repair, the frequency of alt-EJ remained unchanged in these genetic contexts. These data suggest that the 53BP1-dependent resection barrier in compromised CDW19S cells (either by *WDR70* loss or *HBx* expression) is coordinatively established by PTIP and RIF1.

ChIP analysis using a CRISPR-induced site-specific DSB system (Supplementary Fig. S1D) revealed different effects of *WDR70* loss on DSB association for PTIP and RIF1: in control cells PTIP enriched across the 3.5–6 kb regions and showed an increase upon *WDR70* deletion (Fig. 1C). In contrast, RIF1 association with damaged chromatin spanned 3.5–10 kb in WT cells and displayed an increase at 8–10 kb but simultaneous reduction between 3.5–6 kb in *70KO* cells (Fig. 1D). Interestingly, DSB deposition of PTIP and RIF1 appeared mutually exclusive: where the former accumulated (3.5–6 kb in *70KO* cells), the latter decreased; but when *PTIP* was silenced in *70KO*, RIF1 invaded the regions should have belonged to PTIP (Supplementary Fig. S1E and F). This consists to the report that the 53BP1^S25A^ mutation (defective in PTIP binding) enhanced RIF1 association at DSBs [26]. We conclude that PTIP and RIF1 play distinctive roles together with 53BP1 in spatially separated DSB regions, with PTIP being the primary factor that competes with RIF1 at 3.5–6 kb region.

We previously described that CDW19S catalyzes the ubiquitination and degradation of ADRM1 at K99, leading to the removal of 53BP1 [19]. Although ablating *ADRM1* mitigated EXO1 and long-range resection defects caused by *WDR70* depletion, it fails to produce sufficient DSB-loading of BRCA1 and HR frequency [19], implying an ADRM1-independent mechanism contributing to these processes. Interestingly, ADRM1 may act in parallel with PTIP because co-depletion of these proteins in *WDR70*-defective cells reactivated HR comparable to that of si*53BP1*, whereas *RIF1* and *ADRM1* appeared epistatic (Fig. 1E), indicative of ADRM1’s regulation on RIF1. Indeed, ChIP assay using the degradation-insensitive and gain-of-function *ADRM1* mutant (*K99R*, [19]) in L02 cells enhanced RIF1 accumulation at its indigenous regions (8–10 kb), unlike PTIP DSB-dwelling that was not responsive (Fig. 1F). We therefore conclude that CRL4^WDR70^-mediated ADRM1 degradation overcomes 53BP1/RIF1 deposition at distal regions of DSBs. However, the mechanism by which 53BP1/PTIP is dismantled in proximal regions, or if it is caused by *ad hoc* redistribution of factors, remains unknown.

### The dual role of RAP80 implicates an unknown switch for BRCA1

**Figure 2.**
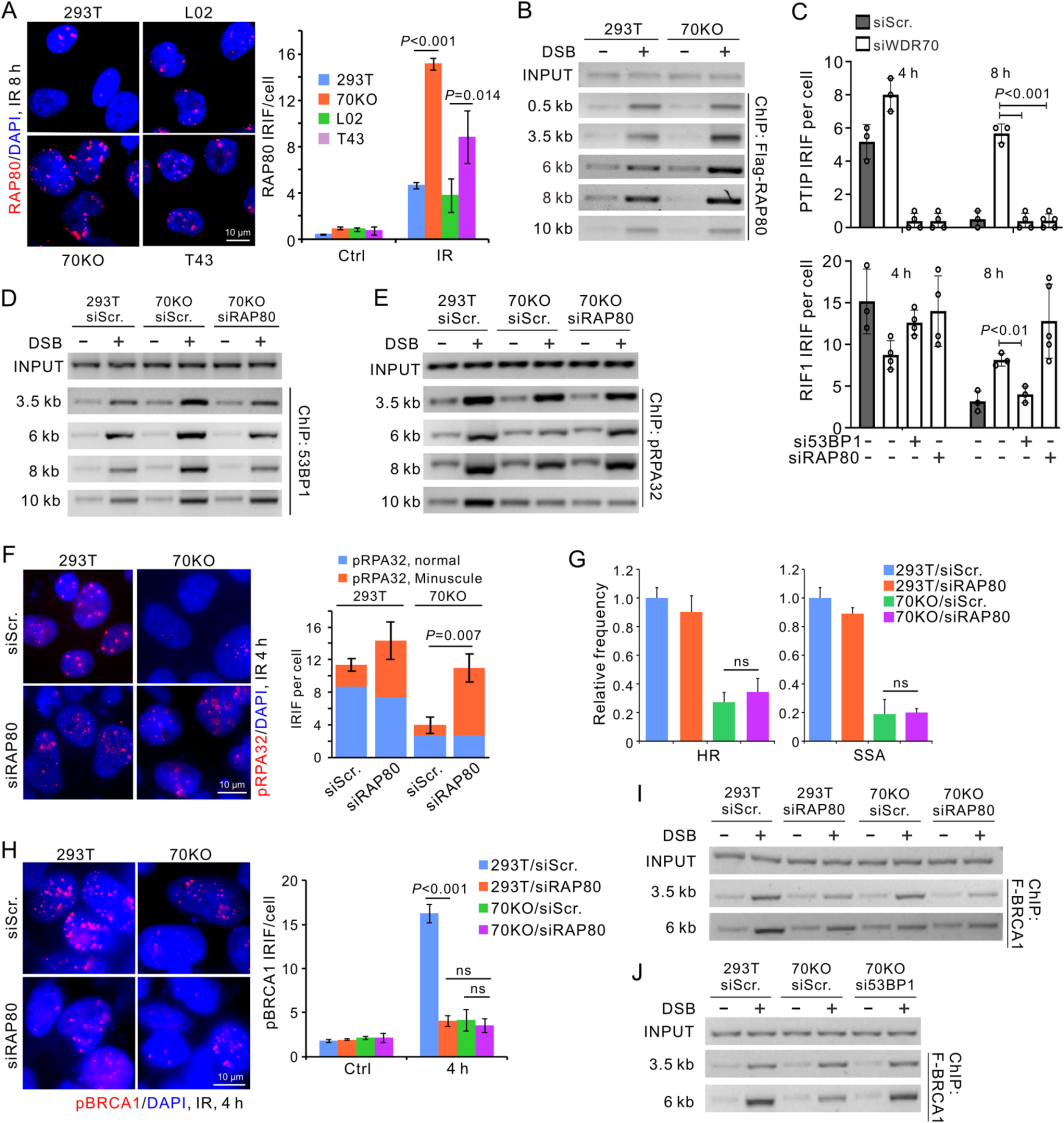
Dual roles of RAP80 regulate 53BP1 repositioning and BRCA1 loading in phase I resection. (**A**) Representative images and quantifications of RAP80 IRIF in indicated cells. (**B**) ChIP assays for DSB loading of Flag-tagged RAP80 in 293T or *70KO* cells. (**C**) Counting for IRIF of PTIP and RIF1 in si*WDR70* L02 cells with *53BP1* or *RAP80* depletion. ChIP assays for 53BP1 (**C**) and pRPA32 (**D**) in indicated cells. (**F**) IRIF morphology and quantifications of pRPA32 in *RAP80*-depleted 293T or *70KO* cells. Note the minuscule RPA32 foci so far as *RAP80* is absent. (**G**) Measurement of HR and SSA efficacies using the I-*Sce*I-DSB system upon *RAP80* silencing. (**H**) IRIF of pBRCA1 in 293T and *70KO* cells with or without *RAP80* depletion. (**I, J**) ChIP detecting DSB-bound Flag-tagged BRCA1 upon indicated treatments. All histograms are presented as mean ± s.d. *P*-values were calculated by a two-way ANOVA test.

To further investigate the ADRM1-independent mechanism by which CDW19S removes 53BP1/PTIP, we investigated the impact of RAP80, the major BRCA1 regulator during early DSB signaling, in the context of *CDW19S* defect. In *70KO* and T43 cells, RAP80 displayed persistent IRIF 8 h after treatment, relative to the respective wild-type cells (293T and L02, Fig. 2A). This mirrored the persistent 53BP1 foci in *70KO* and T43 cells [19], which was effectively reversed by si*RAP80* (Supplementary Fig. S2A and B). Chromatin-bound RAP80 assayed with ChIP was enhanced in *70KO* cells at 3.5–6 kb distal to DSBs, correlating with PTIP-accumulated regions (Fig. 2B). Indeed, si*RAP80* diminished the IRIF of PTIP but not RIF1 in *WDR70*-defective cells, contrasting to the *53BP1* ablation that effectively reverted both (Fig. 2C), indicative of epistasis between RAP80 and PTIP. In ChIP analysis si*RAP80* reverted 53BP1 accumulation and stimulated the re-loading of pRPA32 in *70KO* cells within a similar restricted range, without extending into the distal regions beyond 10 kb (Fig. 2D and E). The limited and zone-specific rescue of si*RAP80* correlated to the restoration of pRPA32 IRIF numbers, but not their size (referred to miniscule foci, Fig. 2F). These phenotypes were reproduced in *WDR70*-defective T43 cells (Supplementary Fig. S2C–E). We conclude that RAP80 assists the establishment of 53BP1/PTIP-mediated roadblock without affecting 53BP1/RIF1. Together with the data in Fig. 1, we posit that PTIP and RIF1 jointly prevent CDW19S-mediated long-range resection in a bipartite manner: PTIP/RAP80 intervenes earlier at 3.5–6 kb proximal to DSBs (referring to phase I), whereas RIF1 cooperates with ADRM1 at more distal regions (8–10 kb, phase II).

Interestingly, partial reversal of 53BP1 accumulation and resection defect by si*RAP80* in *70KO* cells (cf. Fig. 2D and E) did not revitalize HR or SSA efficiency (Fig. 2G). This is attributed to the paradoxical role of RAP80 in loading BRCA1 to DSBs [14, 17], as evidenced by the failure of restoring BRCA1 IRIF in si*RAP80 70KO* double mutant (Fig. 2H). Further, BRCA1 DSB-loading assayed by ChIP was not rigorous in both wild type and *70KO* cells insofar as *RAP80* was depleted (Fig. 2I), contrasting to the impact of si*53BP1* that completely restored BRCA1 recruitment (Fig. 2J). Thus, the dual role of RAP80 resection ensures the timely loading of BRCA1, through establishing the phase I resection barrier of 53BP1/PTIP and thus holding of BRCA1 and an unknown switch to stimulate BRCA1-mediated HR signalling.

### RAP80-6Kub establishes 53BP1/PTIP barriers in phase I

**Figure 3.**
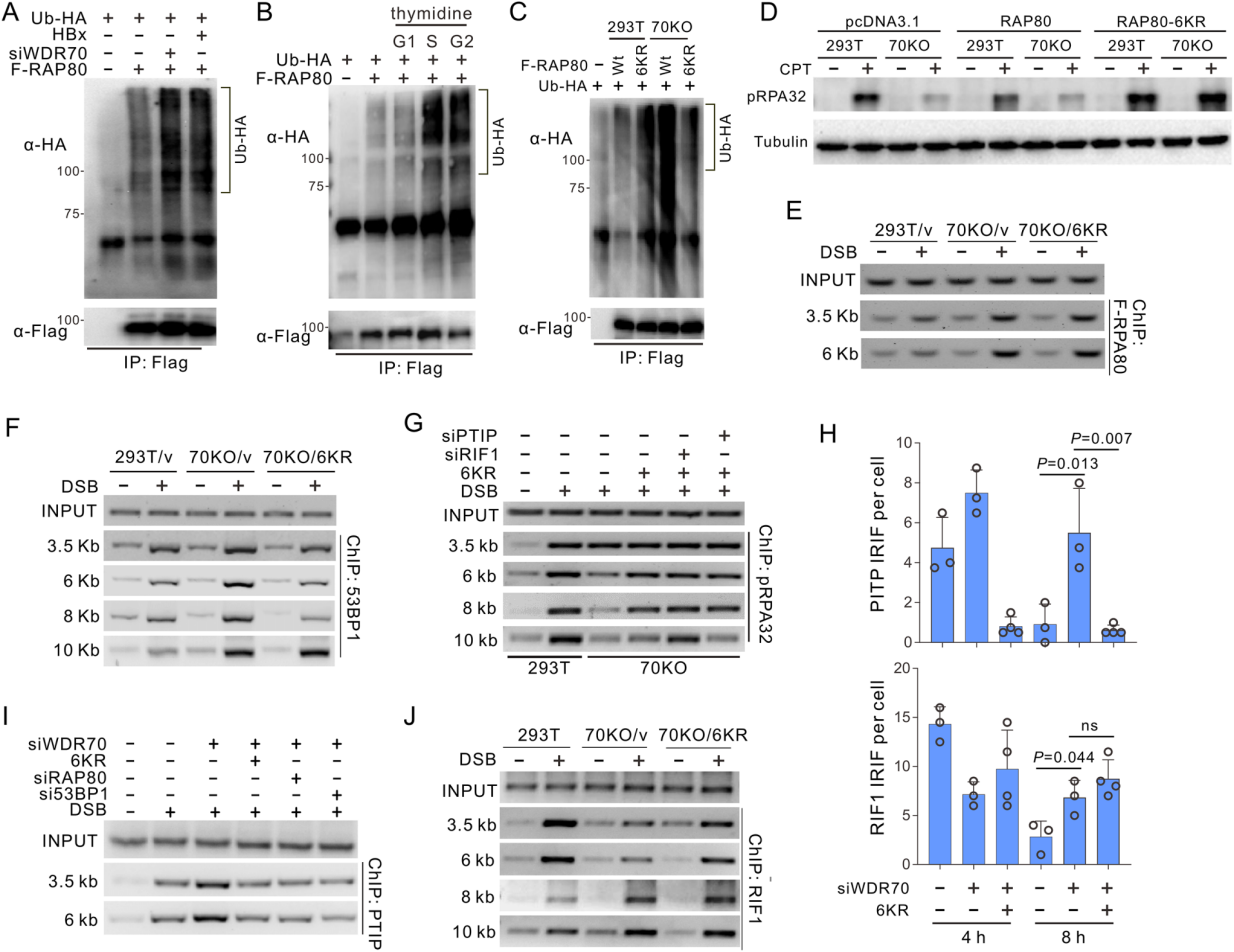
RAP80-6Kub stabilizes 53BP1/PTIP in phase I. (**A**) Flag-tagged RAP80 and HA-tagged ubiquitin were co-transfected into L02 cells. Ubiquitinated RAP80 was pulled down by M2 anti-Flag beads and probed by anti-HA. As in panel (A), ubiquitinated RAP80 was monitored in synchronized 293T cells by block-release of double thymidine treatment (**B**), or in *6KR*-expressing 293T and *70KO* cells (**C**). (**D**) Immunoblotting of pRPA32 in indicated cells upon treatment with 2 μM CPT for 2 h, or not. ChIP assays for RAP80 (**E**) and 53BP1 (**F**) upon introducing *6KR* plasmid or empty pcDNA3.1. (**G**) ChIP assay for pRPA32 in *70KO*/293T cells with individual or combinatorial treatment with si*RIF1*, si*PTIP*, and *6KR*^EE^. (**H**) IRIF counting for PTIP and RIF1 at 4 and 8 h post IR in si*WDR70* L02 cells upon *6KR* expression or not. ChIP analysis showing the effects of *6KR* on DSB-associated PTIP (**I**) and RIF1 (**J**) in indicated cells. All histograms are presented as mean ± s.d. *P*-values were calculated by a two-way ANOVA test.

It was shown that dynamic RAP80 ubiquitination contributes to the pathway choice of DSB repair [27]. To resolve the paradox of the dual role of RAP80, we explored the RAP80 ubiquitin dynamics by co-expressing Flag-RAP80 with HA or V5-ubiquitin in L02 cells, in the context of CDW19S-mediated resection. A reproducible elevation of slow-migrating RAP80 species representing ubiquitin-conjugated RAP80 was observed in *WDR70* knockdown or *HBx* expression cells (Fig. 3A). Notably, the excess of RAP80-ub in *WDR70*-deficient cells is not simply a result of defective HR, as silencing of either *BRCA1* or *BRCA2* did not reproduce the phenotype (Supplementary Fig. S3A). RAP80-ub was S/G2-specific as revealed by double thymidine synchronization-release experiment (Fig. 3B). Further, inhibition of ATM and MDC1, but not CHK1/2, resulted in increased RAP80 ubiquitination in G2-synchronized cells (Supplementary Fig. S3B), indicating that RAP80 ubiquitination is part of DDR network but does not rely on checkpoint activation. Expressing either *K48R* or *K63R* ubiquitin mutant significantly reduced the level of RAP80-ub, and co-expressing both mutants abolished the modifications, suggesting that RAP80-ub are predominant K48 and K63 conjugates (Supplementary Fig. S3C). Moreover, co-expressing a K48/K63-only species partially supported RAP80-ub in *70KO* cells, while the individual K48-only or K63-only versions failed, suggesting that K48 and K63 conjugates are mutually dependent on RAP80 modification.

To define the lysine residuals subjected to ubiquityl modification, we enriched slow-migrating fractions of Flag-RAP80 (>90 kDa) by immunoprecipitation and performed LC-MS/MS analysis (Supplementary Fig. S3D). Peptide fragment analysis identified six lysine candidates, including lysine 75 implicated in K63-conjugation on RAP80 [27] (Supplementary Fig. S3E). We generated a *RAP80* mutant containing *K > R* missense at K9, K31, K75, K245, K374, and K697 (coined as *6KR* mutant). *6KR* ectopic expression (*6KR*^EE^) in *70KO* cells diminished, if not abrogated, RAP80-ub levels in comparison to that on *WT* proteins (Fig. 3C). *6KR* maintained its functions in terms of DSB-loading and BRCA1 interaction (Supplementary Fig. S3F and G). Intriguingly, expression of *6KR*, but not *RAP80 WT*, restored RPA32 phosphorylation in *70KO* cells (Fig. 3D). Compared to si*RAP80*, diminishing RAP80 ubiquitination by *6KR*^EE^ maintained the level of DSB-associated RAP80 in *WDR70*-depleted cells (Fig. 3E). However, *6KR*^EE^ expelled 53BP1 from the 3.5–8 kb region, again not at 10 kb (Fig. 3F). Resultantly, pRPA32 levels within corresponding regions were specifically restored in *6KR*^EE^  *70KO* cells, which was reproduced in *HBx*-expressing L02 cells (Supplementary Fig. S3H). Therefore, ubiquitination (6Kub at K9, K31, K75, K245, K374, and K697) confers RAP80 the activity of resection barrier in phase I, in addition to the physical presence of RAP80 at DSB vicinity.

We next examined whether 6Kub exclusively collaborates with PTIP to establish resection barriers in phase I by expressing *6KR* in *70KO* cells while concomitantly depleting either *PTIP* or *RIF1*. Simultaneous treatment with phase II factor (si*RIF1*) and *6KR*^EE^ restored the pRPA32 IRIF and thoroughly recovered long-range resection up to 10 kb in *70KO* cells, resembling the effect of si*53BP1* (Fig. 3G and Supplementary Fig. S3I). This was not observed in the combination of si*PTIP* and *6KR*^EE^, suggesting that the 6Kub event regulates PTIP, but not RIF1. Indeed, *6KR*^EE^ diminished the persistent IRIF of PTIP in *WDR70*-defective cells, rather than that of RIF1 (Fig. 3H). ChIP assay supported that *6KR*^EE^ prevented DSB-binding of PTIP at 3.5–6 kb in *70KO* cells to an extent comparable to that of si*53BP1* and si*RAP80* (Fig. 3I). In contrast, RIF1 accumulation at 8–10 kb was not obviously affected by *6KR*^EE^ (Fig. 3J). These results were reproduced in *HBx*-expressing L02 cells, where combined treatment of si*RIF1*/*6KR*^EE^ or si*RIF1*/si*PTIP* fully rescued its defect in distal resection (Supplementary Fig. S3J). Importantly, 6Kub/PTIP- and ADRM1/RIF1-mediated sectional ssDNA processing were reproduced at two additional DSB sites in euchromatin loci of H2AZ and KYAT3 (Supplementary Fig. S4A and B), indicating that this bipartite mechanism is generally utilized in DSB repair instead of a site-specific event. We thus propose that 6Kub-mediated stabilization of 53BP1/PTIP in phase I stalls long-range resection, which is subjected to the displacement via CDW19S.

### RAP80 ubiquitin status determines the phase I loading of BRCA1

**Figure 4.**
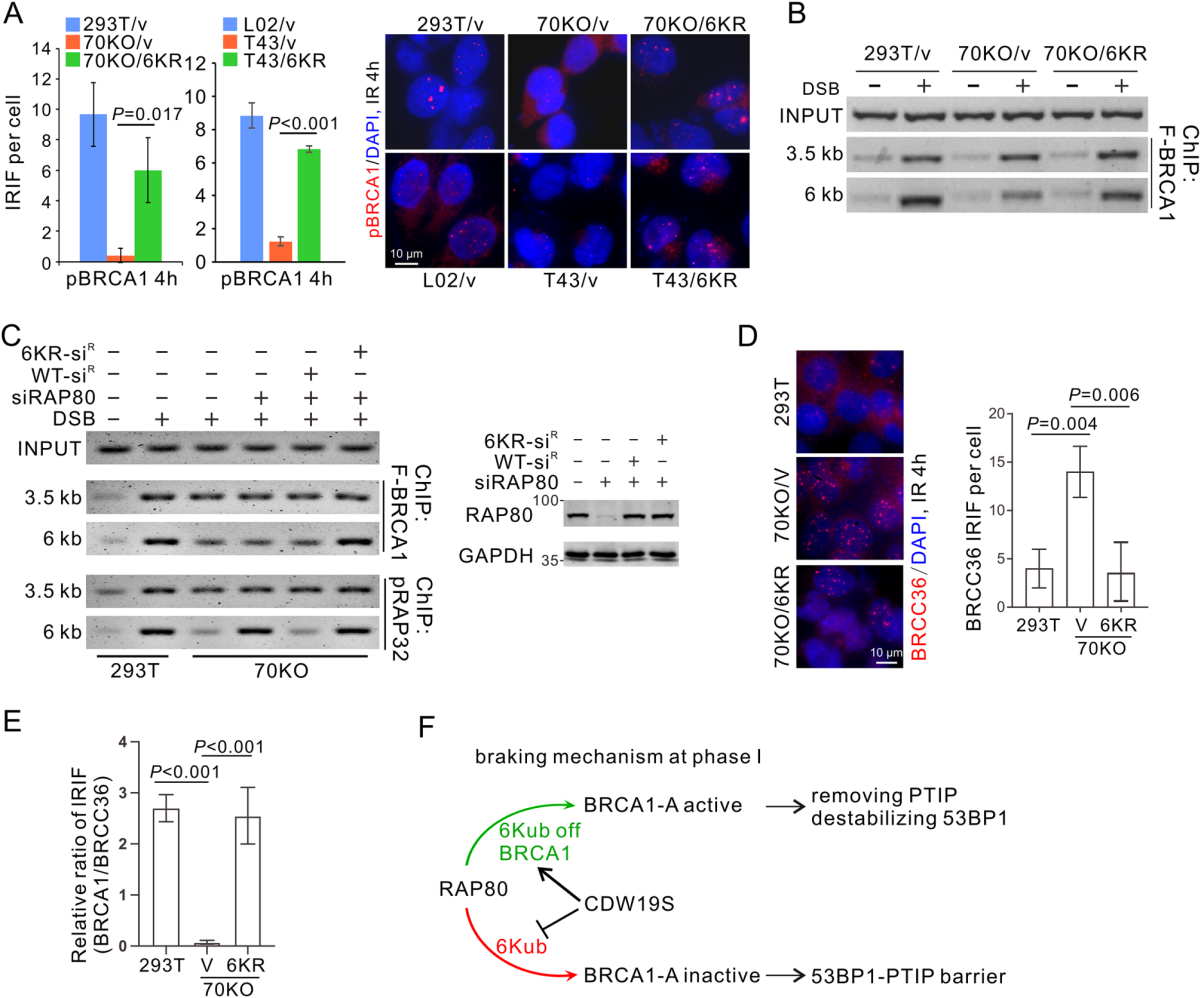
6Kub-free RAP80 implements the assembly of BRCA1–A complex. (**A**) IRIF formation of pBRCA1 in 293/*70KO* and L02/T43 cells. (**B**) ChIP assays for Flag-tagged BRCA1 in indicated cells. (**C**) ChIP assays for Flag-BRCA1 and pRPA32 in indicated cells. siRNA-resistant (si^R^) version of RAP80 *WT* or *6KR* was co-transfected with *siRAP80* into *70KO* cells. Immunoblotting of RAP80 is presented to show comparable expression between ectopic and endogenous *RAP80*. (**D**) Representative images and quantifications of BRCC36 IRIF in indicated cells with *6KR* expression or not. (**E**) The relative ratio of pBRCA1/BRCC36 IRIF was compared in *70KO* cells in the presence of *6KR* or not. (**F**) Schematic showing 6Kub provides a hesitation signal at phase I by suspending BRCA1–A assembly. Histograms in this figure are presented as mean ± s.d. *P*-values were calculated by a two-way ANOVA test.

While si*RAP80* is able to disrupt inhibitory 53BP1 in *70KO* cells, BRCA1 loading was not rescued (cf. Fig. 2). On the contrary, *6KR*^EE^ could effectively recover pBRCA1 IRIF in *70KO* and T43 cells (Fig. 4A), an impact supported by ChIP assay where BRCA1 recruitment was revitalized at 6 kb distal in *70KO 6KR*^EE^ double mutant (Fig. 4B). Consistently, complementing *RAP80*/*WDR70* co-depleted cells with siRNA-resistant *6KR* mutant, rather than wild-type *RAP80*, was able to restore the recruitment of both pRAP32 and BRCA1 (Fig. 4C). These data document the non-ubiquitinable nature of *6KR* enabling BRCA1 loading to DSBs that subsequently dqestabilizes 53BP1 in phase I, evoking an explanation concerning the dual roles of RAP80 in both inhibiting and activating BRCA1–A complex.

BRCA1–A complex consisted of RAP80, BRCC36, Abraxas, BRCC45, BARD1, MERIT40, and BRCA1 responds in the early stage of DSB repair [28]. Resembling RAP80, IRIF of BRCC36 was accumulated in *70KO* cells (Fig. 4D). CDW19S defect generated a BRCA1–free A complex, as the IRIF ratio of BRCA1/BRCC36 was significantly decreased in *70KO* cells (Fig. 4E). The ratio was restored upon *6KR*^EE^ due to increased BRCA1 (cf. Fig. 4A) and decreased BRCC36 (Fig. 4E), indicative of 6Kub as a switch governing the assembly of full BRCA1–A complex.

In this scenario, RAP80-6Kub renders BRCA1–A complex partially assembled in the absence of BRCA1 *per se*. The function of BRCA1-free complex is suspended and can be activated until RAP80 deubiquitination and DSB engagement of BRCA1. Thus the dynamic conjugation and removal of 6Kub defines a transition apparatus for BRCA1–A activation, we termed as hesitation mechanism, to determine the holding and proceeding of long-range resection (Fig. 4F).

### 6Kub-dependent hesitation mechanism contributes to the quality of homology-dependent repair

**Figure 5.**
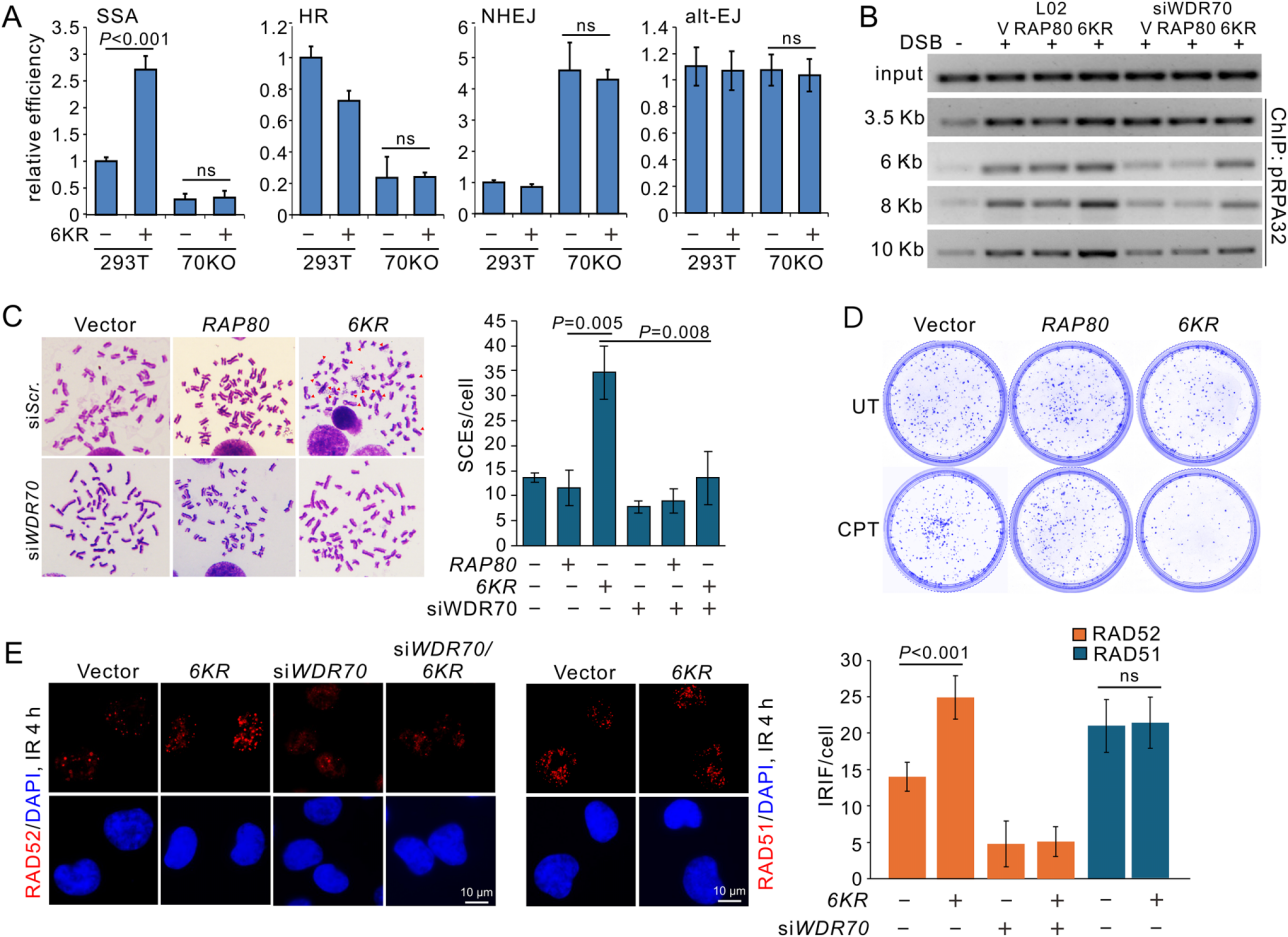
6Kub-mediated hesitation mechanism prevents hyperresection and mutagenic SSA repair. (**A**) Measurement of HR, SSA, alt-EJ, and NHEJ efficacies using the I-*Sce*I-DSB system in 293T and *70KO* cells transfected with *6KR*. (**B**) ChIP assays for pRPA32 in the indicated cells. (**C**) SCE analysis in L02 cells in the presence of RAP80 *WT* or *6KR*. Right: quantification for SCE frequency. (**D**) Colony assay for L02 cells transfected with empty vector, RAP80 *WT*, or *6KR*. Five days after seeding, cells were treated with CPT at 2 nM or left untreated. Survived colonies grew for 10 days and were visualized by Tanon MINI Space system. UT: untreatment. (**E**) IRIF and quantification for RAD52 (left) and RAD51 (right) in L02 cells with individual or combinatorial treatment with si*WDR70* and *6KR*^EE^. Histograms in this figure are presented as mean ± s.d. *P*-values were calculated by a two-tailed *t*-test.

To understand the role of the 6Kub-dependent hesitation mechanism, we further evaluated the ability of *6KR*^EE^ to alter the pathway choice of DSB repair in the context of CDW19S deficiency. No obvious activity changes regarding HR, SSA, NHEJ, and alt-EJ were observed in *70KO* cells transfected with *6KR* or a control plasmid (Fig. 5A). This suggests that the partial rescue of resection within a limited distance (3.5–8 kb, Supplementary Fig. S5A) is insufficient to activate homology-dependent repair, which requires phase II resection (beyond 8 kb). Surprisingly, *6KR*^EE^ provoked conspicuous hyperactivation of SSA accompanying reduced HR in wild-type 293T cells (Fig. 5A). In these cells, undue SSA concurred with excessive ssDNA production, as evidenced by increased RPA32 phosphorylation relative to cells transfected with RAP80-WT or vector plasmids (Supplementary Fig. S5B). Consistently, *6KR*^EE^ induced more intensified pRPA32 IRIF frequently coincided with non-denaturing signals of BrdU, consolidating superfluous production of ssDNA upon lack of 6Kub signals (Supplementary Fig. S5C). The unrestricted ssDNA generation was further confirmed by ChIP analysis in wild-type L02 cells showing enhanced pRPA32 deposition in both phases upon *6KR* expression, where the signals were diminished by *WDR70* ablation (Fig. 5B). Consequently, L02 cells with *6KR* expression, rather than WT RAP80, displayed a burst of sister chromatid exchange (SCE) that was suppressible by si*WDR70* (Fig. 5C). This hyper-recombination is detrimental to cell viability, as L02 cells expressing *6KR* exhibited reduced viability and were hypersensitive to genotoxic insults including CPT (Fig. 5D).

The above data indicate that the proceeding of resection into phase II is under the retroverse control of 6Kub in phase I, otherwise it could jeopardize cells to toxic SSA repair. One would expect that WT cells expressing *6KR* are pre-determined to overactivate RAD52, the main player of SSA [29]. Indeed, RAD52 IRIF but not RAD51 displayed a significant increase in *6KR*-expressing L02 cells (Fig. 5E), and RAD52 was indispensable for *6KR*-induced hyperactive SCE (Supplementary Fig. S5D). Thus, RAD52-mediated SSA underlies the hyper-recombination in *6KR*^EE^ cells, a process also requires CDW19S/WDR70’s activity to complete distal resection in phase II (cf. Fig. 5A and C). In contrast, prime factors of alt-EJ (PARP1 and Polθ) did not respond to *6KR* expression in IRIF assay (Supplementary Fig. S5E), reminiscent of its unaltered activity in the presence of *6KR* (cf. Fig. 5A). Nevertheless, CDW19S-mediated resection are apical events over homology-dependent recombination because ssDNA generation was not affected by silencing *RAD51* or *RAD52* expression in both wild-type and *6KR^EE^* cells (Supplementary Fig. S5F). These data reveal a crucial role of 6Kub-regulated resection brake, in that it prevents excessive ssDNA processing into distal region to overactivate toxic and unfaithful SSA repair, analogous to the loss of 53BP1 [30].

### Enzymatic activities of CDW19S enable the sequential removal of 53BP1 barriers

**Figure 6.**
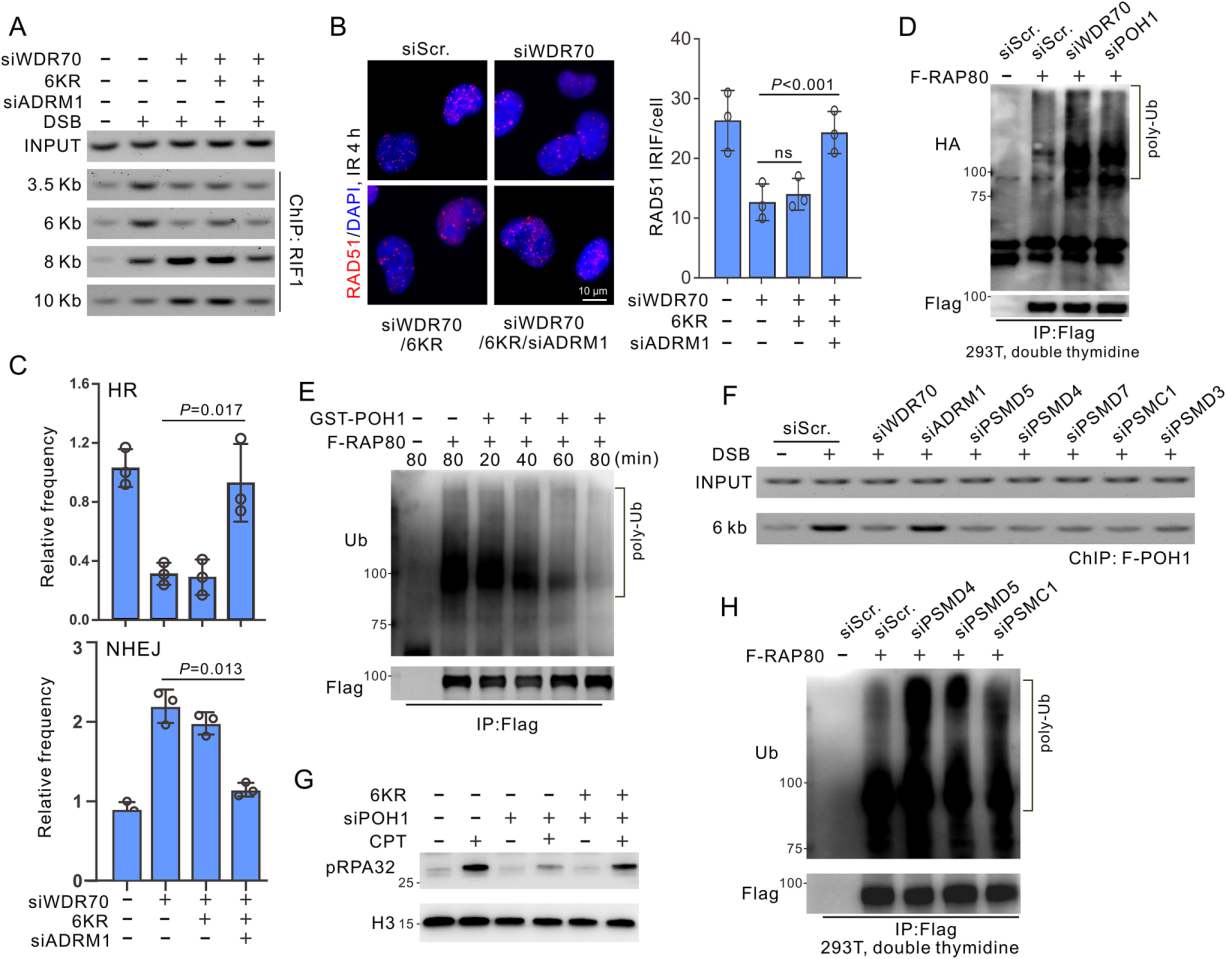
Distinctive CDW19S enzymes govern the removal of 53BP1 barricades in both phases I and II. (**A**) ChIP analysis for RIF1 in L02 cells upon introducing indicated siRNA or plasmids. (**B**) RAD51 IRIF assay in L02 cells subjected tosi*WDR70* and *6KR* expression. (**C**) Measurement of HR and NHEJ efficacies using the I-*Sce*I-DSB system in L02 cells treated with si*WDR70, 6KR*^EE^, and si*ADRM1*. (**D**) Polyubiquitinated RAP80 was detected in L02 cells transfected with si*WDR70*, si*POH1*, or not. (**E**) Flag-tagged RAP80 purified from 293T cells was incubated with 1 μg recombinant GST-POH1 for the indicated time point. Levels of RAP80 ubiquitination were detected by western blot using an antibody specific to ubiquitin. (**F**) ChIP assays for POH1 in L02 cells treated with indicated siRNA. (**G**) Immunoblotting of pRPA32 in L02 cells transfected with si*POH1* or *6KR*, concurrently treated with 2 μM CPT for 2 h, or not. (**H**) Polyubiquitinated RAP80 in L02 cells transfected with the siRNA against selected CDW19S components. All histograms are presented as mean ± s.d. *P*-values were calculated by a two-way ANOVA test.

*WDR70* defective cells are incompetent in both proximal and distal resection, suggesting that sufficient and quality production of ssDNA requires the function of CDW19S in both phase I and phase II. It is plausible that 6Kub-mediated 53BP1/PTIP stabilization in phase I (cf. Fig. 3I) and its joint action with ADRM1-dependent 53BP1/RIF1 establishment in phase II (cf. Fig. 1F) establish full-range resection barriers to block illegitimate HR. While *6KR*^EE^ overcame PTIP in phase I but failed to resolve excessive deposition of RIF1 in the context of CDW19S defects (cf. Fig. 3J), simultaneous ablation of *ADRM1* was capable of expelling RIF1 in phase II (Fig. 6A). Consistently, robust RAD51 IRIF were formed in si*ADRM1 6KR* si*WDR70* triple mutant relative to *WDR70*-depleted cells, however it remained low when *ADRM1* was competent (*6KR* si*WDR70* double mutant) (Fig. 6B). Resultantly, balanced repair activities (HR versus NHEJ) were restored in triple but not in double mutant (Fig. 6C).

It is notable that all factors in phase I, including BRCA1, RAP80, BRCC36, *et al*., emerged in higher metazoan species but are absent from lower single-cell eukaryotes. On the contrary, machinery constituted of CRL4^WDR70^, RIF1, ADRM1 (and the conserved K99 residual [19]), and 19S RP (framework for CDW19S) is conserved throughout eukarya. We propose that phase I was evolved from the basic phase II apparatus to deal with ever increasingly elaborated chromatin organization in metazoans.

We speculate that the phase I 6Kub event is also rid off by an integral enzyme of CDW19S. We investigated POH1, the core deubiquitinase of 19S RP known to function in DSB repair [19, 31, 32], and a promising candidate to scrape 6Kub from RAP80. *POH1* loss caused higher levels of RAP80 ubiquitination phenocopying si*WDR70* (Fig. 6D). Using an *in vitro* deubiquitination assay, we demonstrated that recombinant POH1 could effectively remove ubiquitin conjugates off RAP80 purified from *70KO* cells (Fig. 6E). si*POH1* resulted in increased 53BP1 levels at later time points (8 h) following IR treatment but lacked an impact at 30 min (Supplementary Fig. S6A), indicating that POH1 regulates the removal of 53BP1 barriers for extensive resection by targeting RAP80. In support of this, POH1 assayed by ChIP was enriched at phase I segments (Fig. 6F), and loss of which caused a resection defect at the same region, indicative of its connection with the RAP80 function (Supplementary Fig. S6B). Further, *6KR*^EE^ rescued the resection defect in *POH1*-deficient cells (Fig. 6G), strengthening that 6Kub is subjected to the deubiquitination activity of POH1 during DSB response in phase I.

We considered whether the complete assembly of CDW19S is necessary for POH1 activity. Upon ablating majority of CDW19S subunits, including CRL4^WDR70^ E3 ligase and its associated protein (PSMD5), anchor protein (PSMD4), MPN domain (PSMD7), AAA+ ATPases (PSMC1), and PCI factor (PSMD3) affected POH1 deposition at 6 kb distal to DNA breaks (Fig. 6F). Consistently, depleting these CDW19S factors also raised the level of RAP80-Ub species (Fig. 6H), correlating their function with POH1 and 6Kub events. Further, these data suggest that 6Kub deubiquitination via POH1 requires the complex integrity of CDW19S that attaches POH1 enzyme to DSB chromatin. Coherent to the function of ADRM1 in phase II (cf. Fig. 1F), the phase I DSB loading of POH1 did not rely on ADRM1 (Fig. 6F), and si*ADRM1* failed to restore POH1 deposition in *70KO* cells (Supplementary Fig. S6C), corroborating that 6Kub removal in phase I requires POH1 function but is independent of CRL4^WDR70^-ADRM1 regulation.

Taken together, we conclude that CDW19S provides separate enzymatic activities pivotal for the sequential removal of 53BP1/PTIP and 53BP1/RIF1 resection barriers. These events are not attributed to *ad hoc* and passive redistribution of repair factors, but are deliberately controlled and factor-driven processes.

## Discussion

Unlike error-prone NHEJ and SSA, HR is a high-fidelity repair pathway crucial for genomic stability. BRCA1 orchestrates the HR cascade by dynamic incorporation into distinct complexes [28]. The BRCA1–A complex, via its subunit RAP80, detects K63-linked ubiquitination at DNA breaks and initiates early HR signaling [15, 16]. Later, the BRCA1–C complex (BRCA1-MRN-CtIP) facilitates DNA end resection, a process also involving EXO1 and DNA2, to generate extended ssDNA for homology search [33]. The final step involves the BRCA1-BRCA2-PALB2-RAD51 complex, which catalyzes strand invasion and completes recombination [34, 35].

Our current data revealed a stepwise resection model in which distinct enzymatic components of CDW19S overcome RAP80 and 53BP1. POH1 initiates extensive resection by deubiquitinating RAP80-6Kub to enable full assembly of BRCA1–A and remove 53BP1/PTIP, and CRL4^WDR70^ takes over the relay to degrade ADRM1 that dictate 53BP1/RIF1 removal and full-range resection (see the “Graphical abstract” section). This two-phase resection mechanism not only facilitates the production of sufficient length and amount of ssDNA necessary for HR, but also offers pausing points to check the quality of ssDNA in each step.

While RAP80 recruits BRCA1–A complex in the initial stage, it also restricts resection by sequestering BRCA1 *per se* [17, 36]. Our study demonstrates the mechanism governing this dual function is the metabolism of 6Kub modification, which is hydrolized by POH1 to terminate the BRCA1 sequestration (Figs 4A and 6E). It is sound that BRCA1 engagement to BRCA1–A ends the occupancy of 53BP1/PTIP and the subsequent assembly of BRCA1–C complex containing CtIP/EXO1, thereby firing extensive DNA end resection. These findings identify 6Kub-POH1 regulation as an on–off switch that hands over BRCA1 to different BRCA1-containing complex, enabling the transition from early damage detection to active end processing.

The CDW19S complex assembled at DSB ends provides two major enzymatic activities: CRL4^WDR70^ that triggers ADRM1 degradation and the deubiquitinase POH1 targeting 6Kub. RAP80 deubiquitination is not carried out by roaming POH1 but requires POH1 decorated into fully assembled and break-associated CDW19S complex. A panel of CDW19S subunits, including *WDR70*, offers structural platforms rather than enzymatic activity for the DSB recruitment of POH1 (Fig. 6F). Consequently, CRL4^WDR70^ plays both structural role for POH1 recruitment and E3 ligase activity for ADRM1 degradation.

Distinct enzymatic activities of CDW19S expose a bipartite nature of long-range resection with respect to 53BP1. In the phase I region that is ~3.5–6 kb from the break site (in our current CRISPR-induced DSB locus), BRCA1 is excluded from the BRCA1–A complex upon 6Kub modifications, allowing the establishment of 53BP1/PTIP phase I barrier. Elimination of 6Kub by POH1-DUB allows resection proceeding into phase II (Fig. 3F and G). In segment II spanning 8–10 kb from the break, resection is suspended by joint actions of ADRM1 and 53BP1/RIF1, which temporarily pauses HR prior their removal via CRL4^WDR70^ (Fig. 6A–C). Interestingly, resection suspension in spatially distinct phases operates independently but in coordination to achieve qualified resection with respect of ssDNA length and amount, as evidenced by the synergistic effect of si*PTIP*-si*RIF1*, si*RIF1*-*6KR*^EE^, and *6KR*^EE^-si*ADRM1* in CDW19S defective cells (Figs 1B, 3G, and 6C). Apparently, these braking mechanisms allow the activation of key factors (BRCA1, RAD51) at appropriate timing, which is crucial for preventing toxic and mutagenic repair mechanisms.

The phasic model for end resection is evolutionarily sound. CRL4^Wdr70^, Crb2^53BP1^, and Rif1 are highly conserved in the fission yeast (*Schizosaccharomyces pombe*). We previously showed in *S. pombe* that CRL4^Wdr70^ is required to counteract the inhibition of resection by Crb2^53BP1^ within a distal region of 2–3 kb from DNA ends, however they do not contribute to processing at the very end of breaks (0.5 kb) [37]. Interestingly, ADRM1 and its lysine 99 (lysine 100 of Rpn13b in *S. pombe*) liable to CRL4^WDR70^ modification are also conserved in lower eukaryotes. We posit that phase II is the prototypic resection regulation common in eukarya. To deal with more complicated genome composition and regulation, higher eukaryotes evolve additional layer of phase I control represented by the BRCA1-RAP80 circuit to tighten the resection control.

It has been documented that DNA end resection is initiated by MRN/CtIP-dependent short-range resection, followed by extensive long-range resection mediated by EXO1 and BLM/DNA2. DNA2 only degrades 5′-terminated ssDNA and requires helicase partners (BLM or WRN) to unwind dsDNA. In contrast, EXO1 degrades dsDNA from 5′ ends but is sensitive to protein barriers [33]. Our current study has widened the scope of this process by showing the interplay of positive (CDW19S) and negative (PTIP, RIF1, and 53BP1) controls on EXO1 activation. Moreover, MRN-CtIP also plays nuclease-independent roles to hand over nucleolytic activity to EXO1 and DNA2, probably by providing suitable DNA structure and antagonizing barrier proteins [38–40]. However, CDW19S is not part of this toolbox because CRL4^Wdr70^ in *S.pombe* specifically promotes Crb2^53BP1^ removal and Exo1-mediated resection, but is irrelevant to the functions of Mre11, Ctp1^CtIP^, or Rqh1^BLM^ [37]. Therefore, CDW19S exerts specific regulation on EXO1 activity via removing resection roadblocks of 53BP1.

In summary, our work proposes a model in which CDW19S coordinates phasic processing of DNA breaks through its embedded distinct enzymatic modules. By sequential dismantling individual 53BP1 barrier complexes in spatially separated regions, CDW19S contributes to sufficient and tightly controlled end processing, thereby introducing appropriate repair mechanism to maintain genomic stability.

## Significance statement

DNA end resection is a labrynthic process involving a myriad of signals. The DSB-associated complex CDW19S orchestrates the stepwise removal of 53BP1 and its effector proteins via its integral enzymatic modules: POH1 deubiquitinase and CRL4^WDR70^ E3 ligase. In the initial phase, POH1 targets RAP80 K48/K63 ubiquitination, switching on BRCA1 recruitment and removing proximal 53BP1/PTIP barriers; in the second phase CRL4^WDR70^ catalyzes ADRM1 degradation to destabilize 53BP1/RIF1 at distal regions. These spatial separated but coordinated actions of CDW19S enable the sufficient production of ssDNA and avoidance of toxic repair intermediates.

## Supplementary Material

gkag681_Supplemental_Files

## Data Availability

The data that support the findings of this study are available from the corresponding authors upon reasonable request. Source data are provided with the paper.
