# Supplementary material for: CDW19S coordinates phasic end processing via distinct enzymatic activities
Source: Nucleic Acids Res. 2026 Jul 6;54(13):gkag681. doi: 10.1093/nar/gkag681 (PMC13335481; doi:10.1093/nar/gkag681)

Fig. 1C

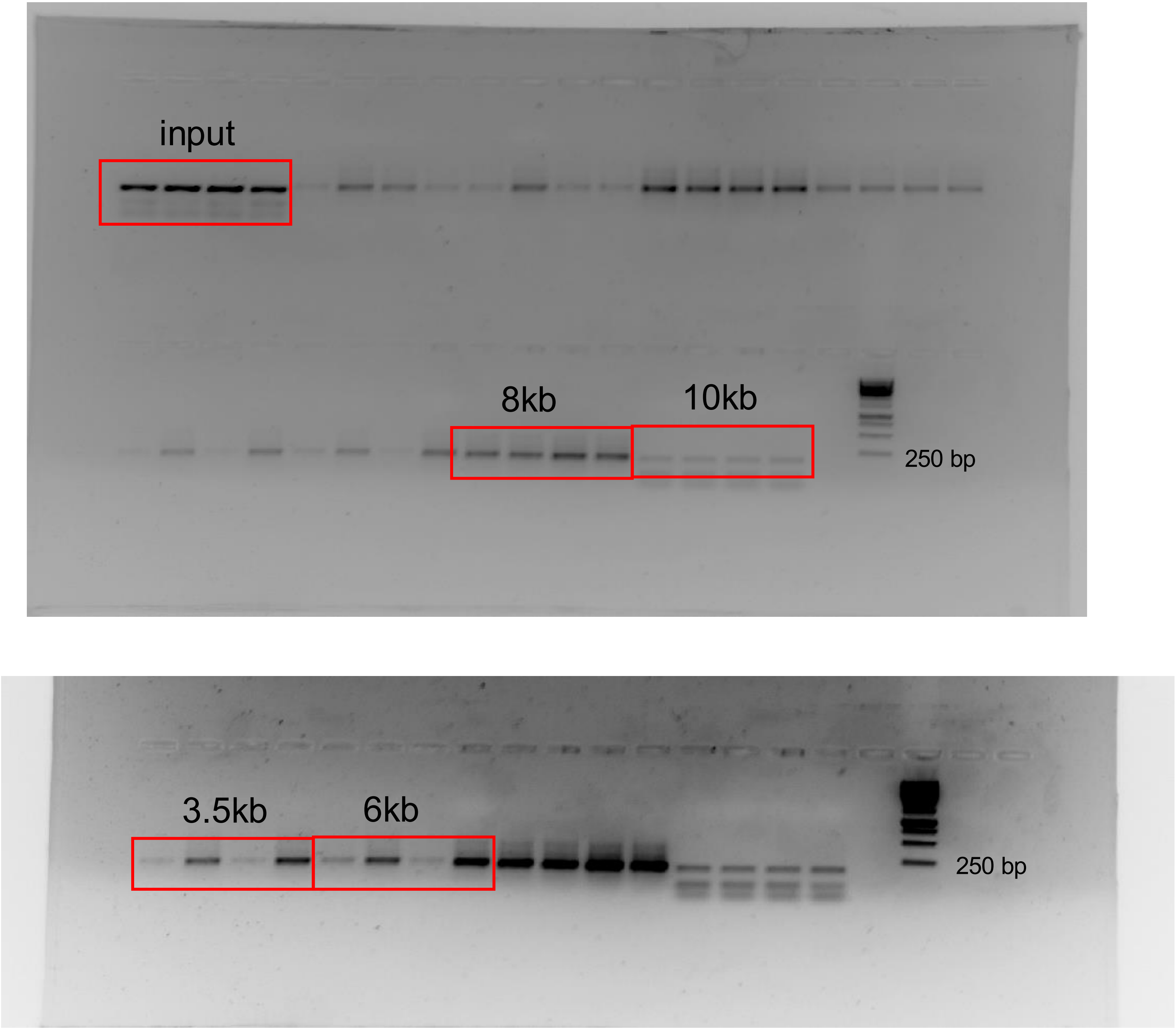

Fig. 1D

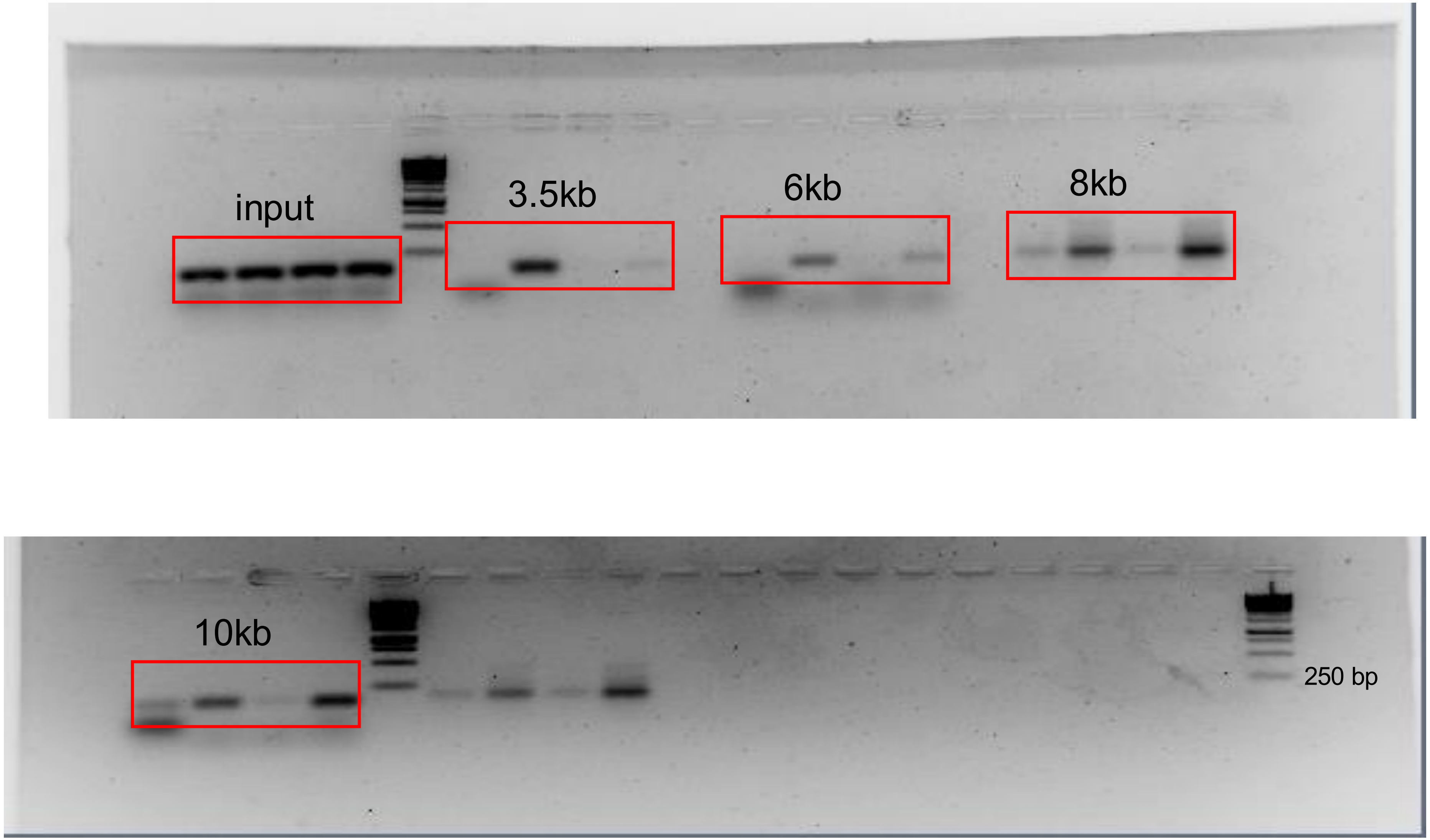

Fig. 1F

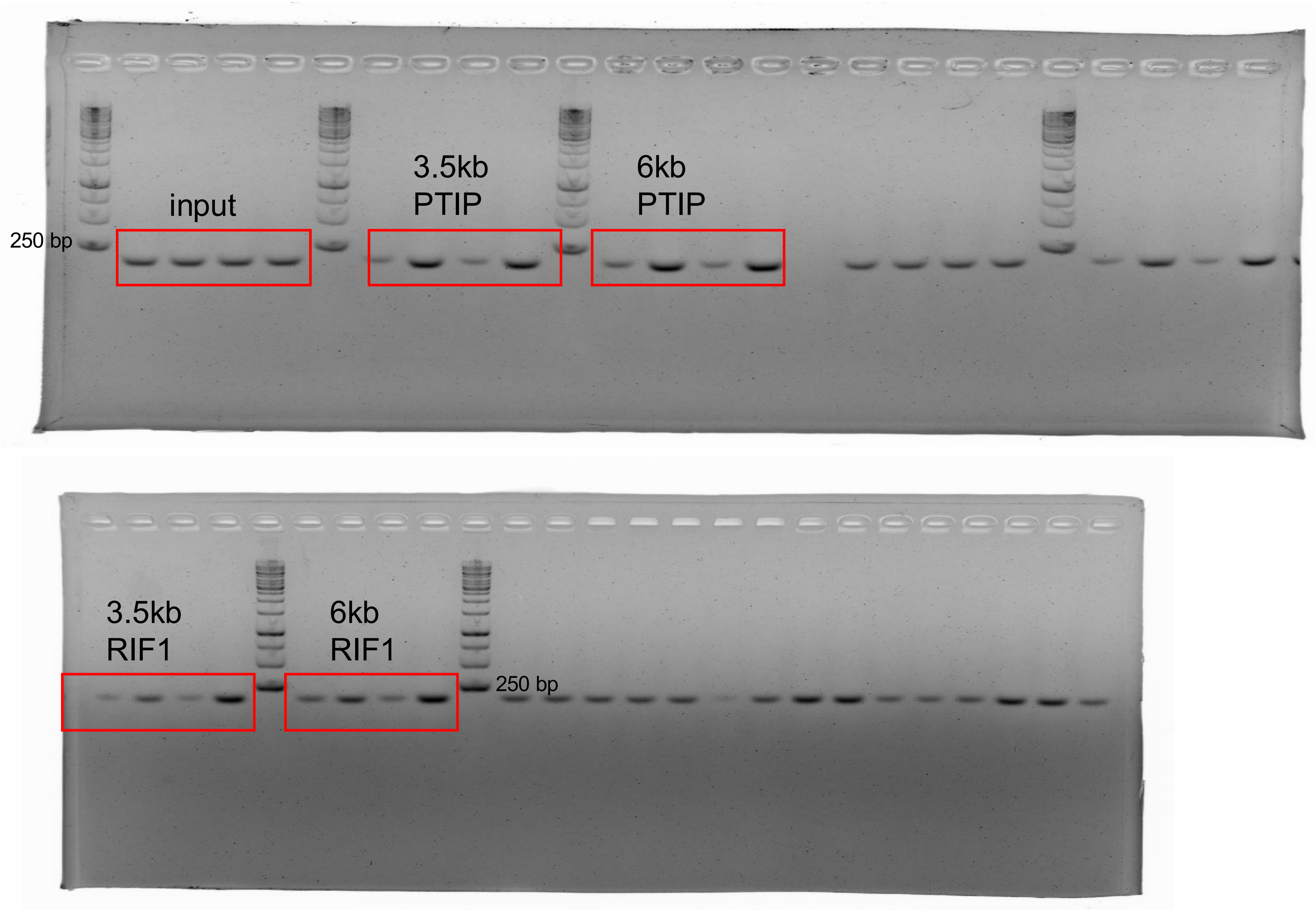

Fig. 2B

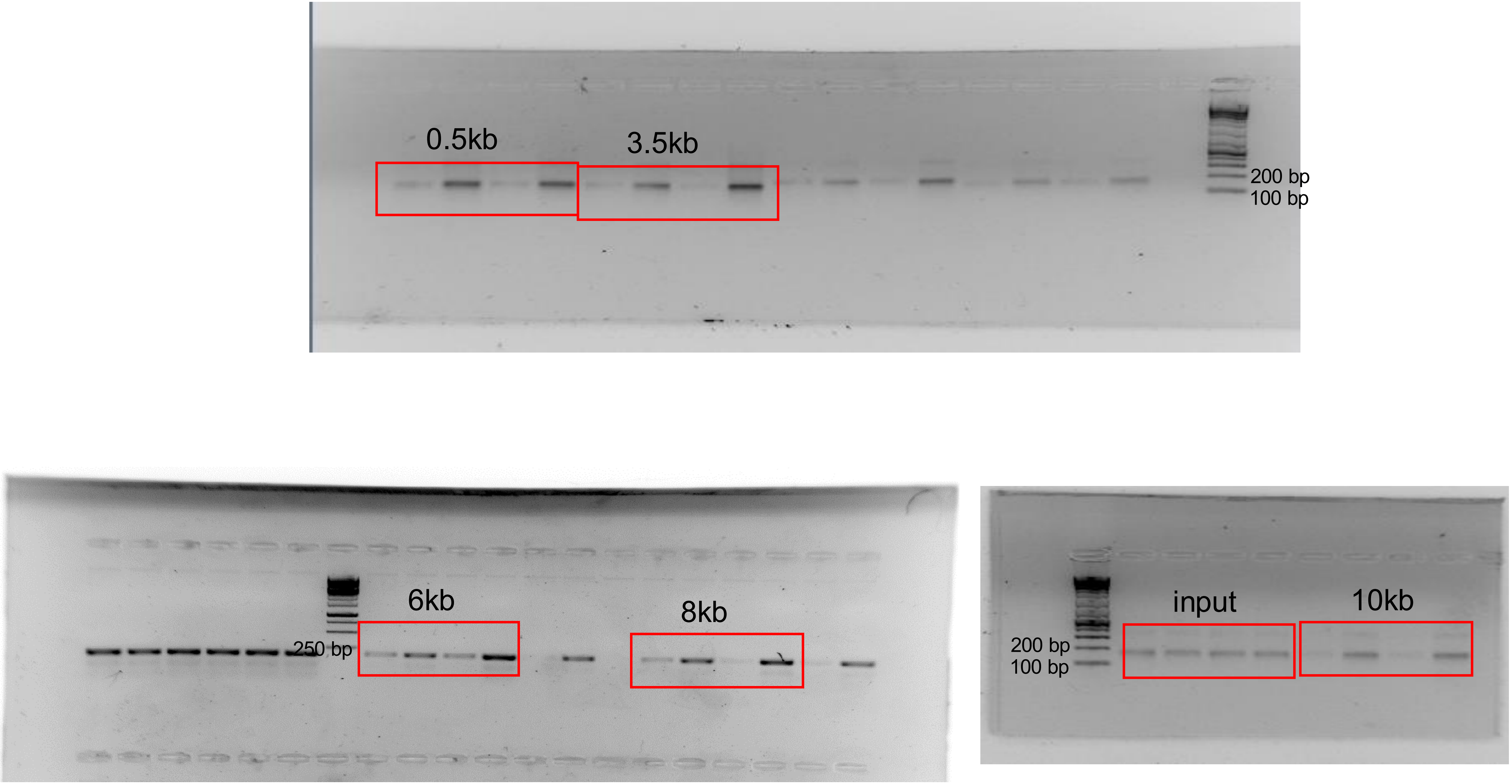

Fig. 2D

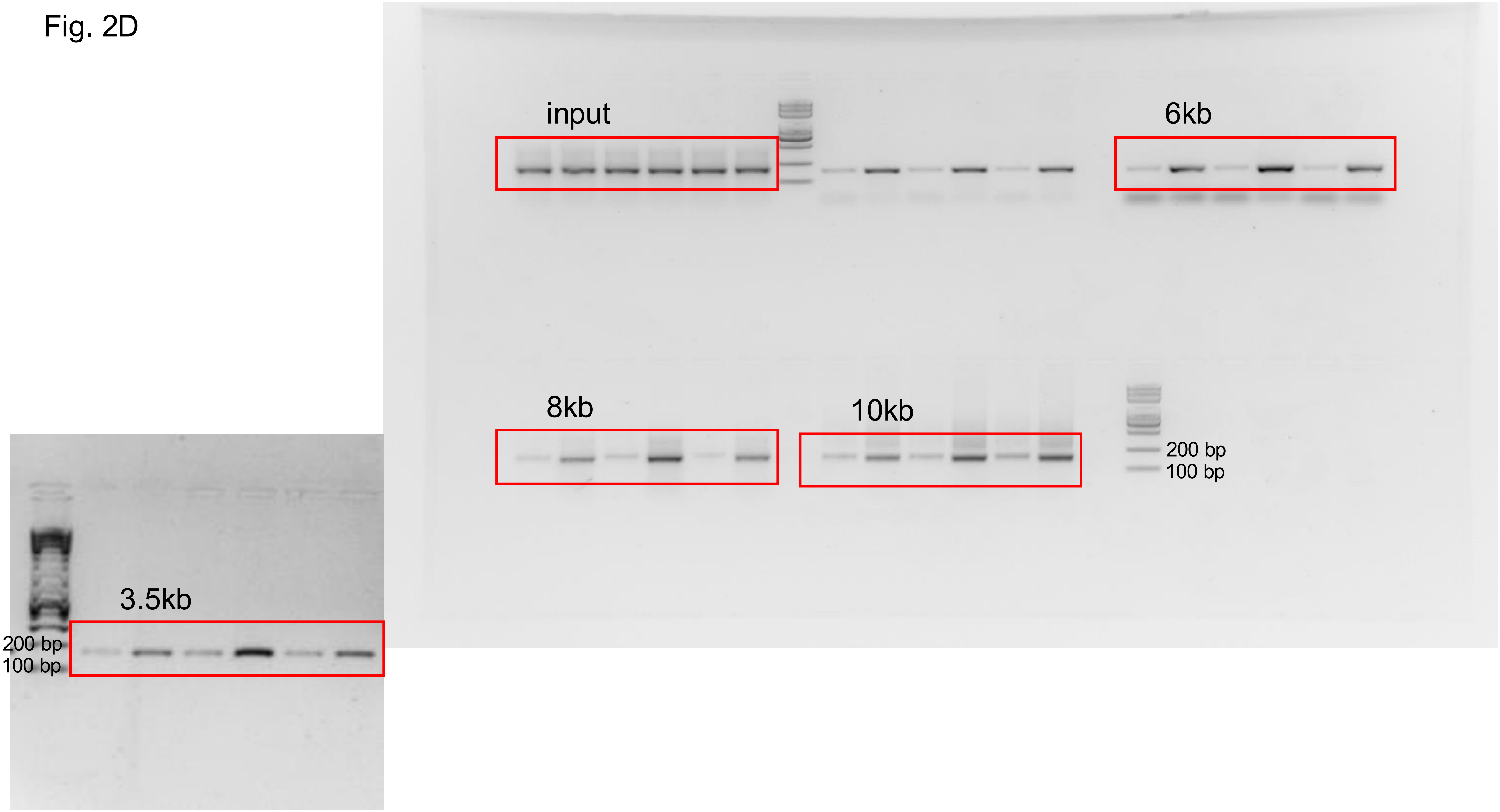

Fig. 2E

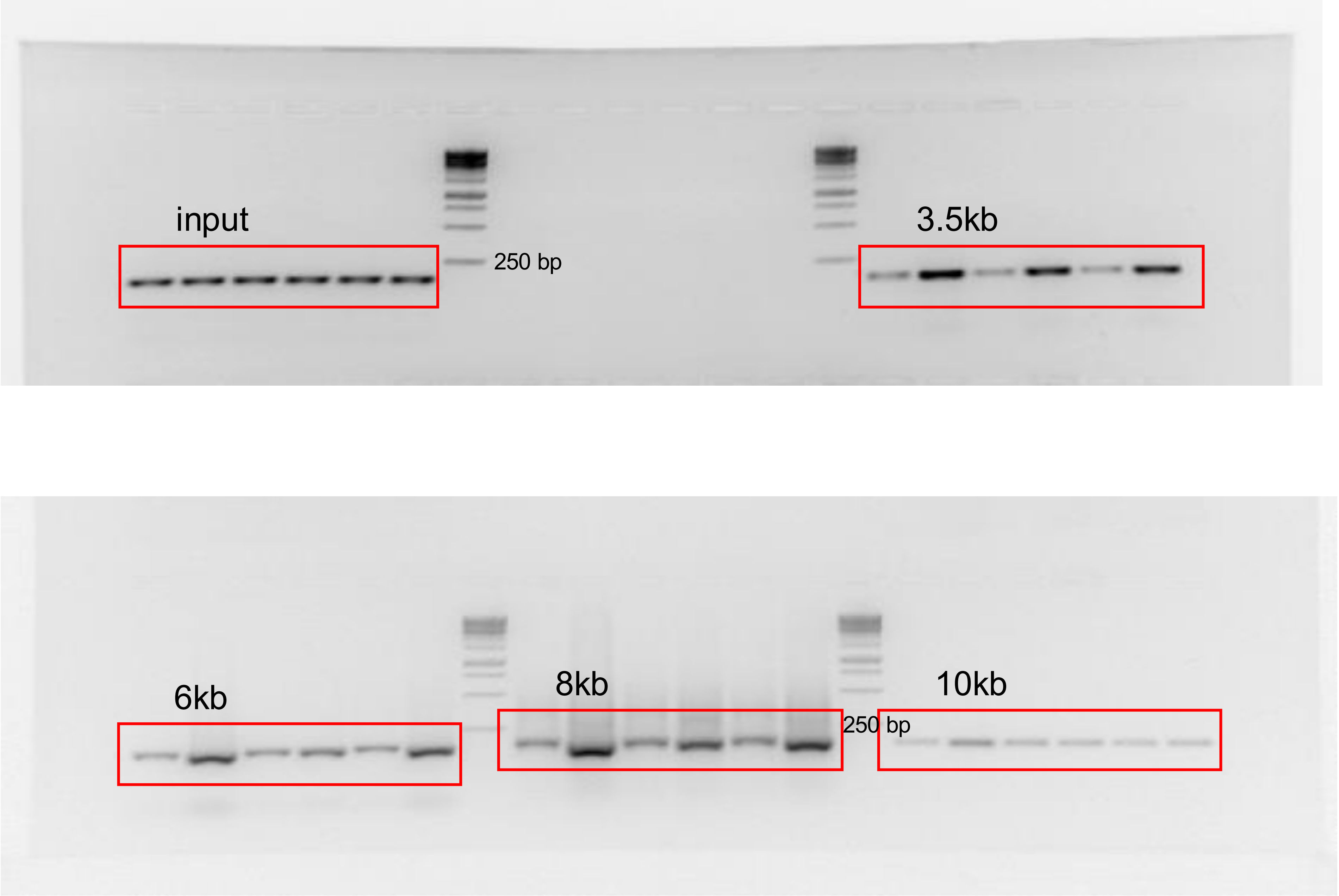

Fig. 2I

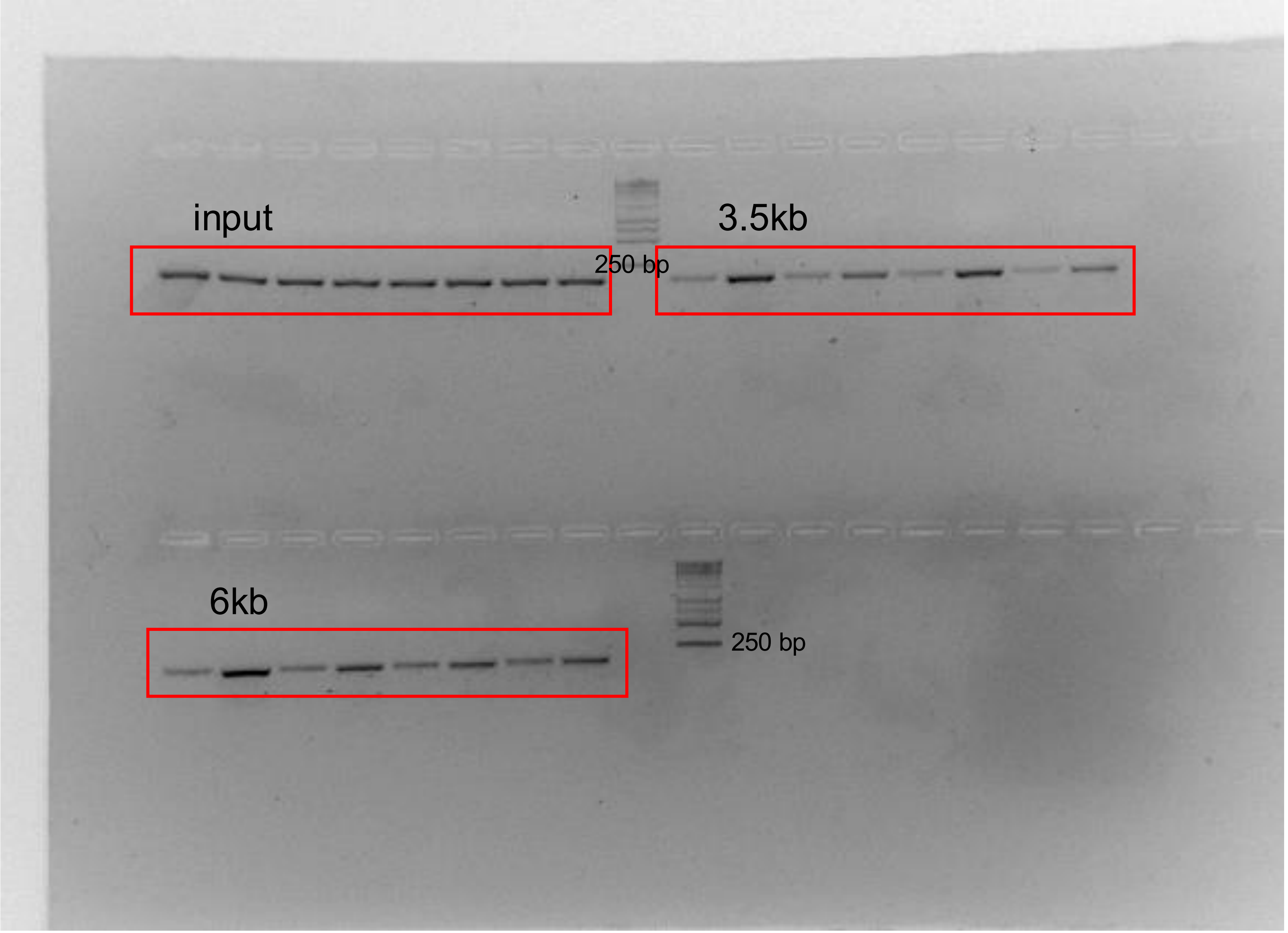

Fig. 2J

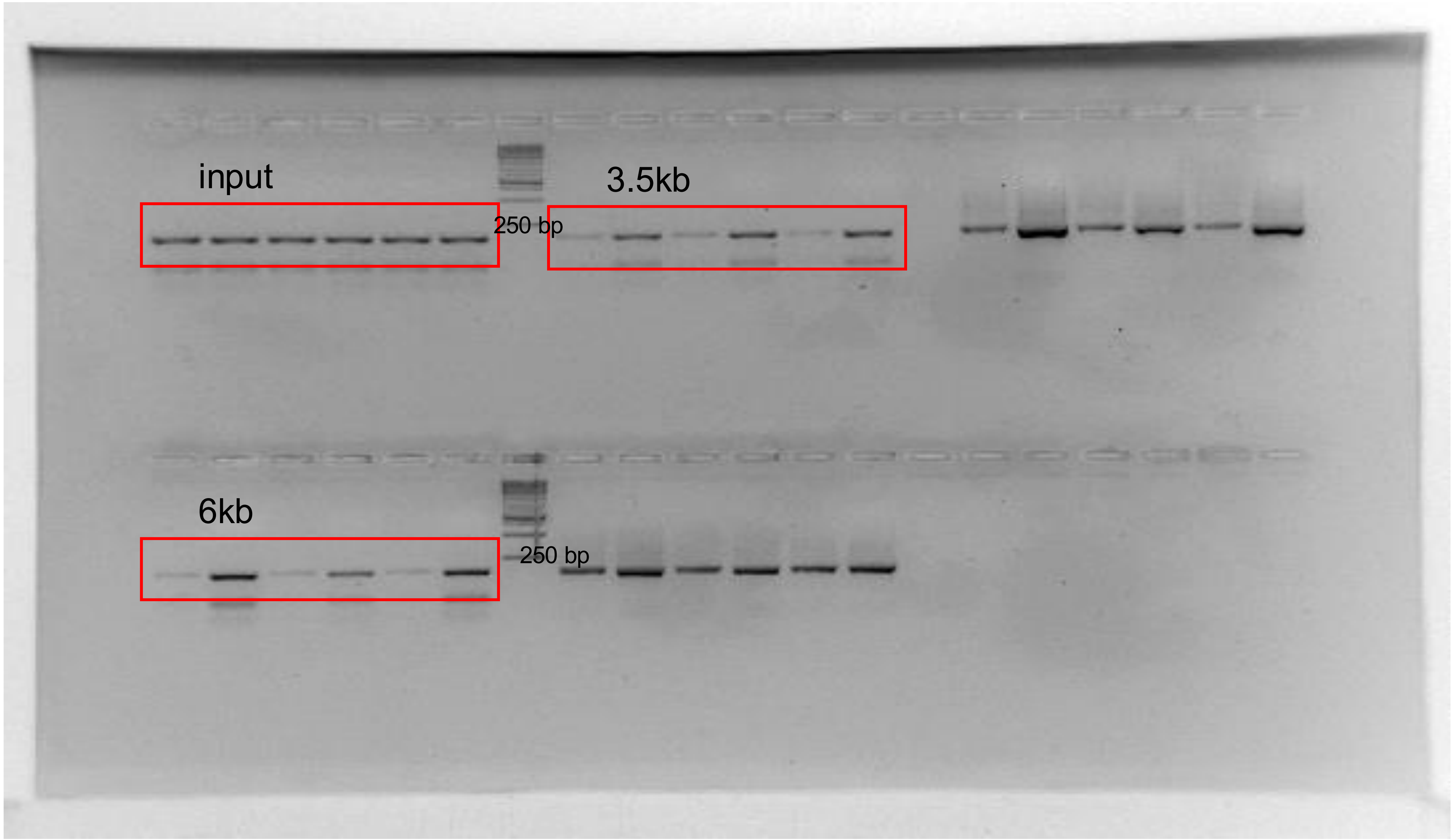

Fig. 3A

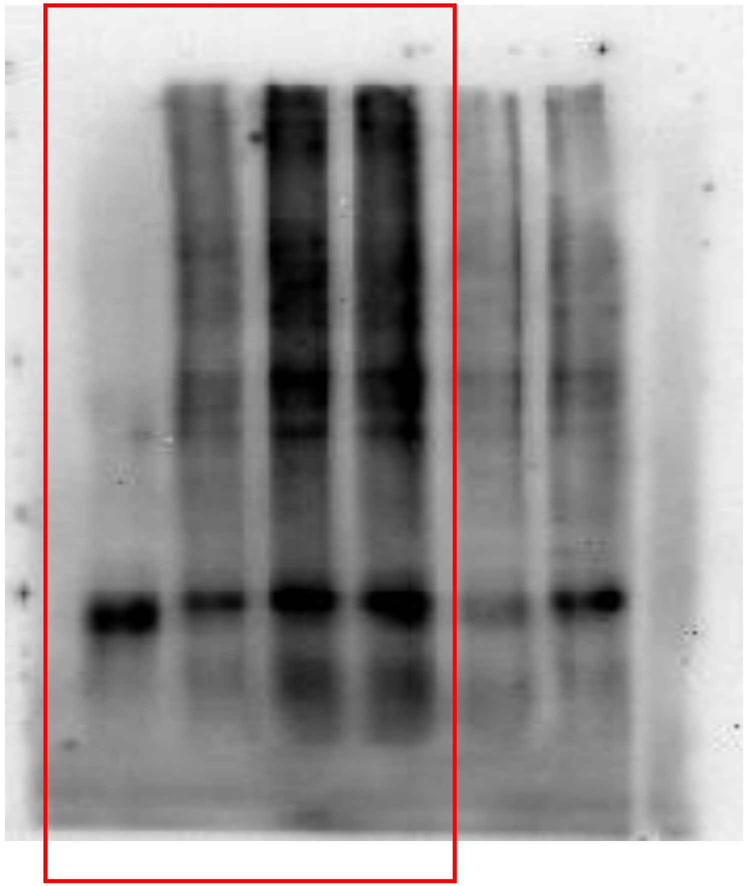

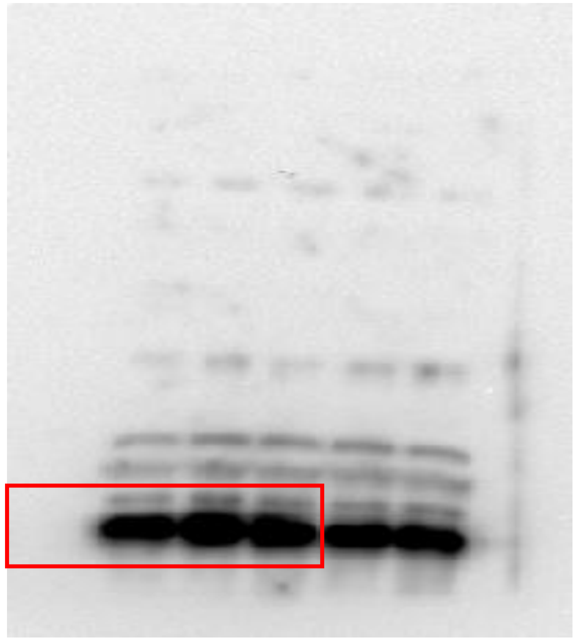

Fig. 3B

HA

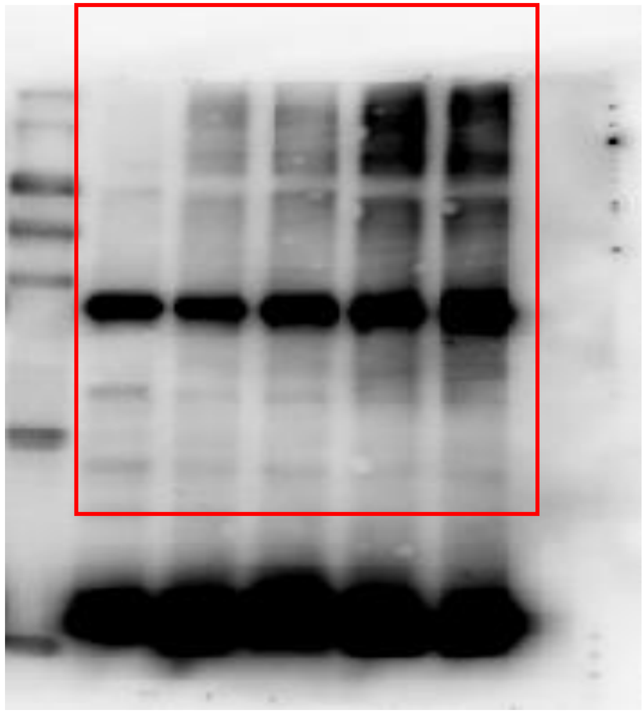

Flag

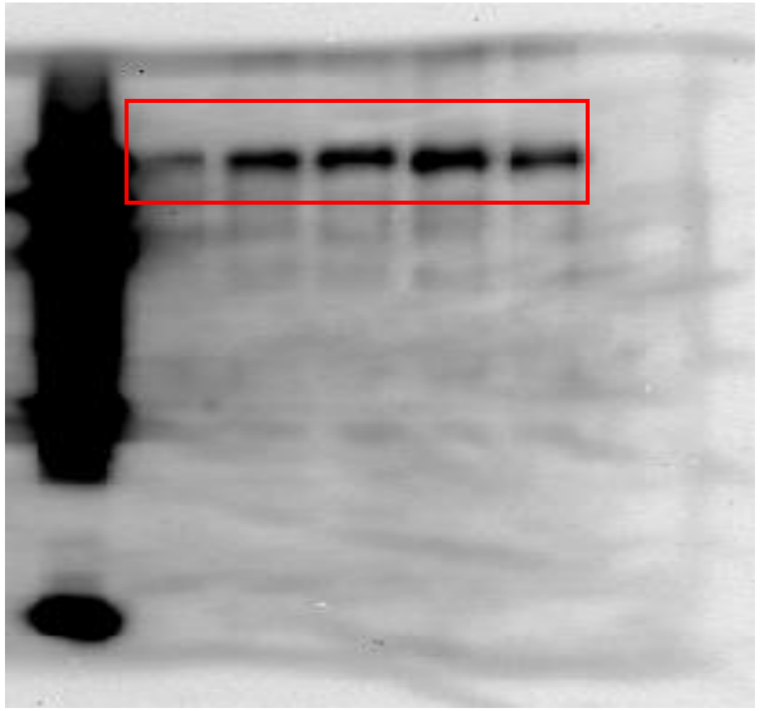

Fig. 3C

HA

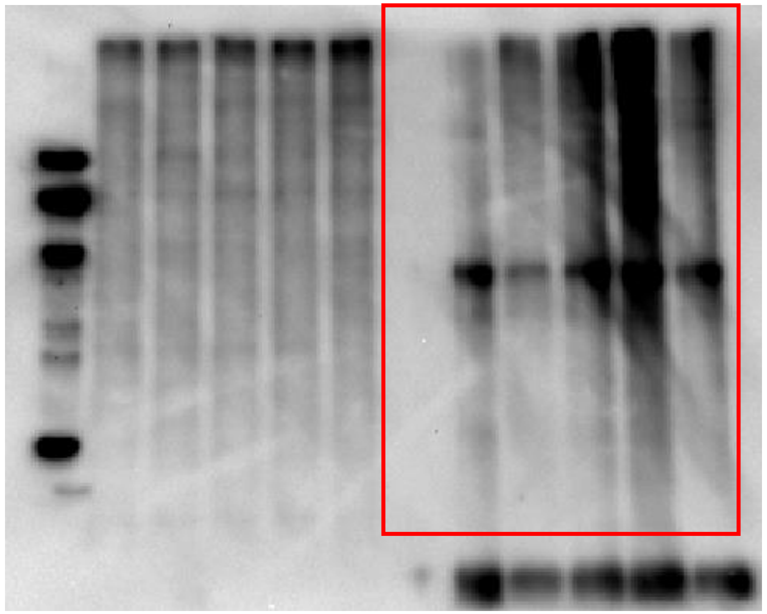

Flag

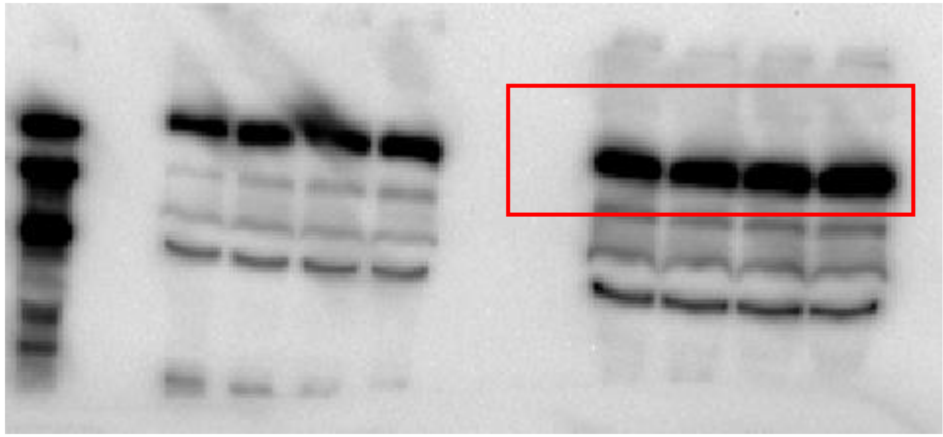

Fig. 3D

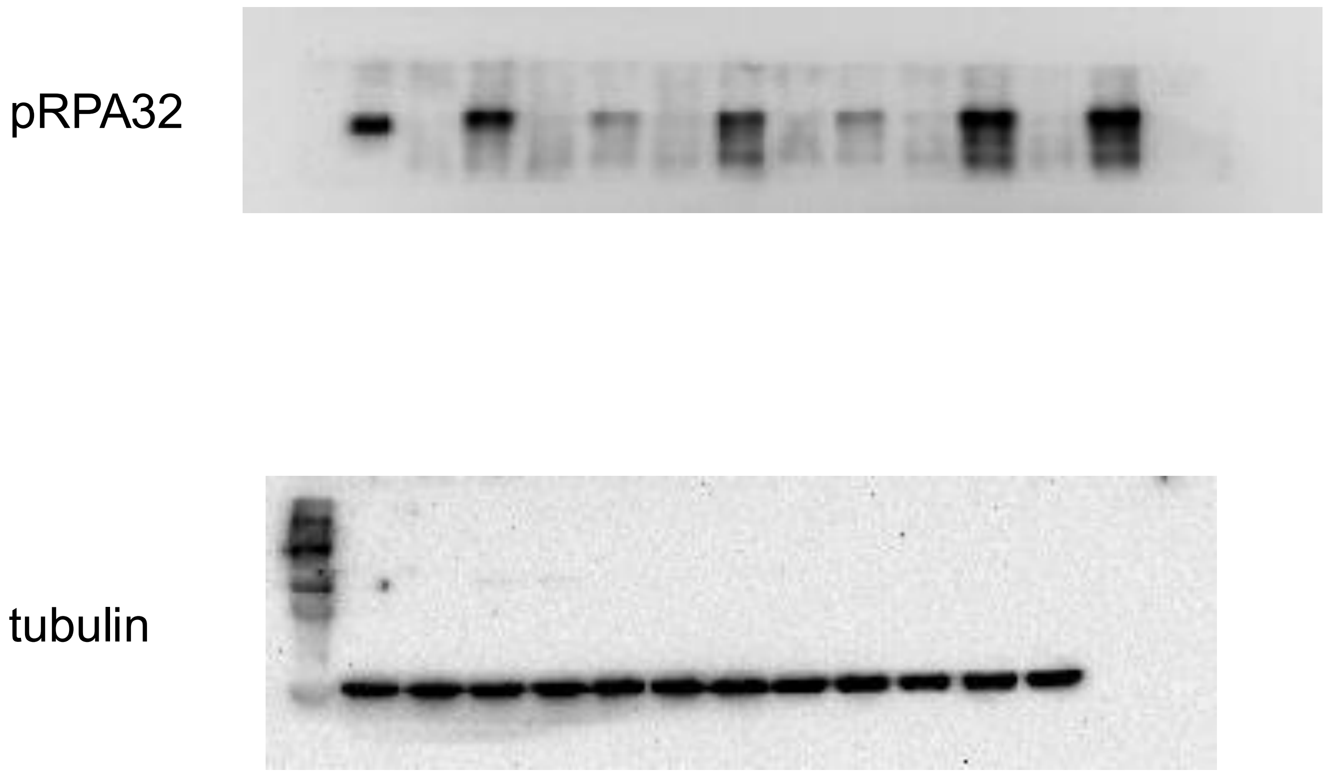

Fig. 3E

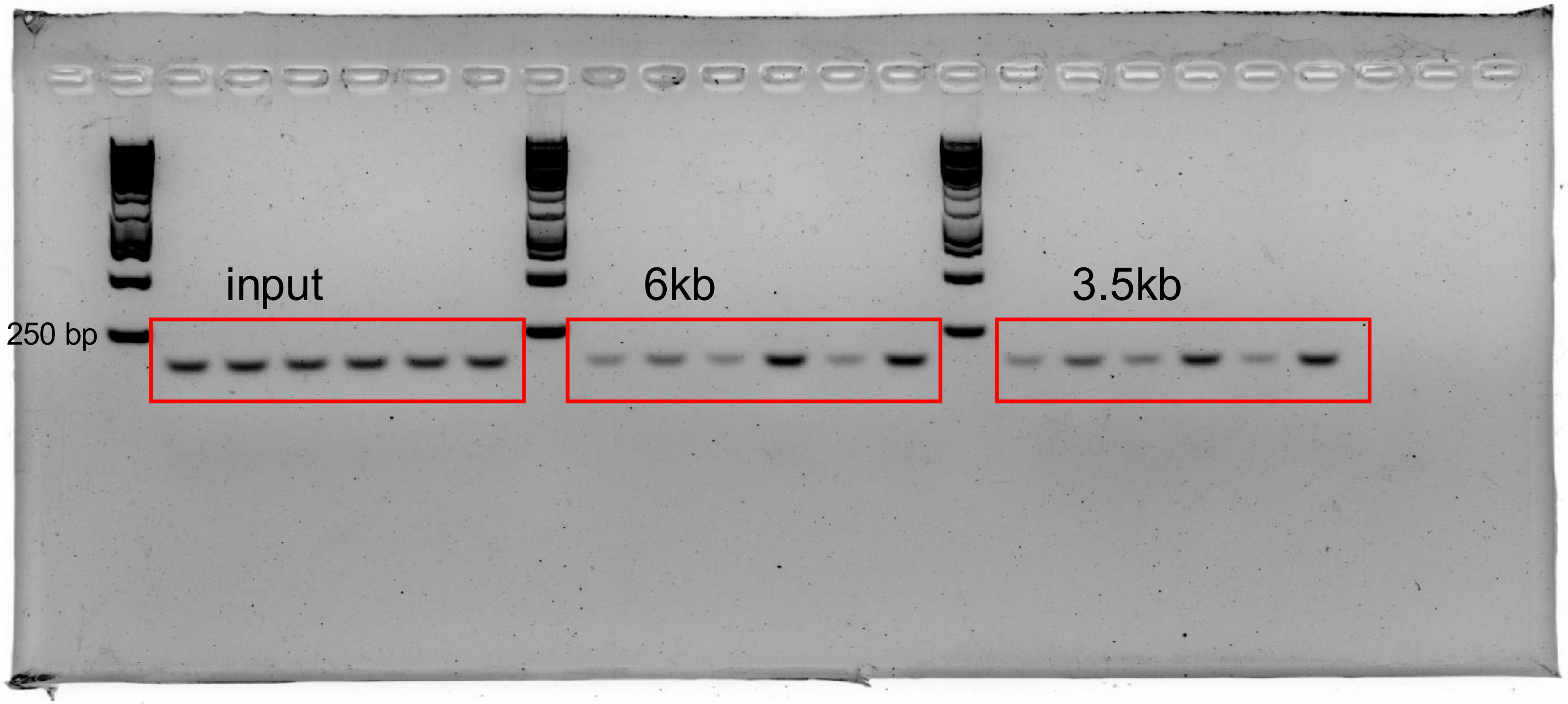

Fig. 3F

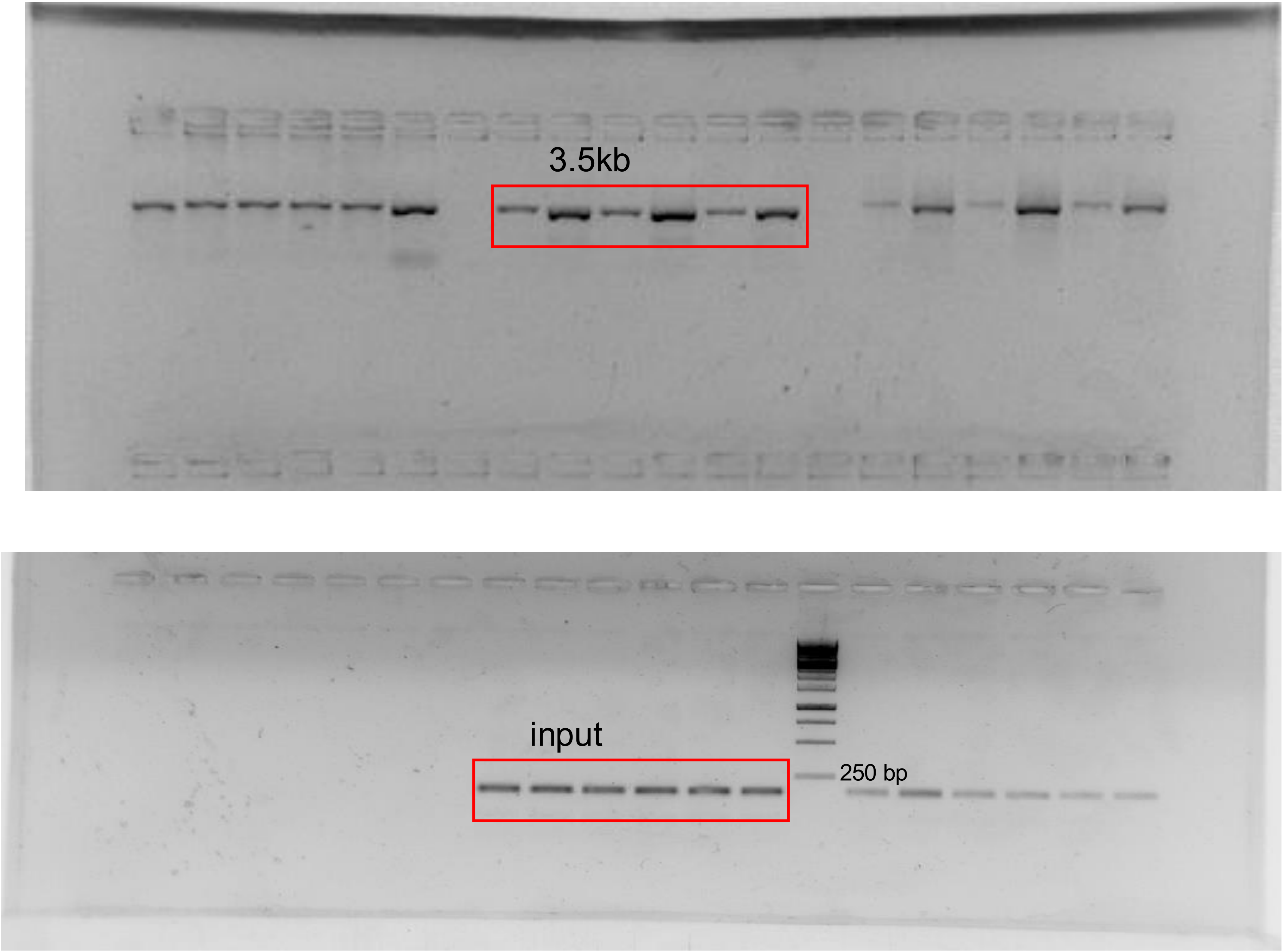

Fig. 3F

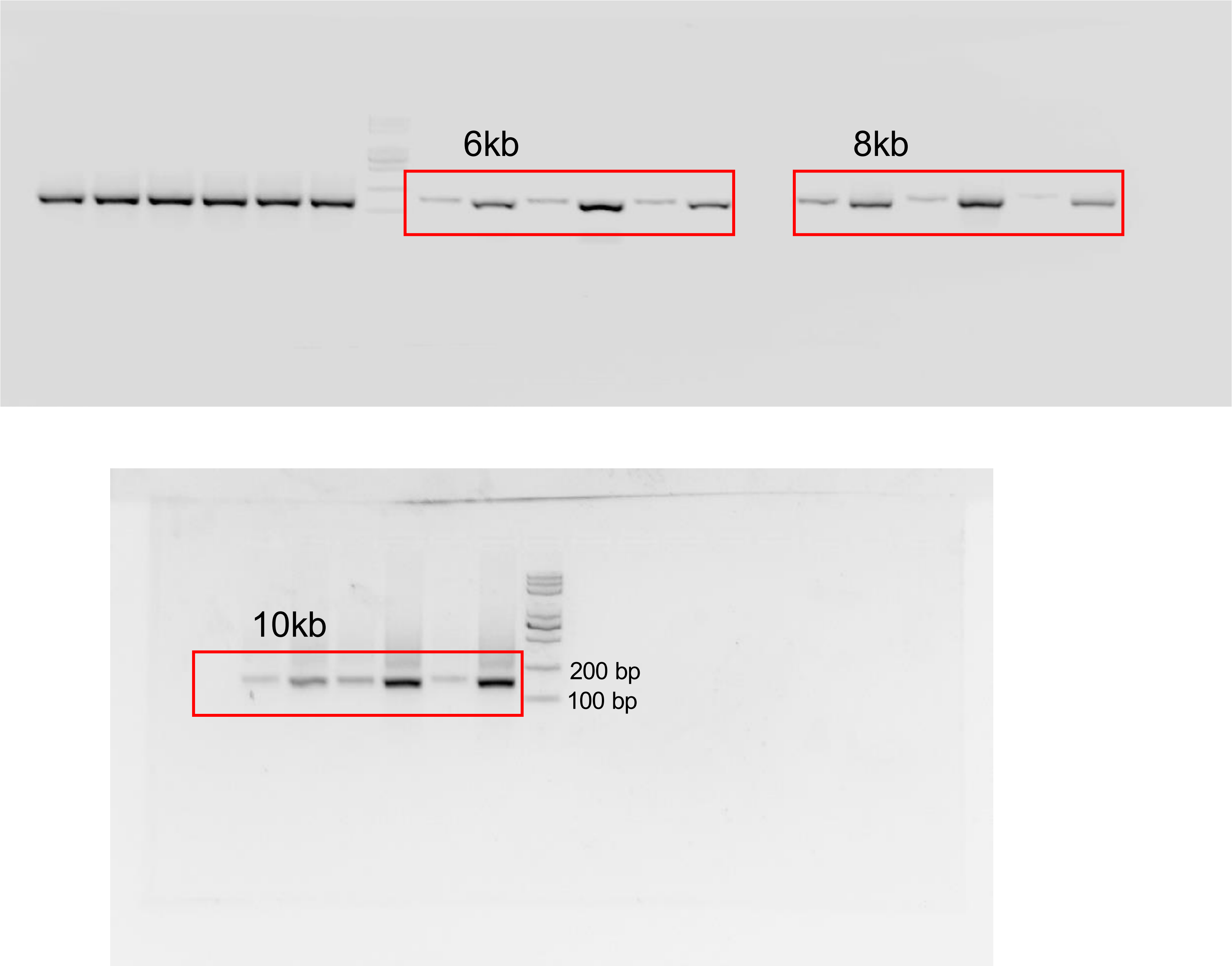

Fig. 3G

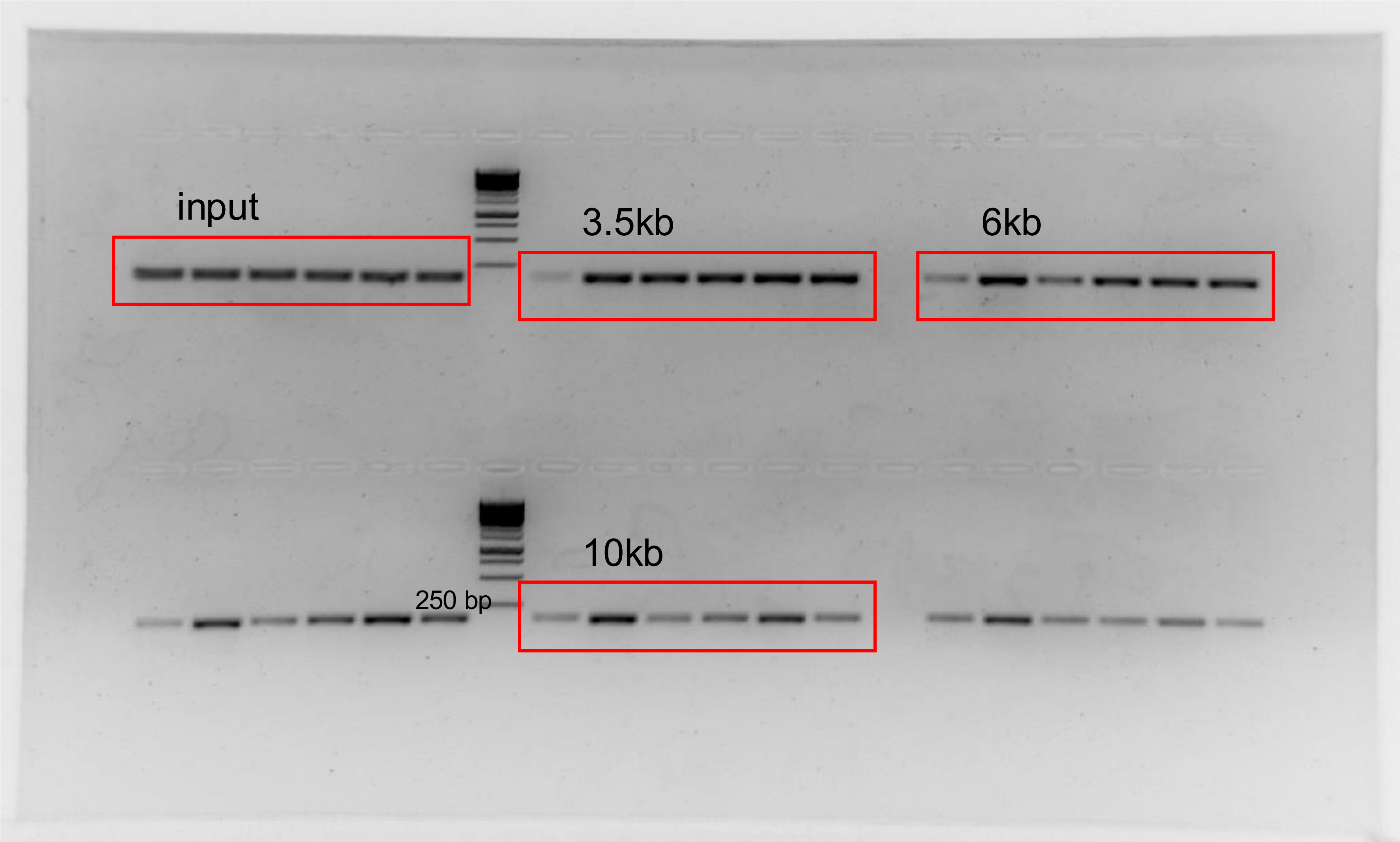

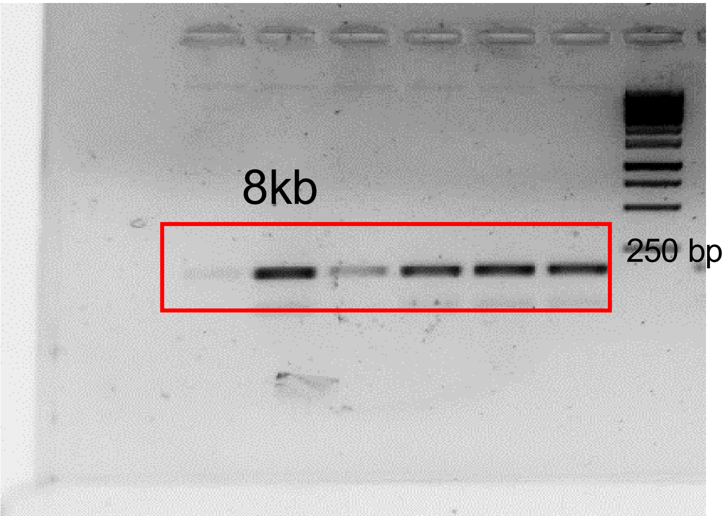

Fig. 3I

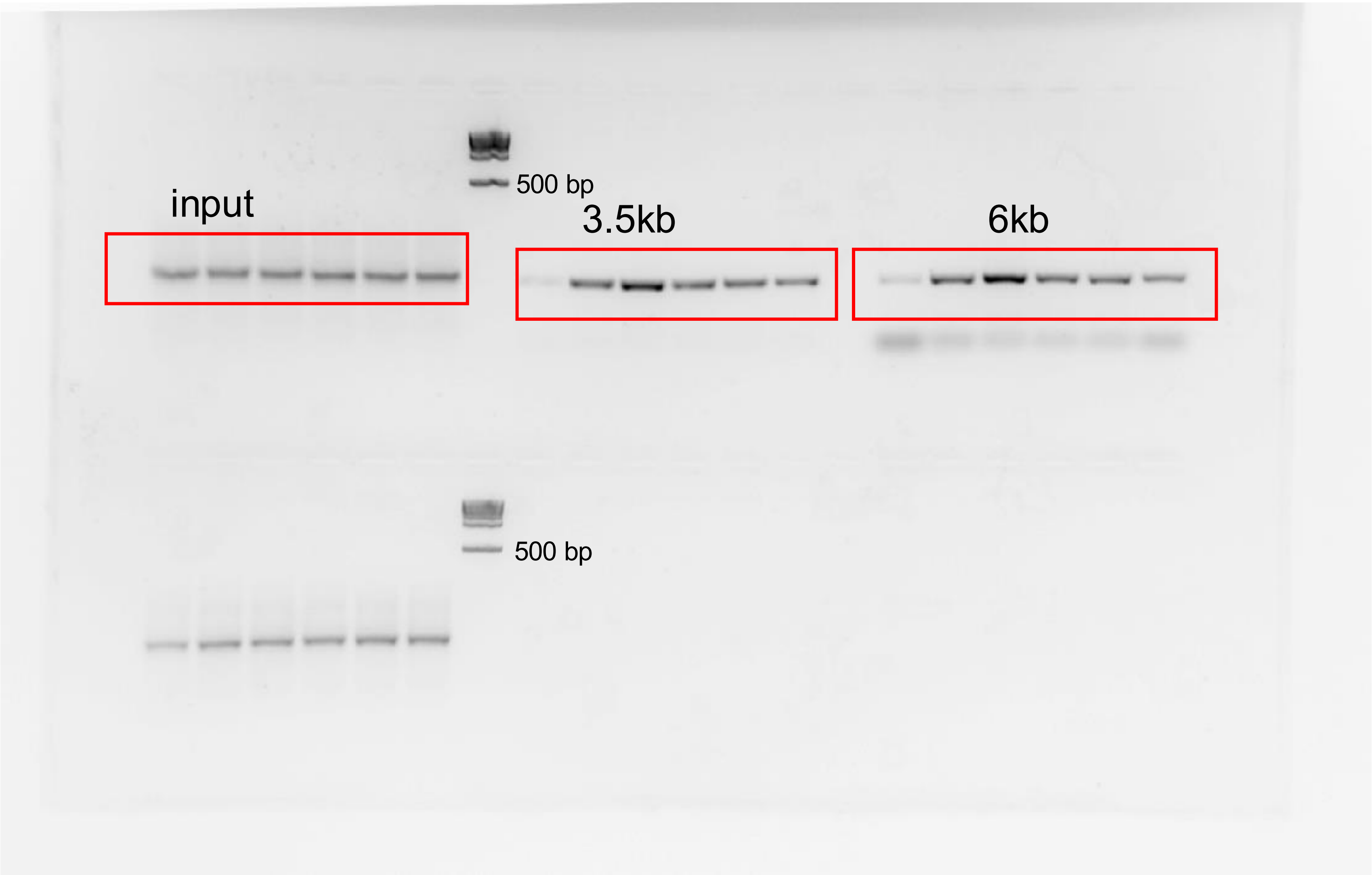

Fig. 3J

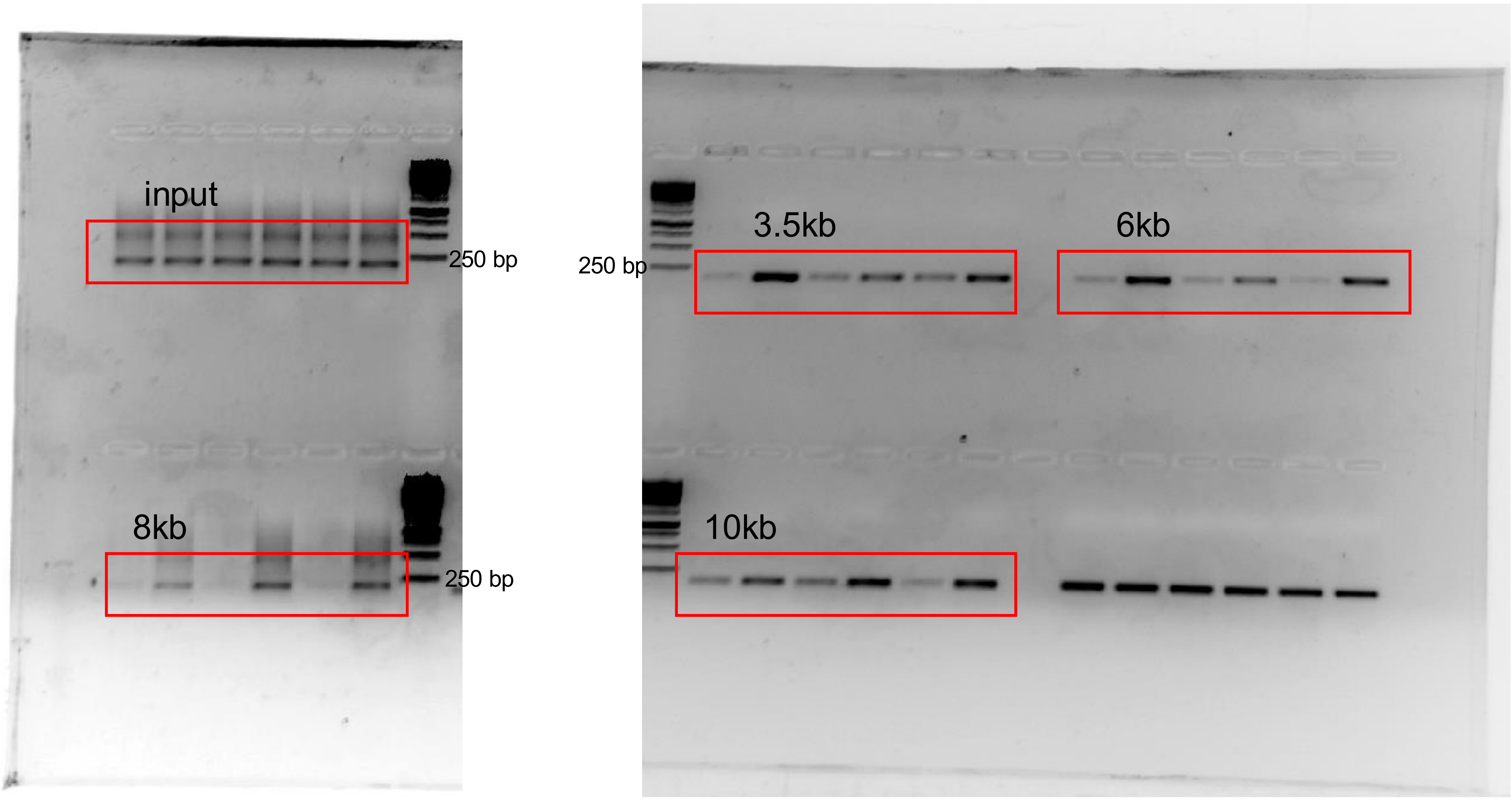

Fig. 4B

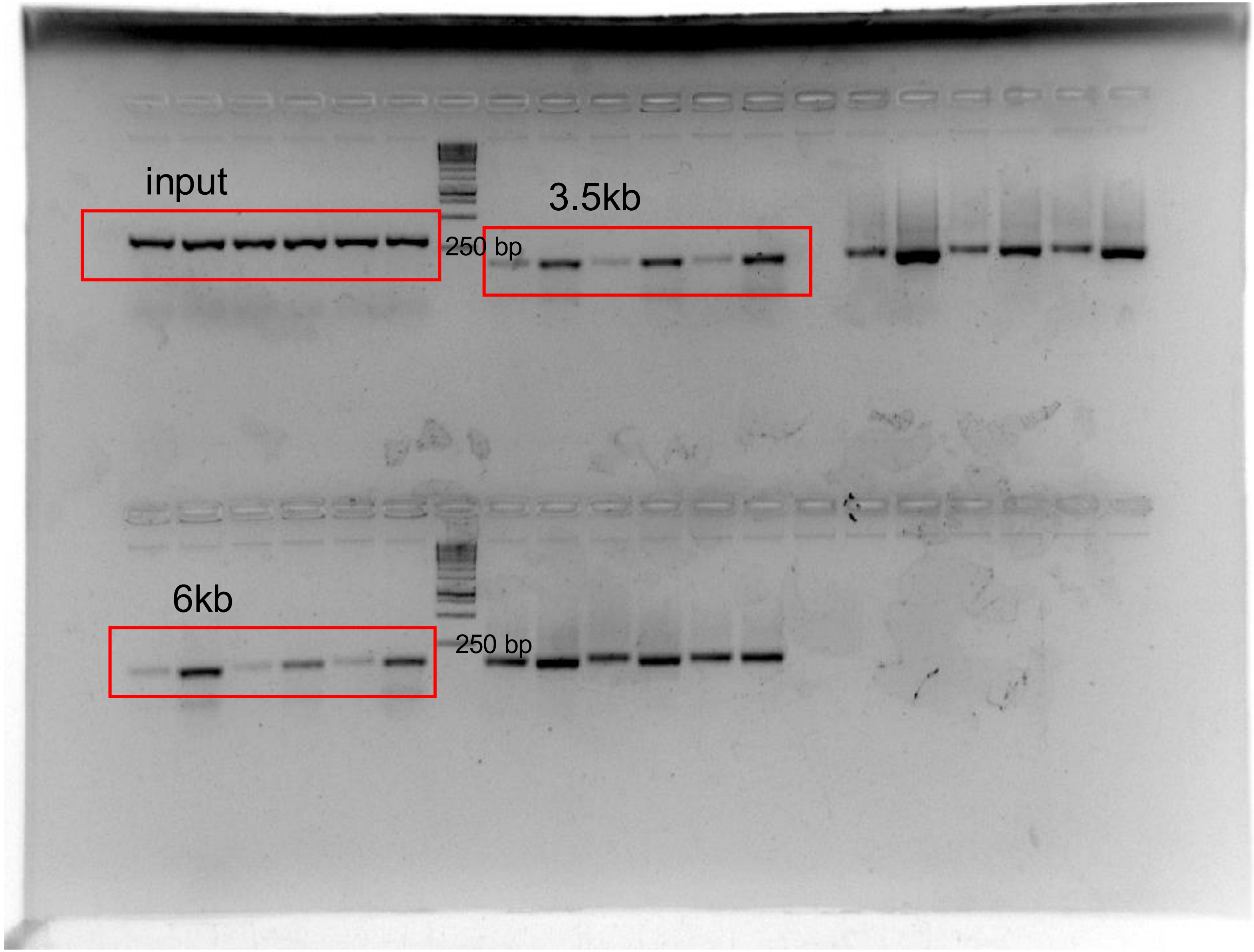

Fig. 4C

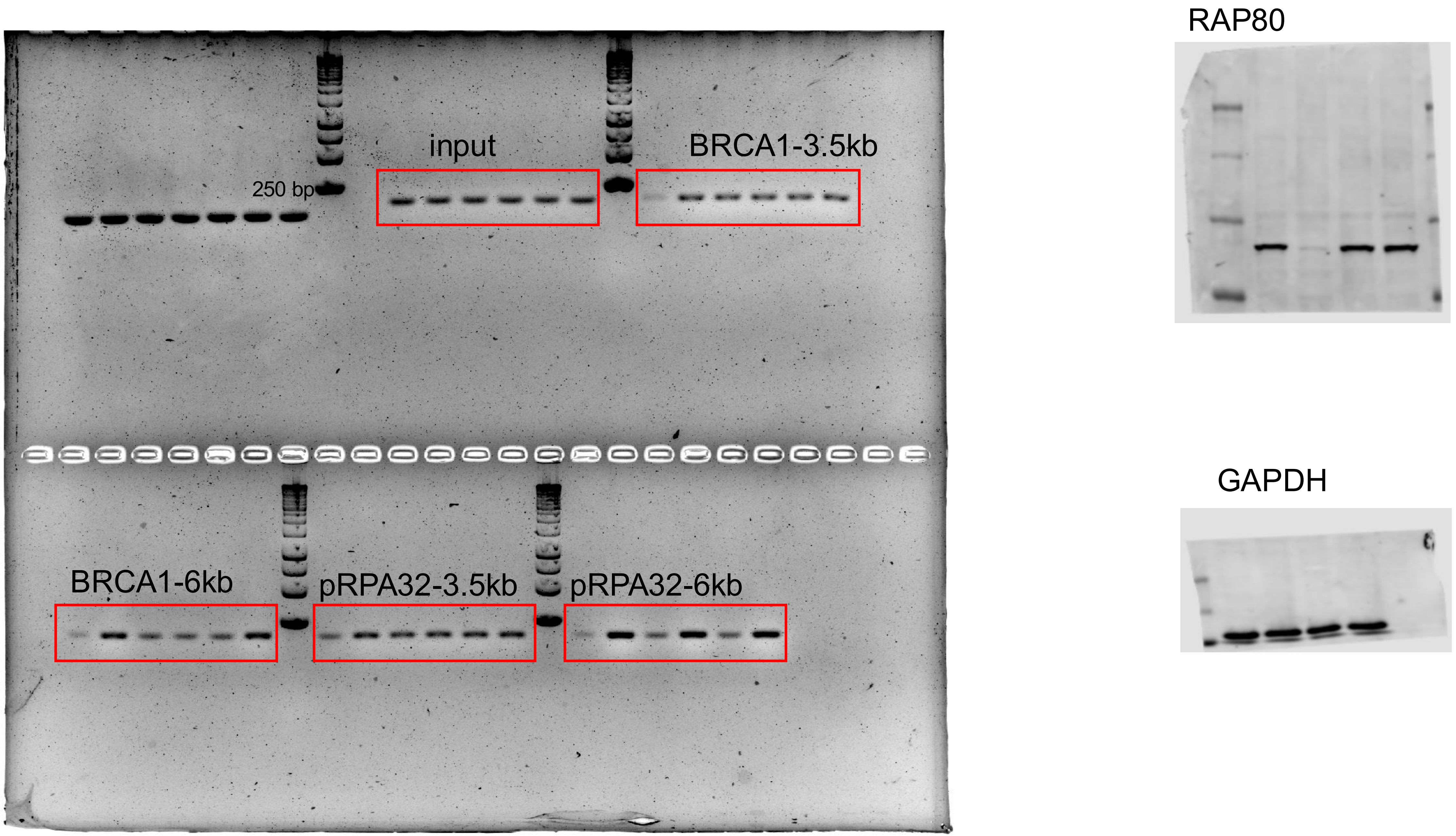
250 bp
input
BRCA1-3.5kb
BRCA1-6kb
pRPA32-3.5kb
pRPA32-6kb
RAP80
GAPDH

Fig. 5B

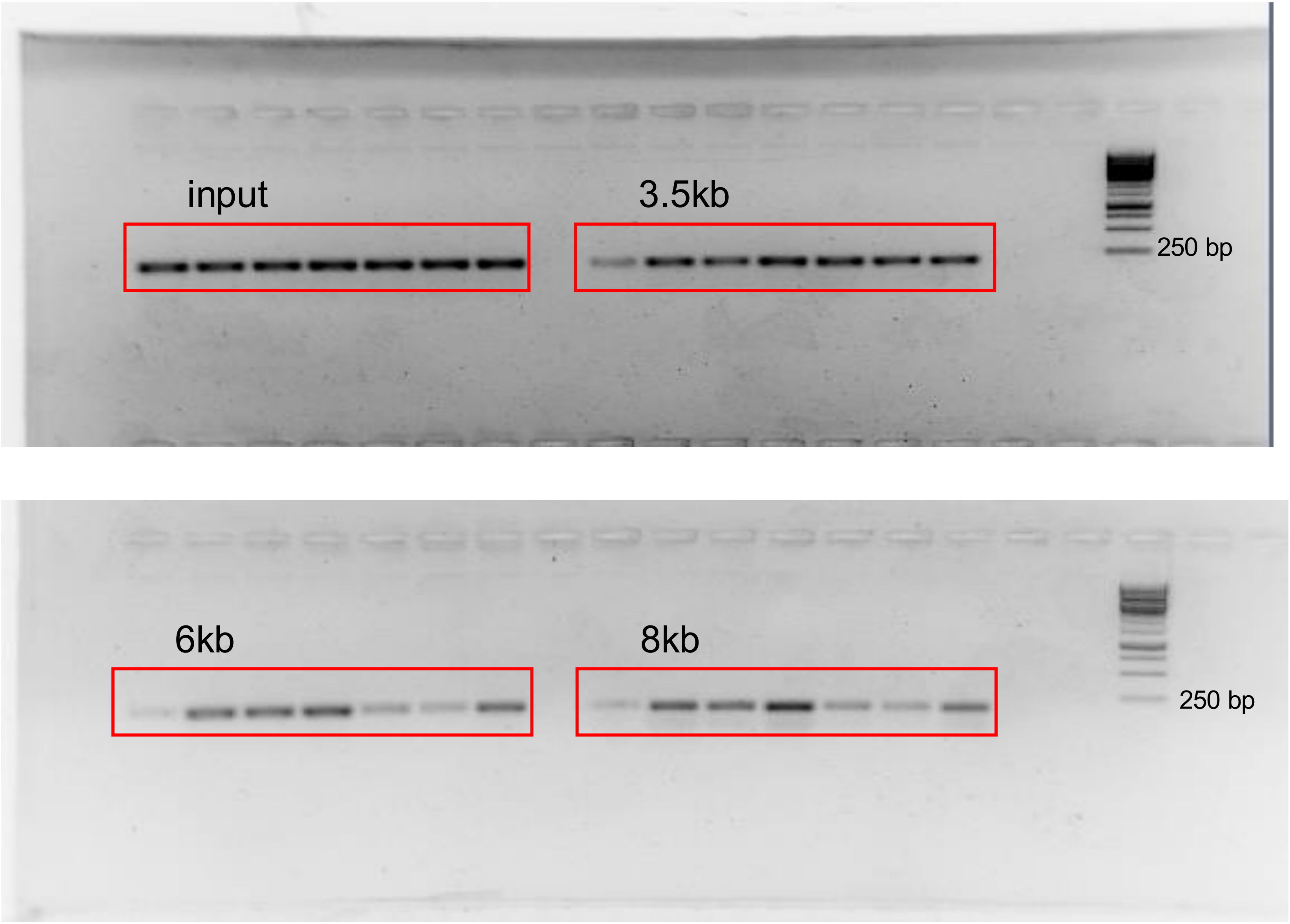

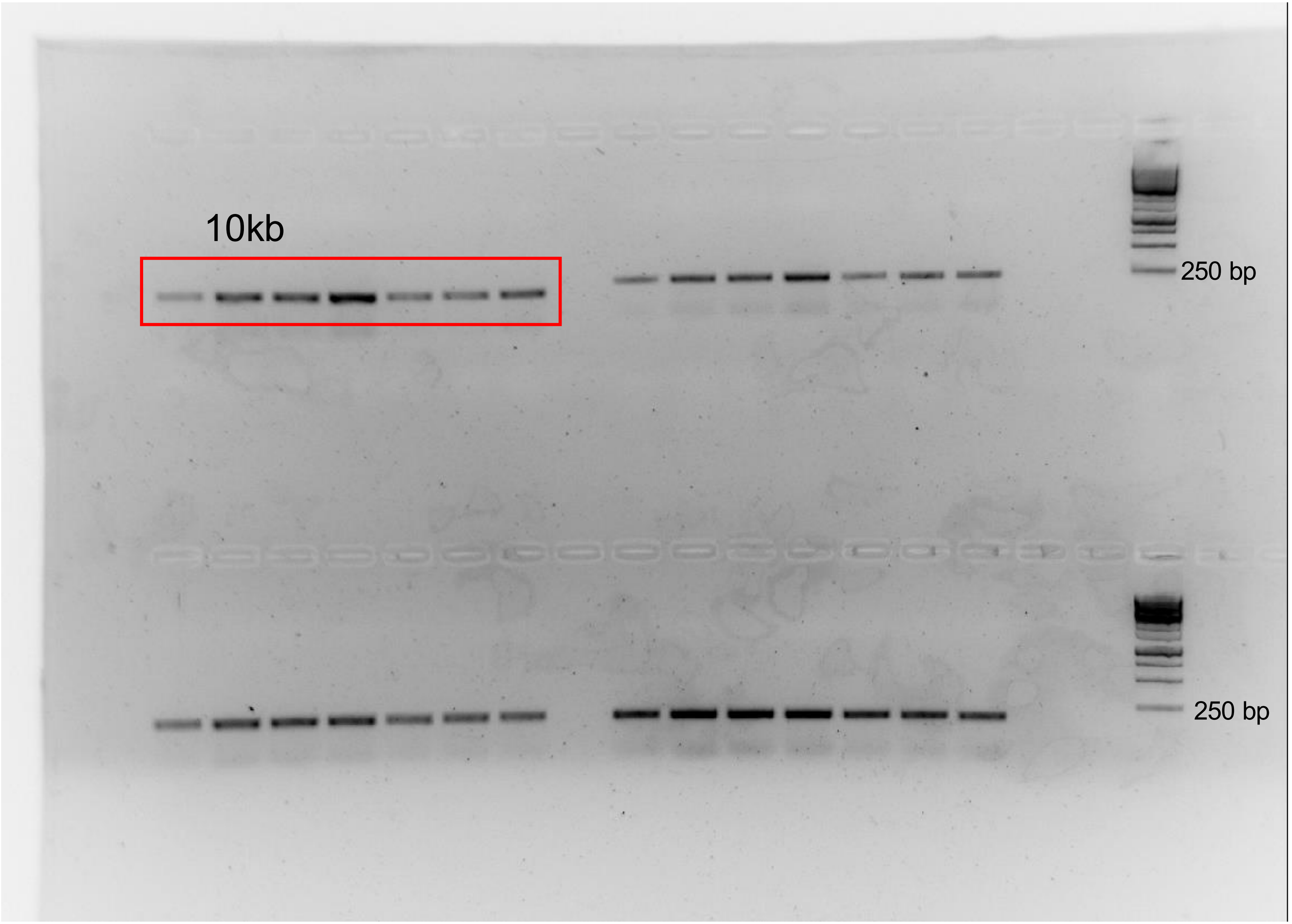
10kb
250 bp
250 bp

Fig. 6A

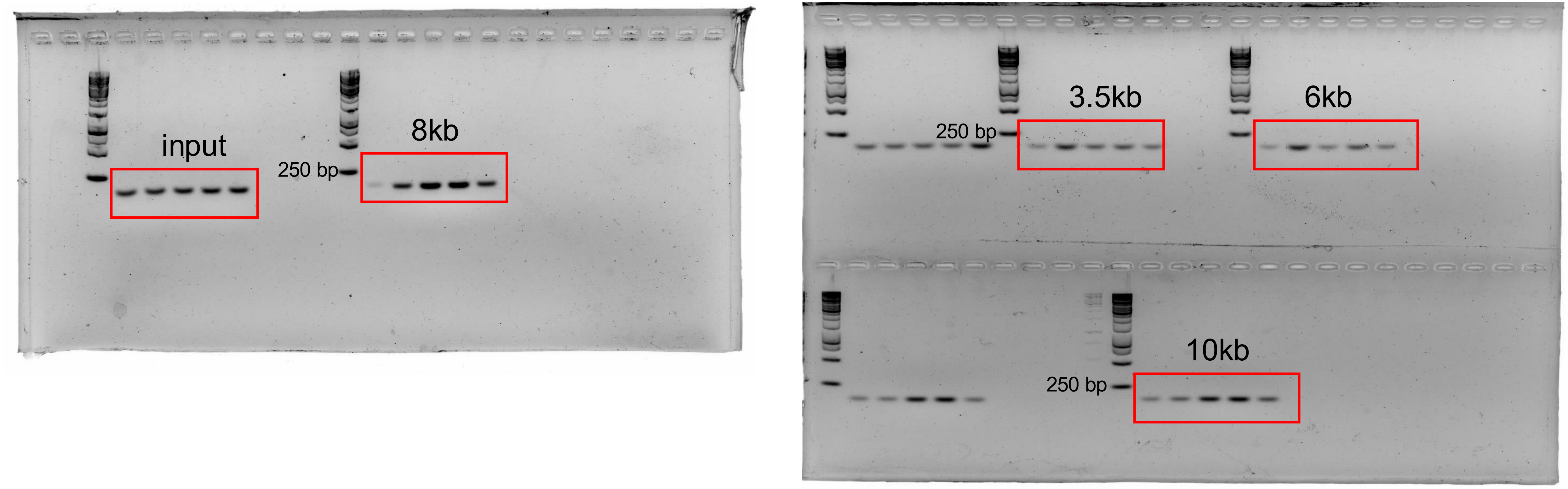

Fig. 6D

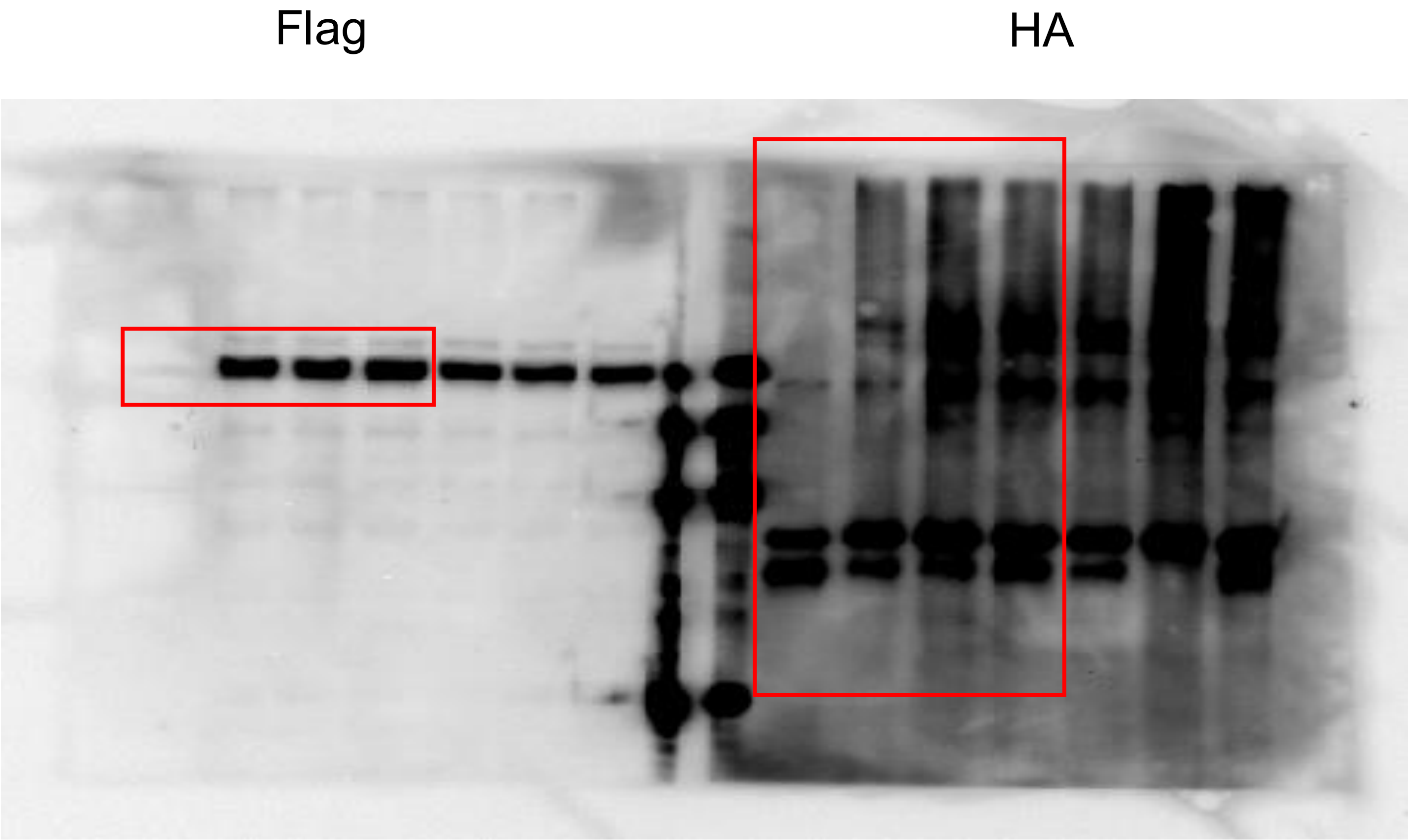

Fig. 6E

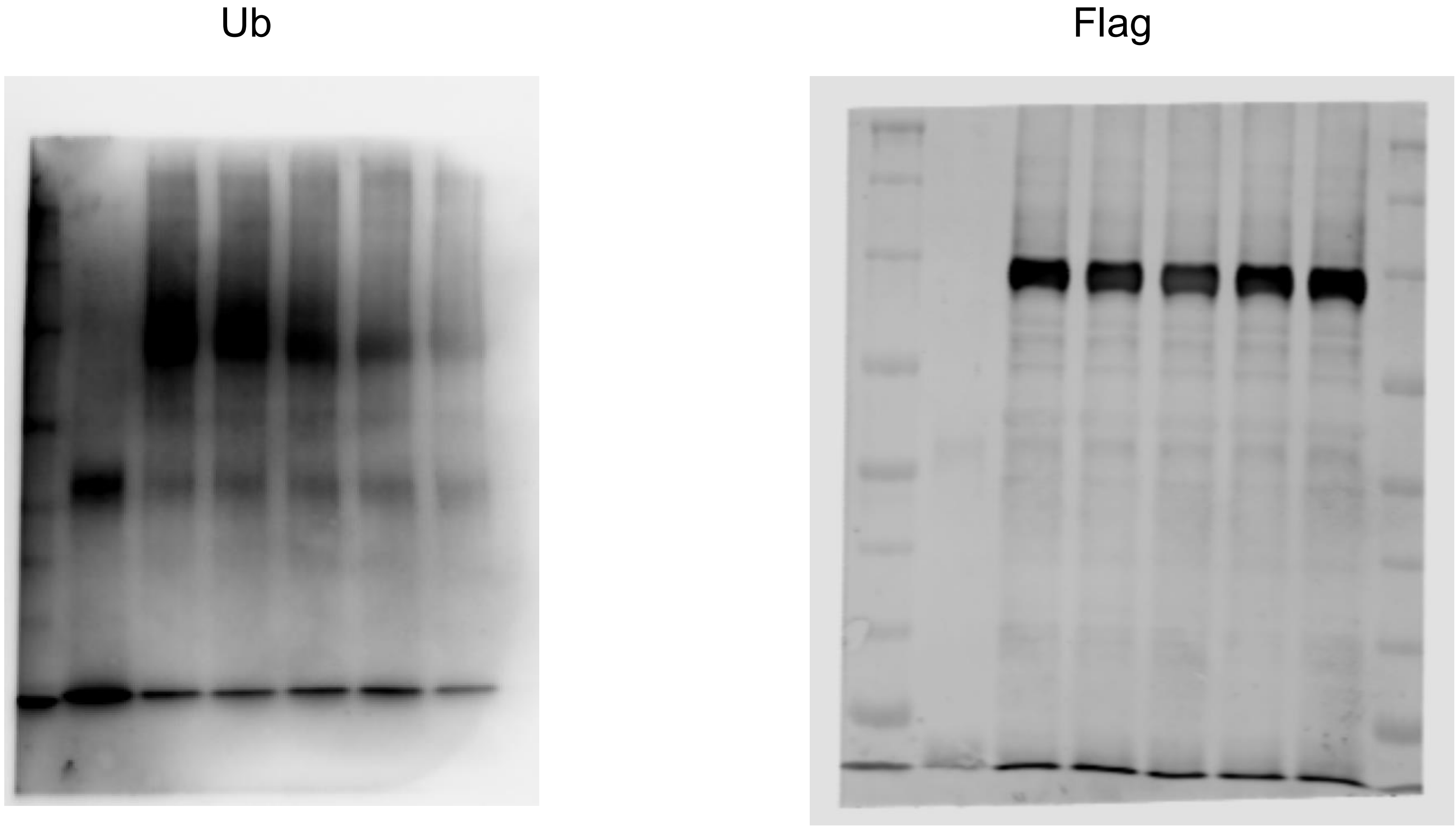

Fig. 6F

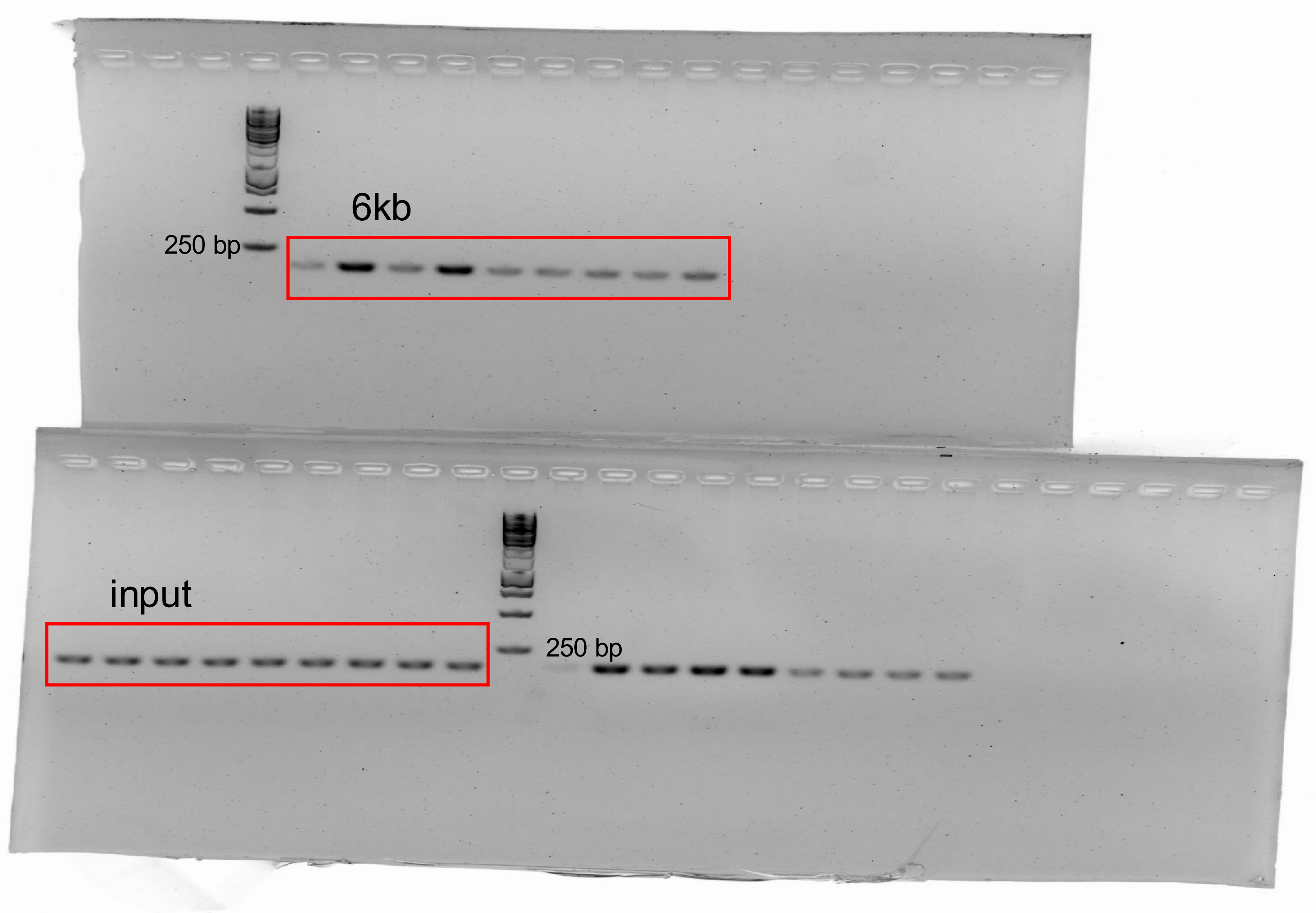

Fig. 6G

pRPA32

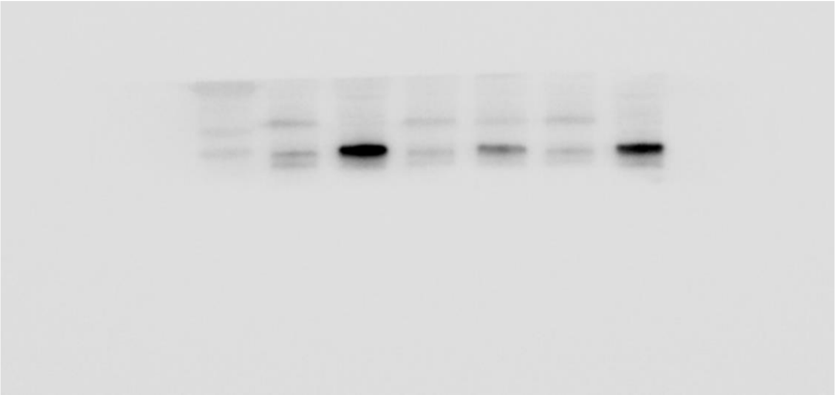

H3

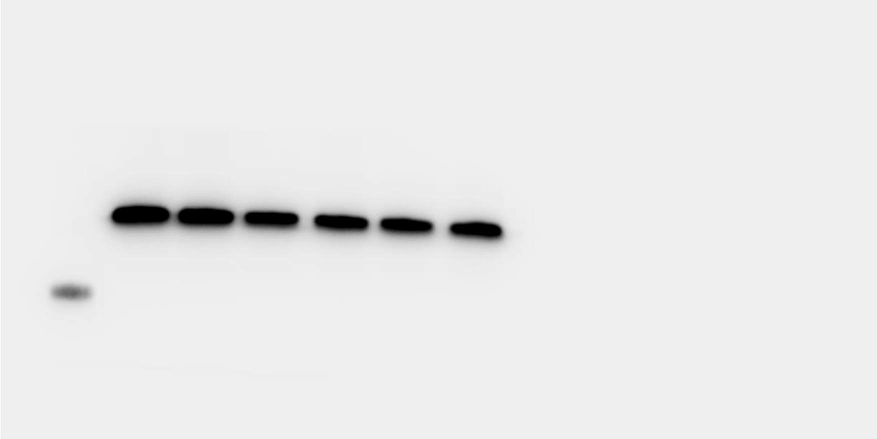

Fig. 6H

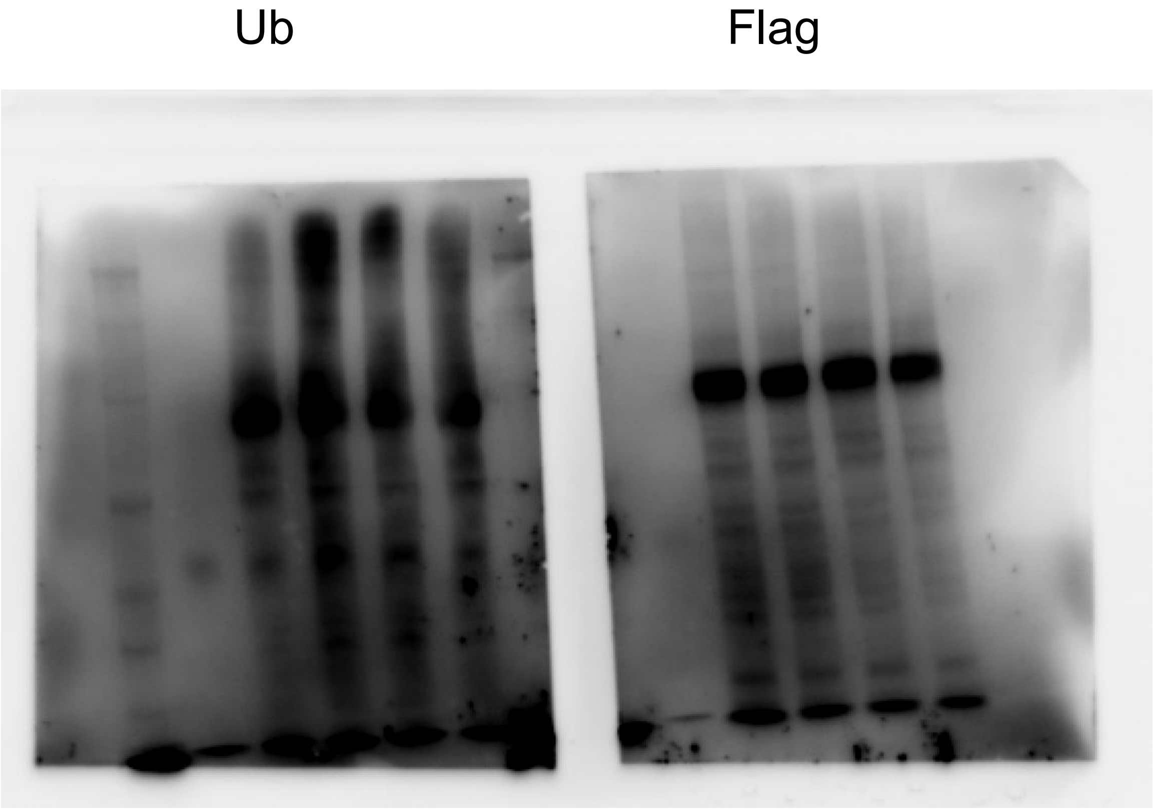

Fig. S1E

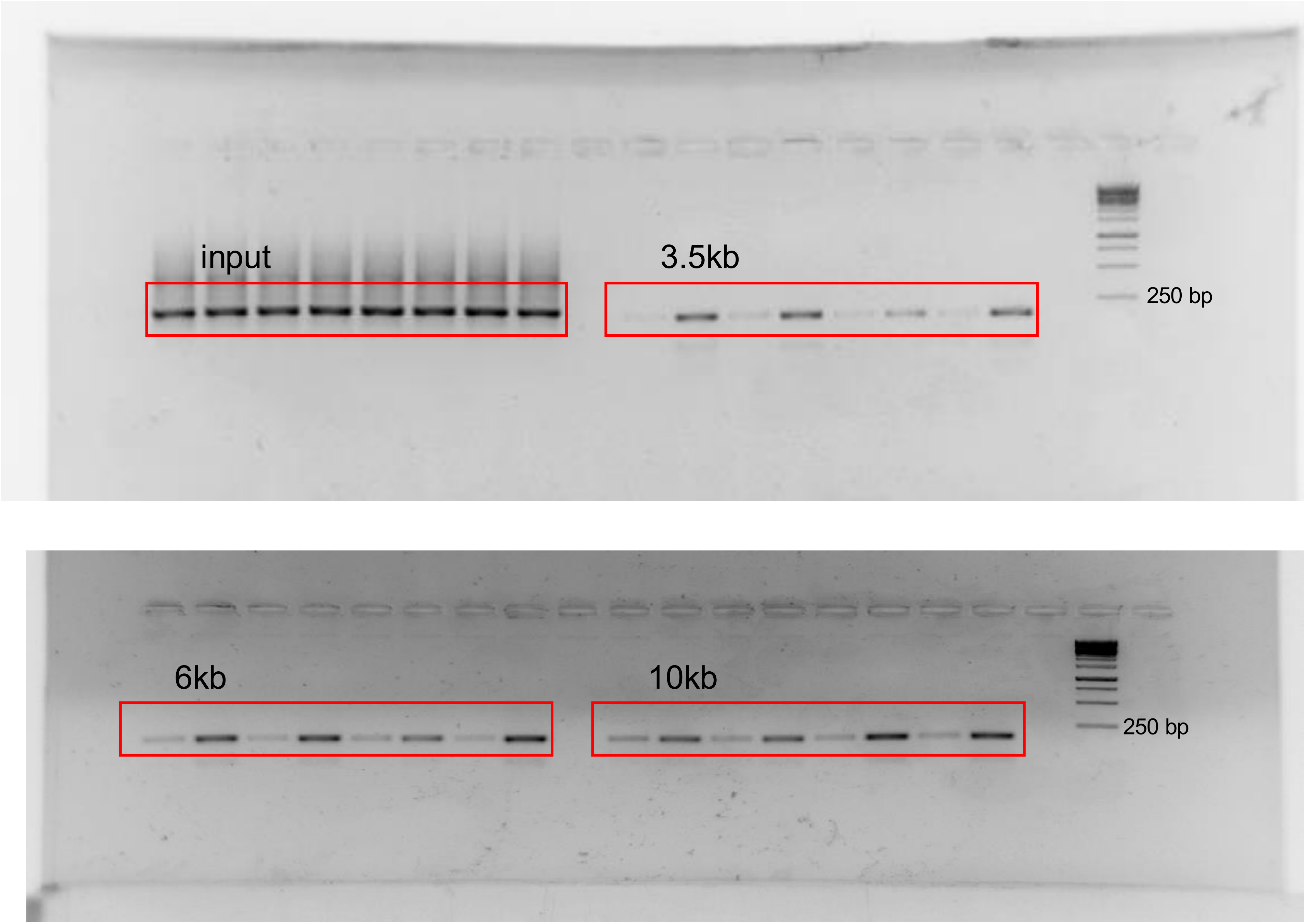

Fig. S1F

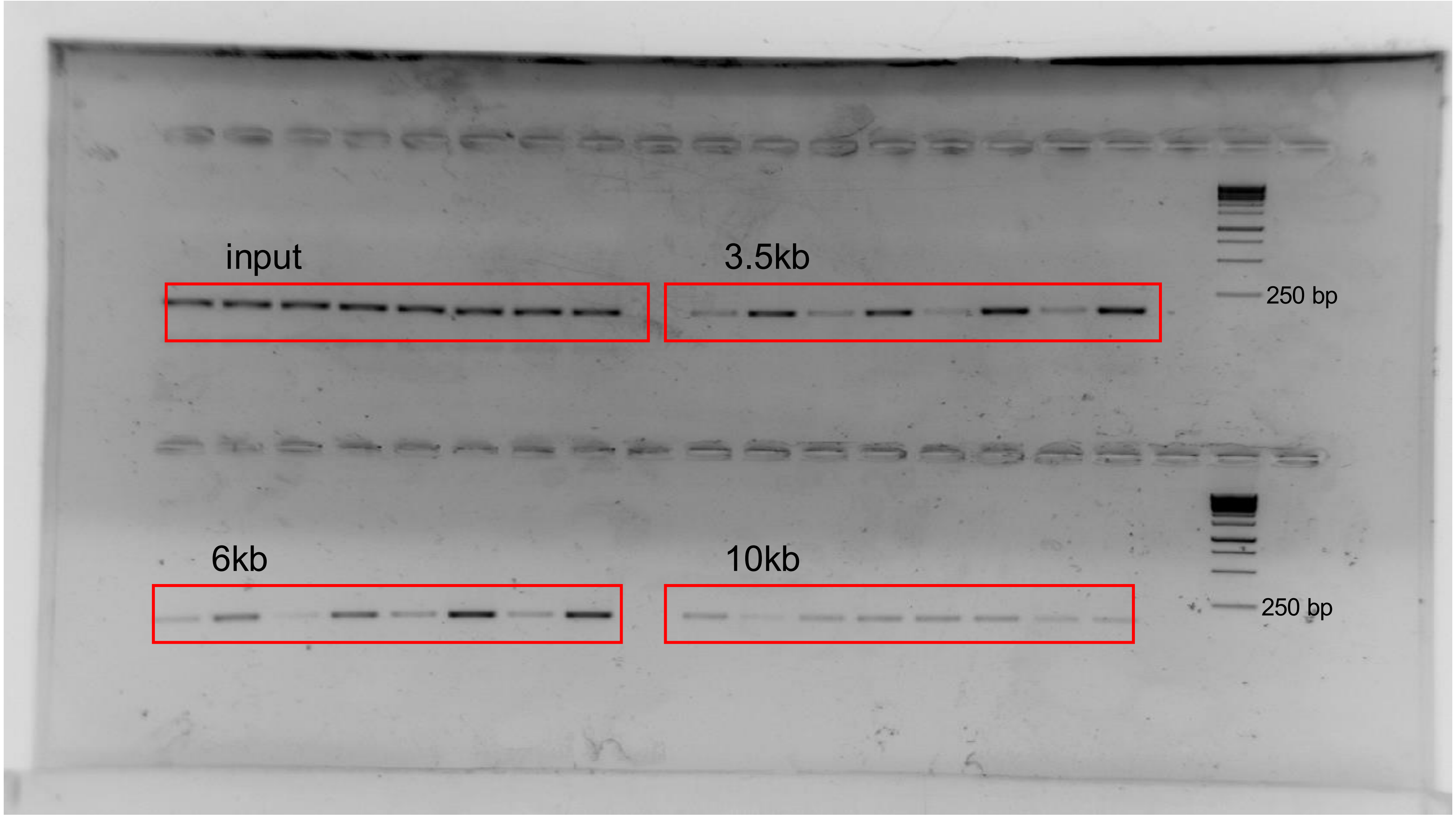

Fig. S2C

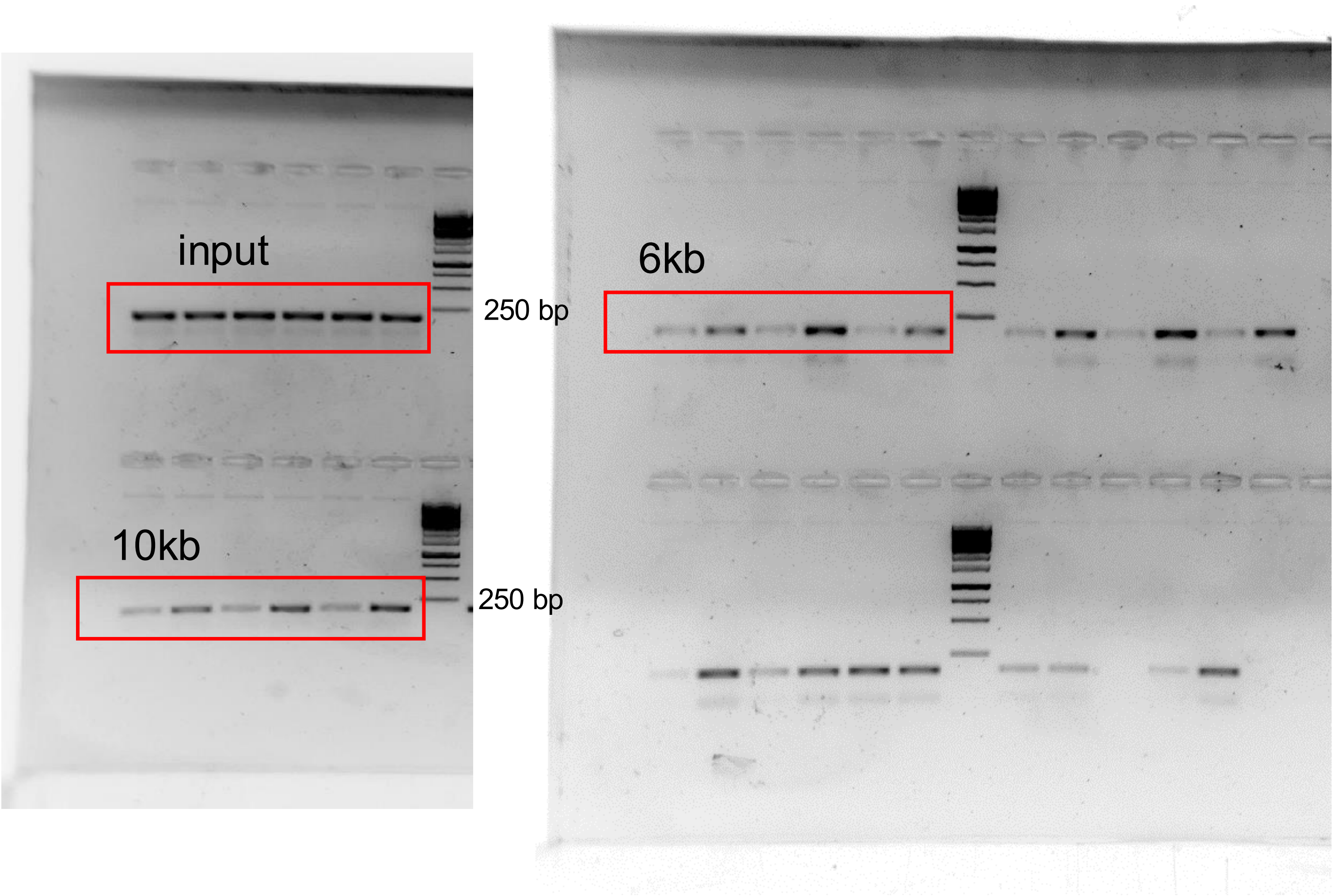

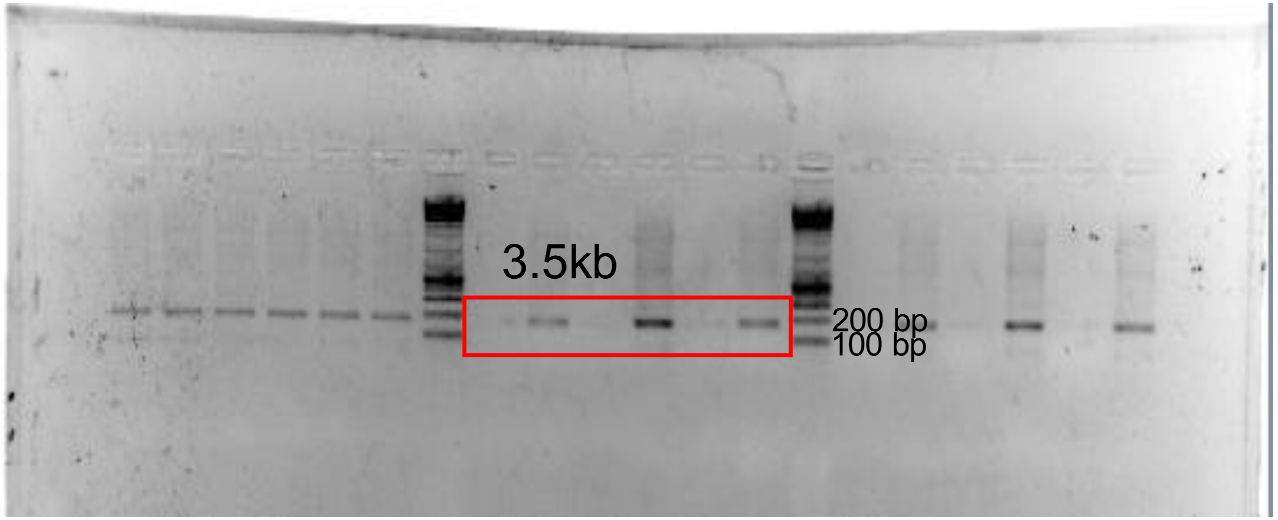

3.5kb
200 bp
100 bp

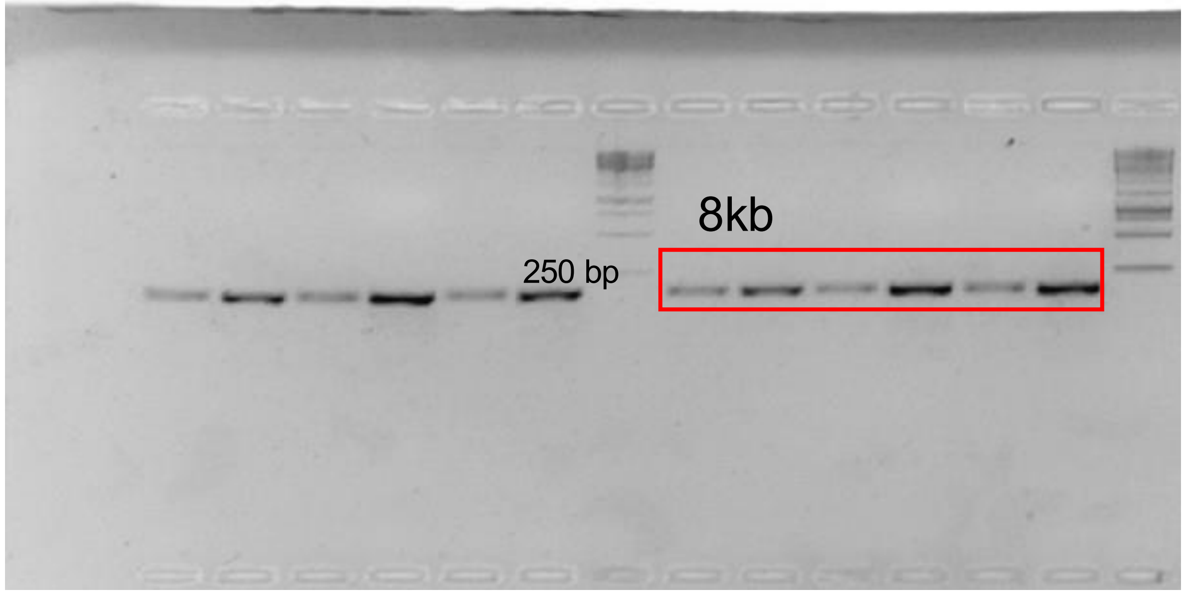

8kb
250 bp

Fig. S2D

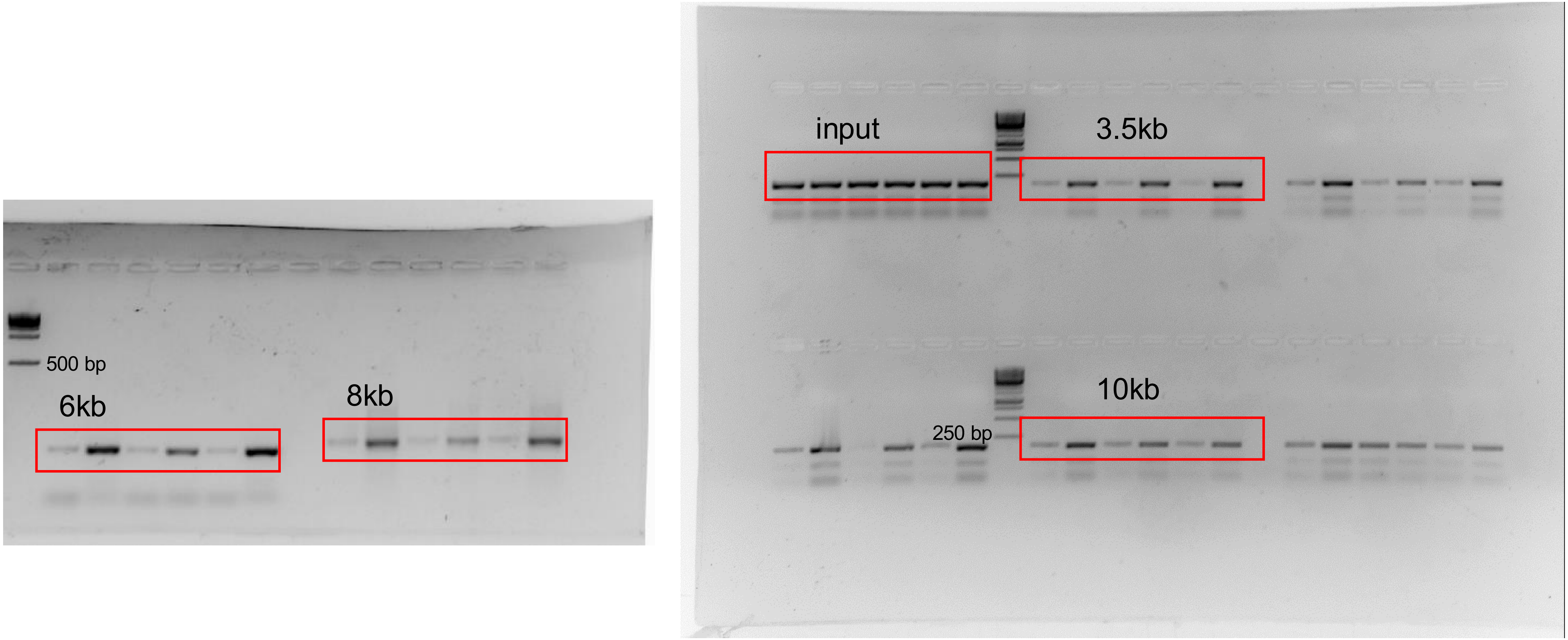

Fig. S3A

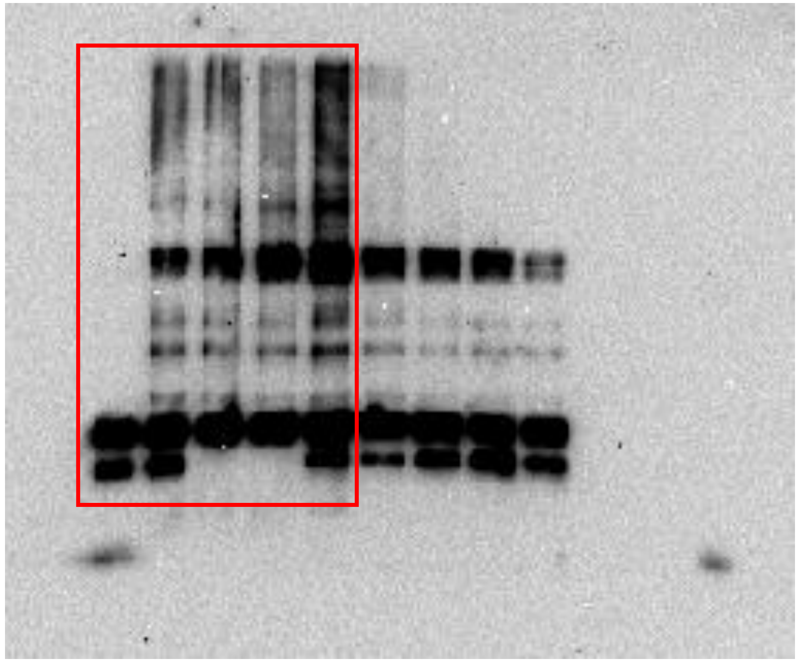

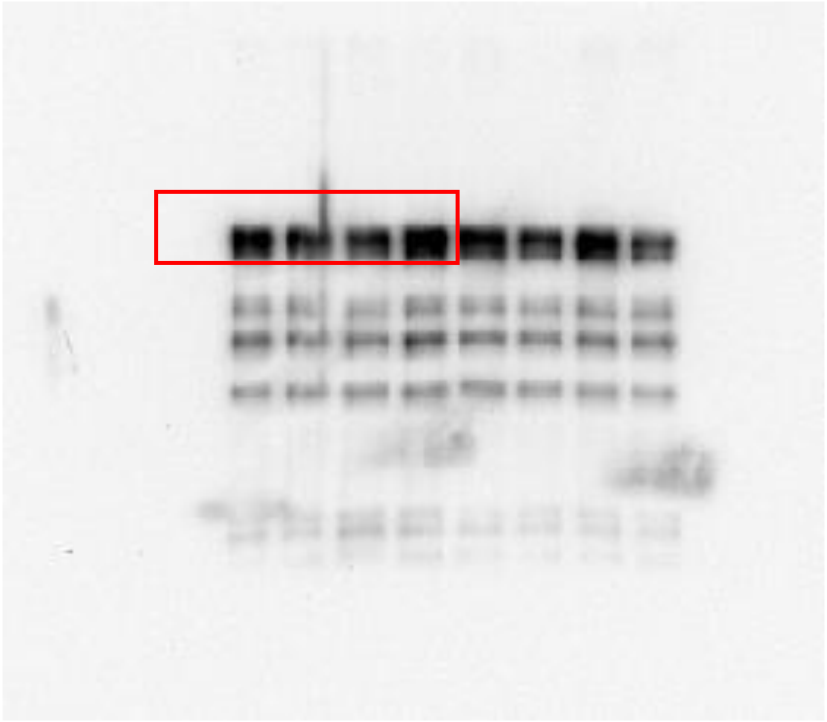

Fig. S3B

Ub

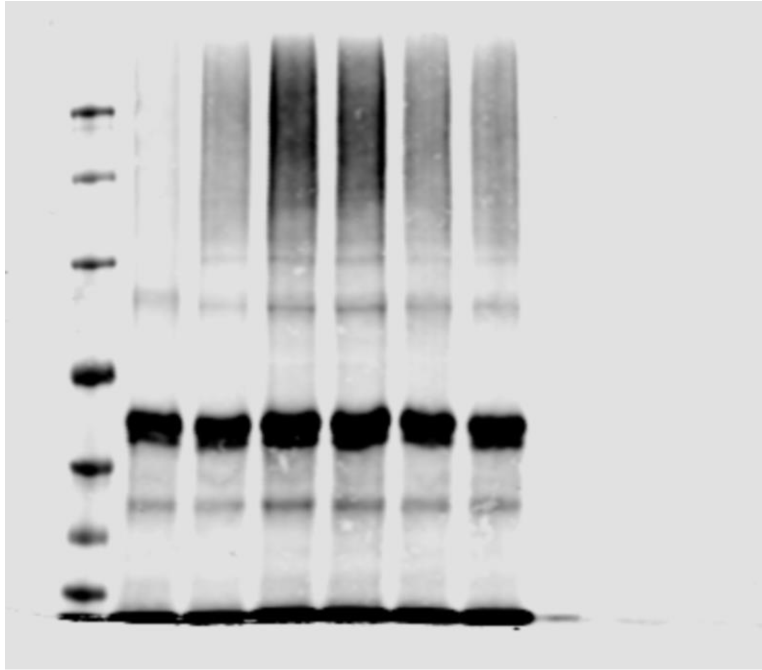

Flag

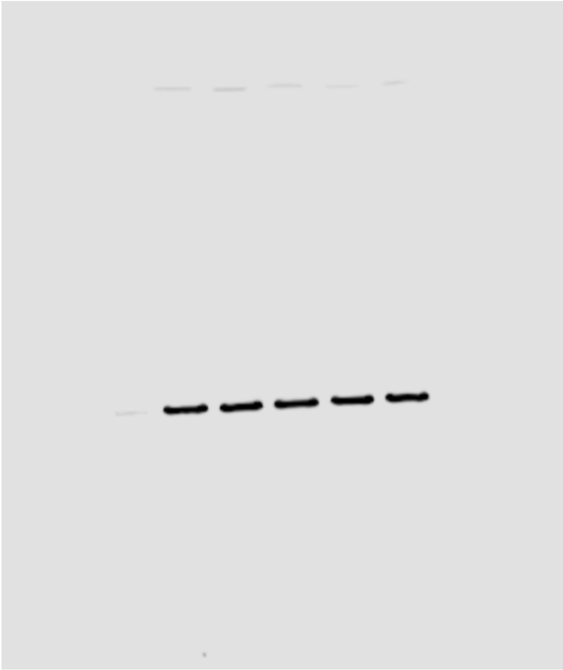

Fig. S3C

V5

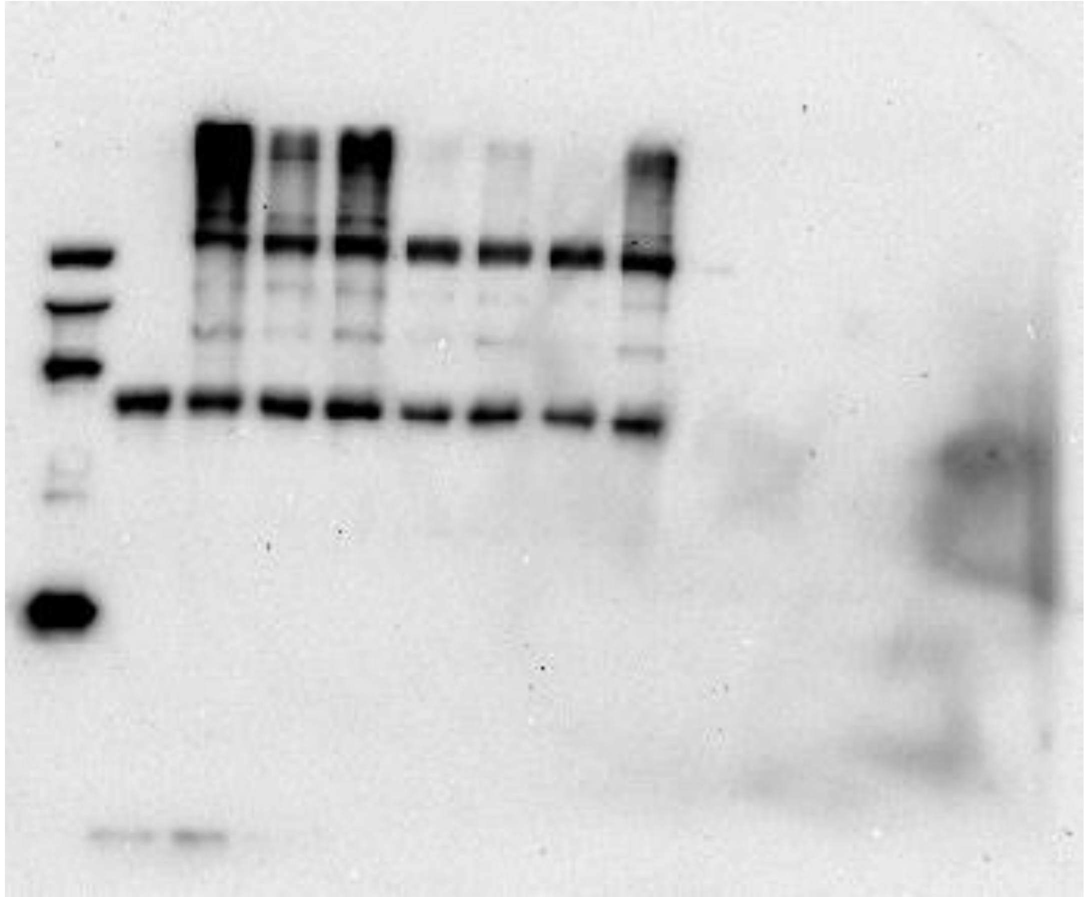

Flag

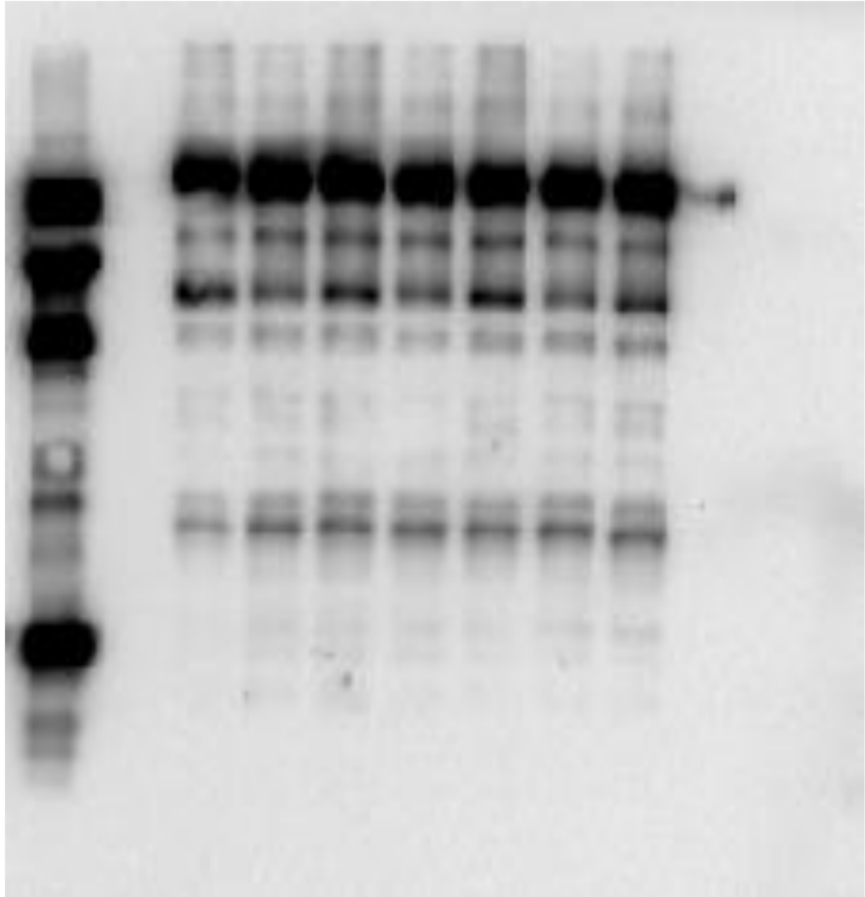

Fig. S3F

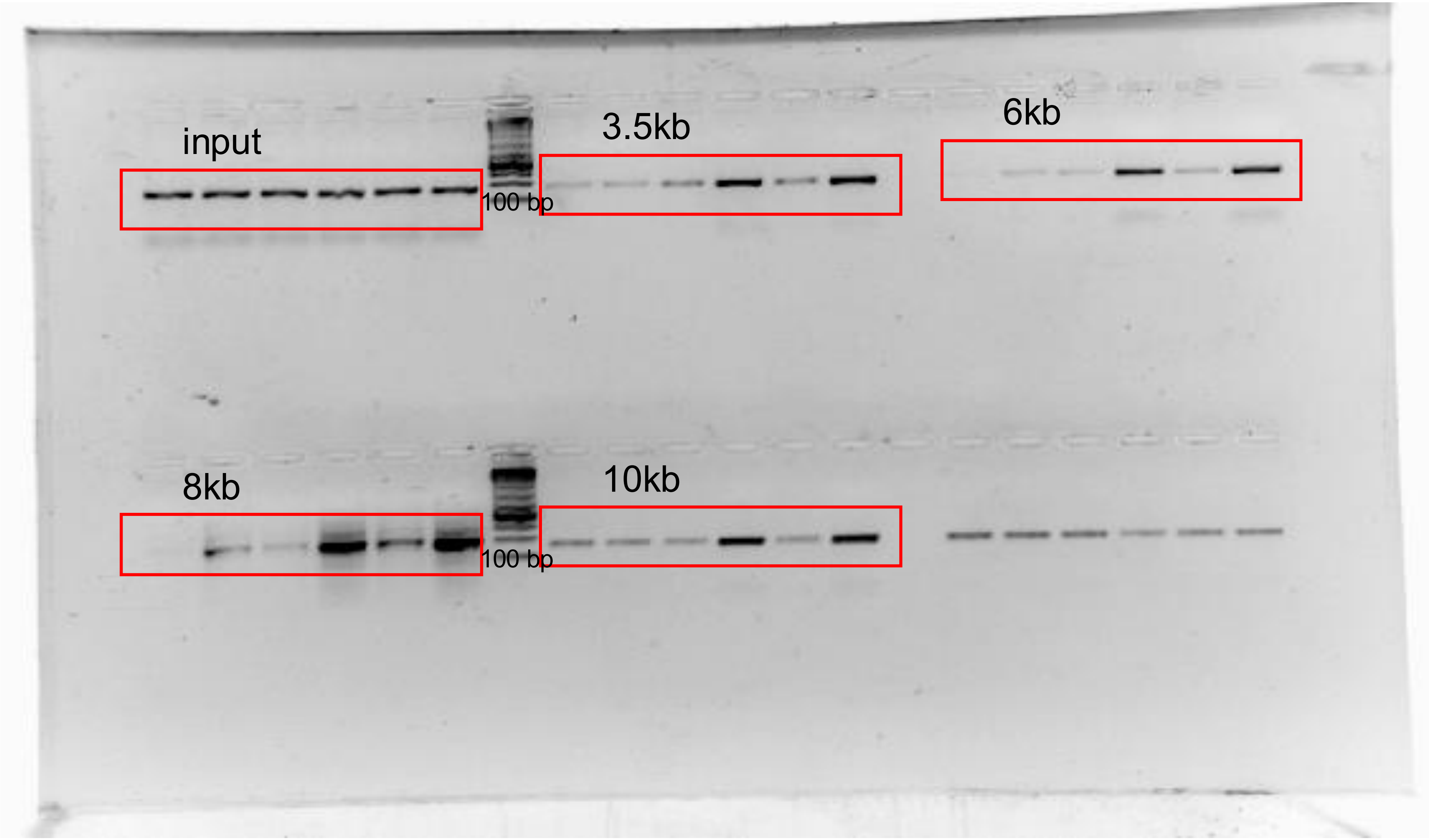

Fig. S3H

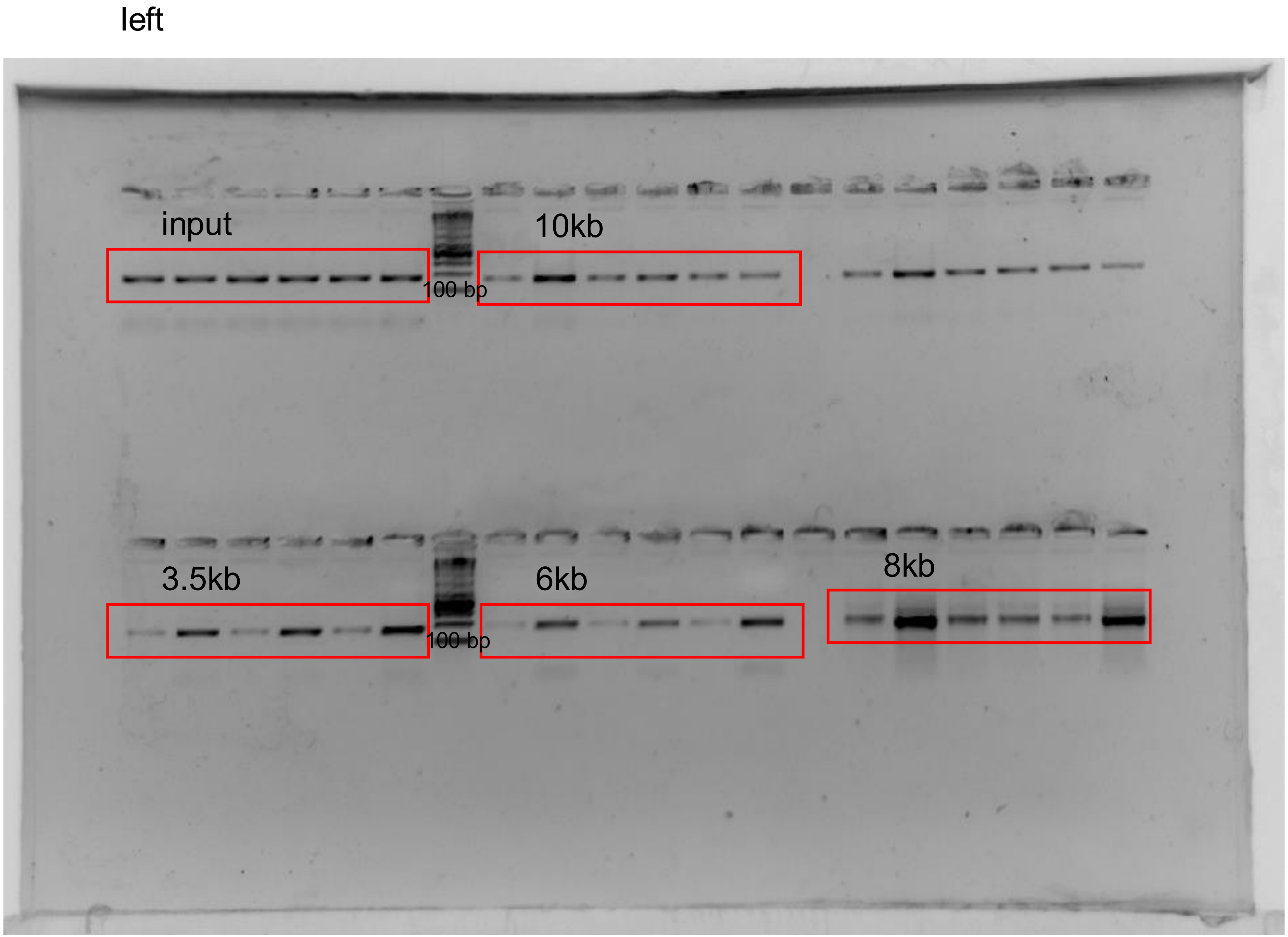

right

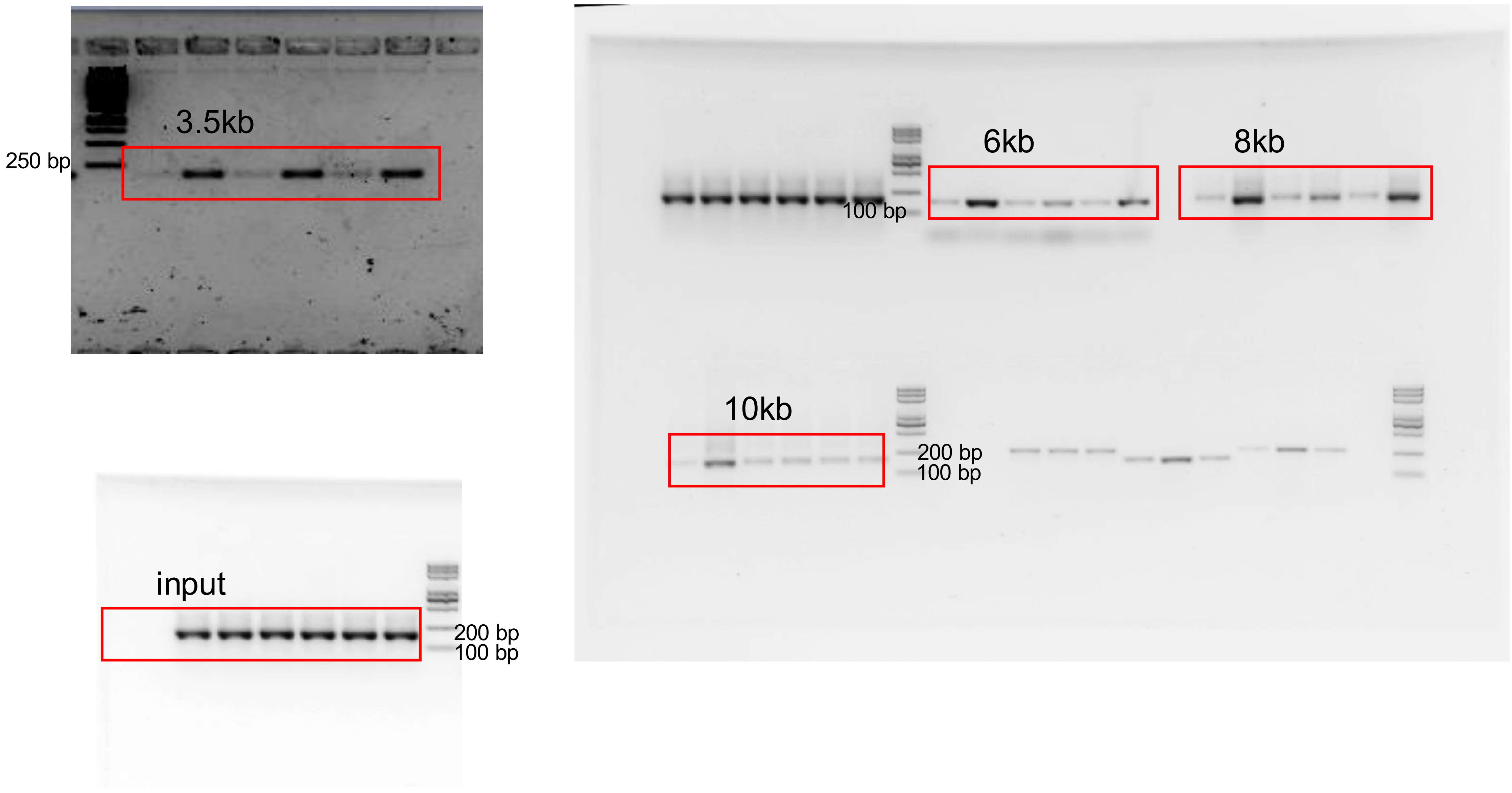

Fig. S3G

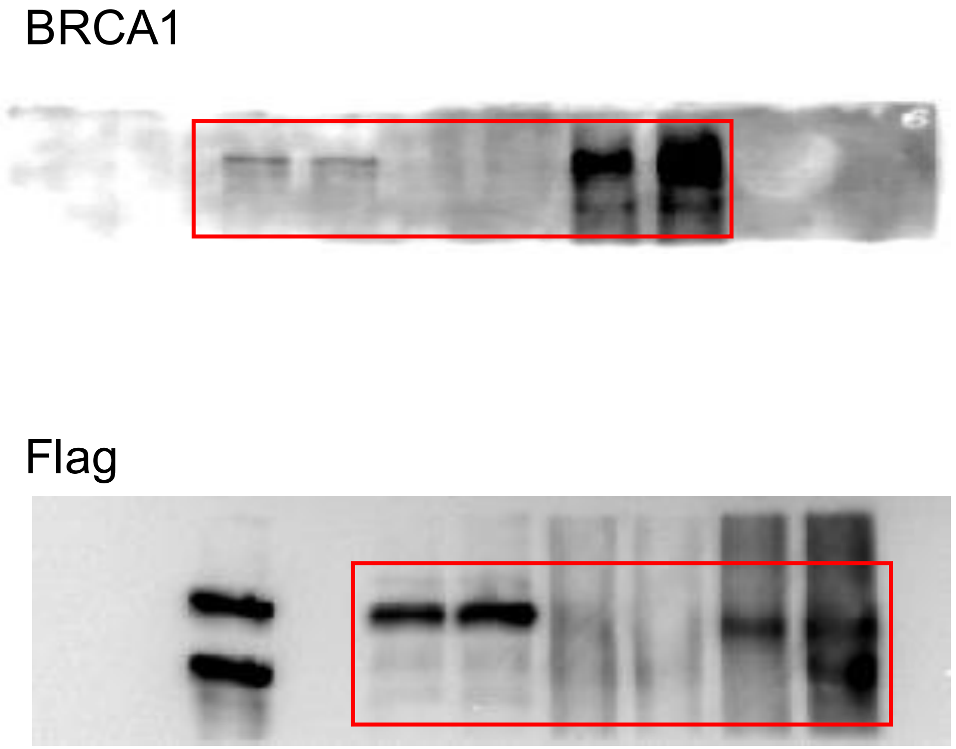

Fig. S3J

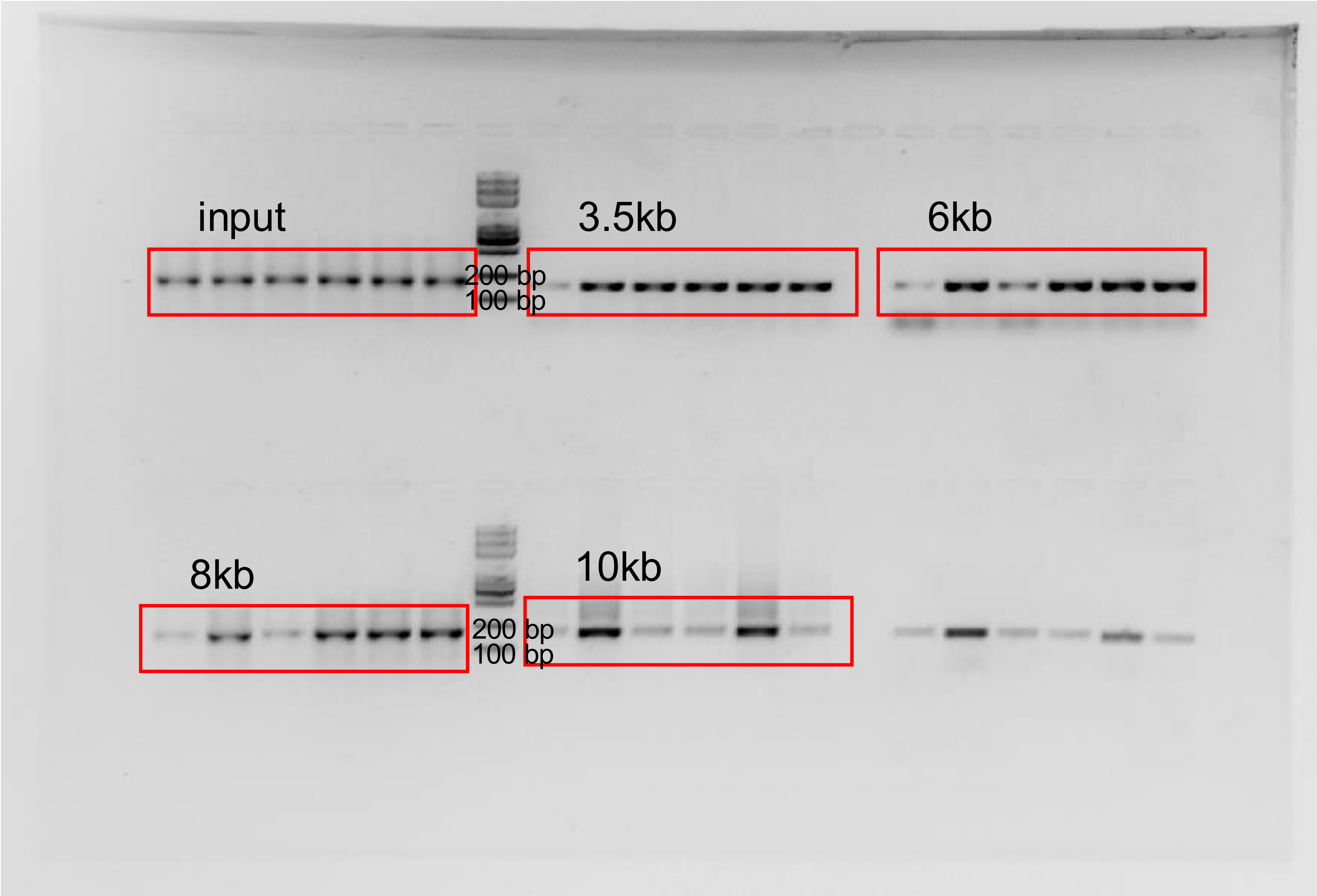

Fig. S4B left

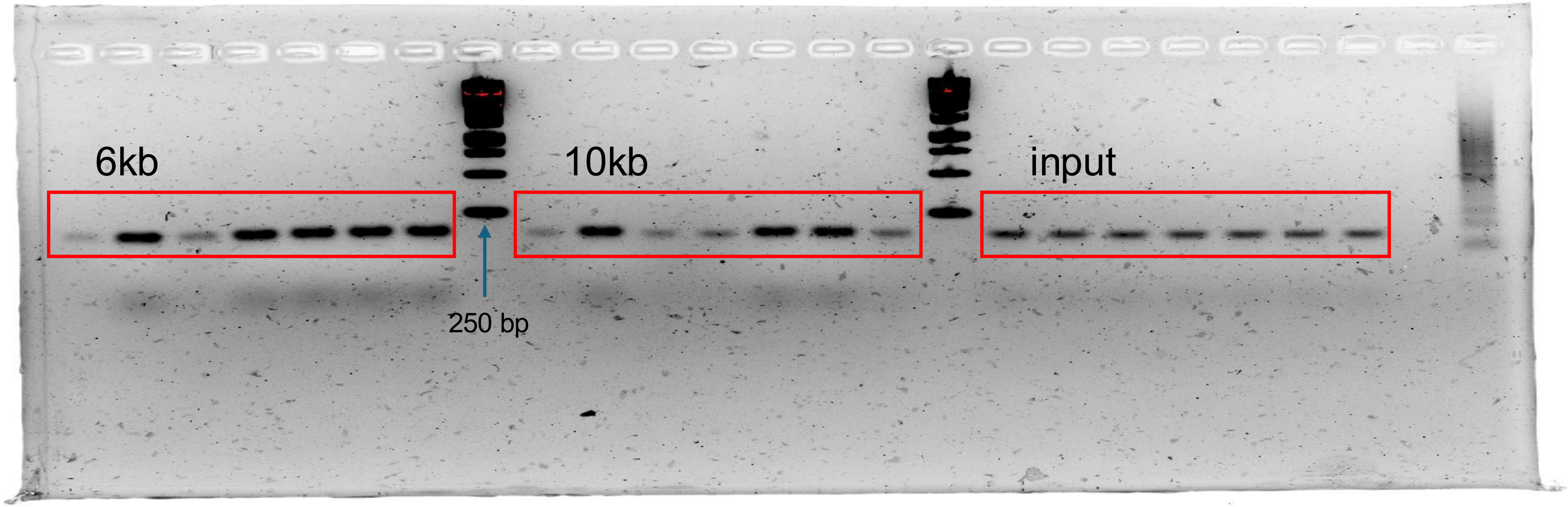

right

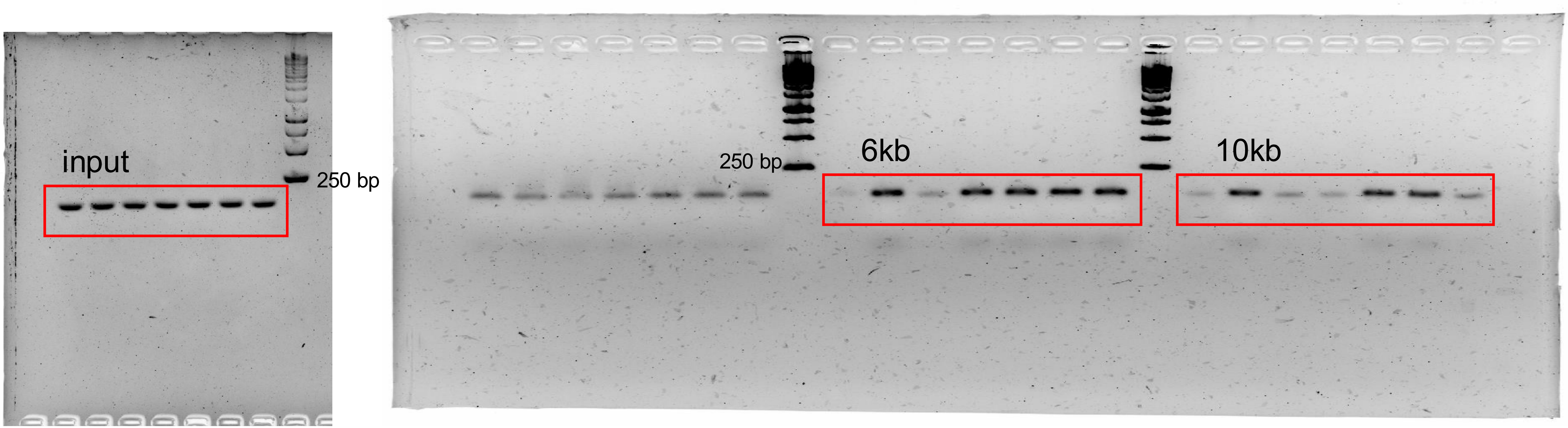

Fig. S5A

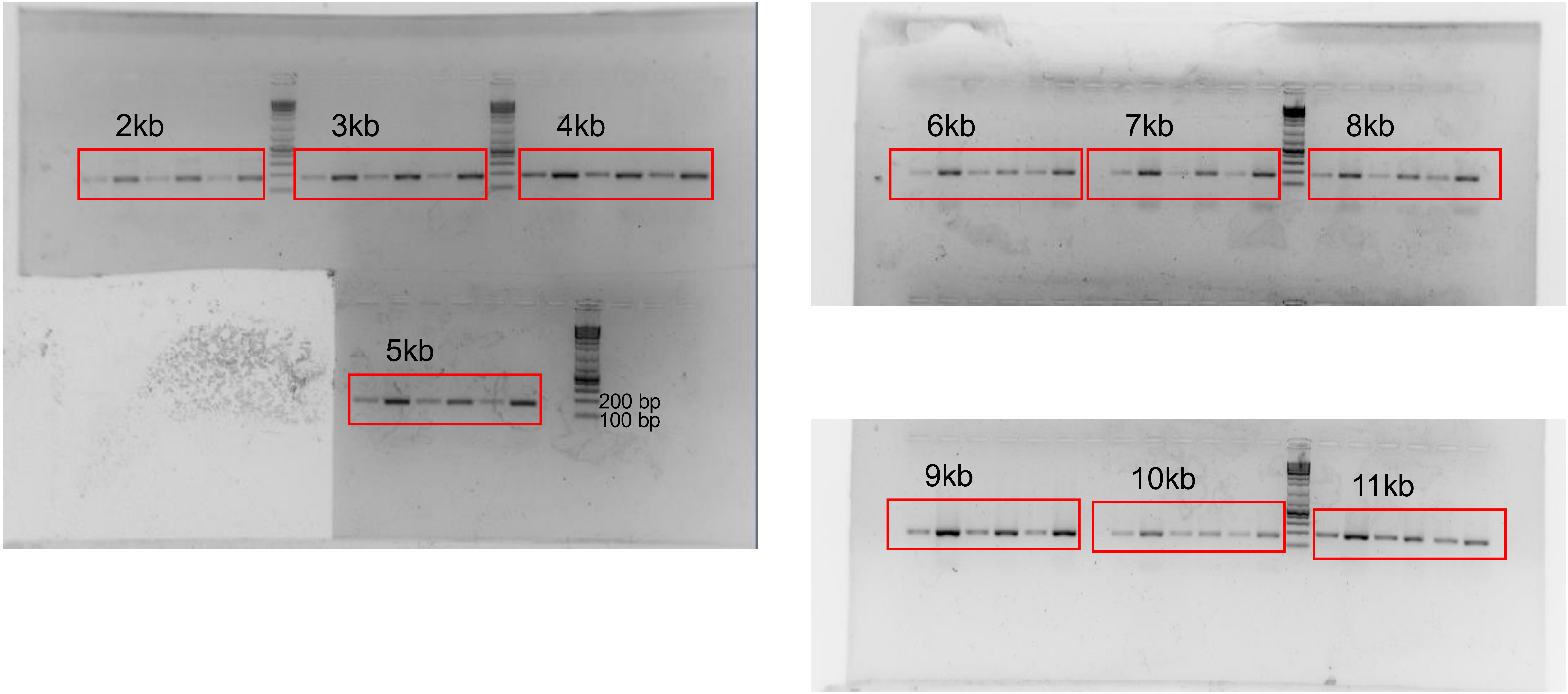

Fig. S5B

pRPA32

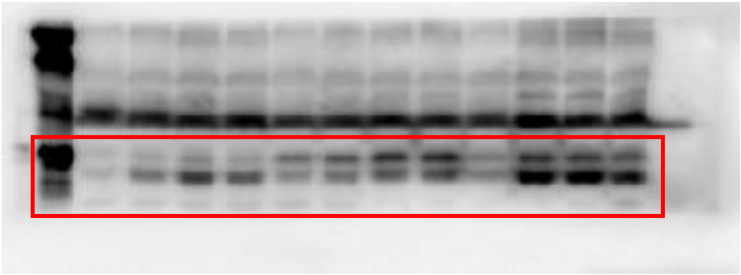

H3

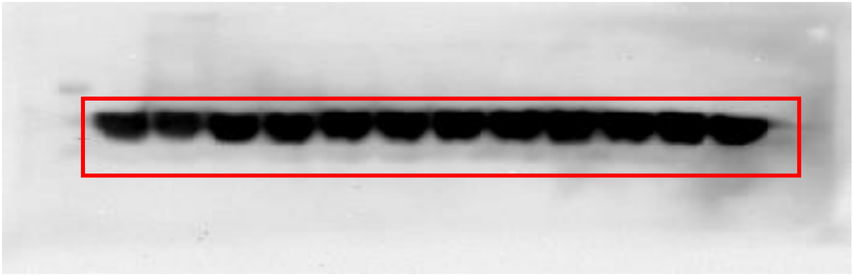

Fig. S5F

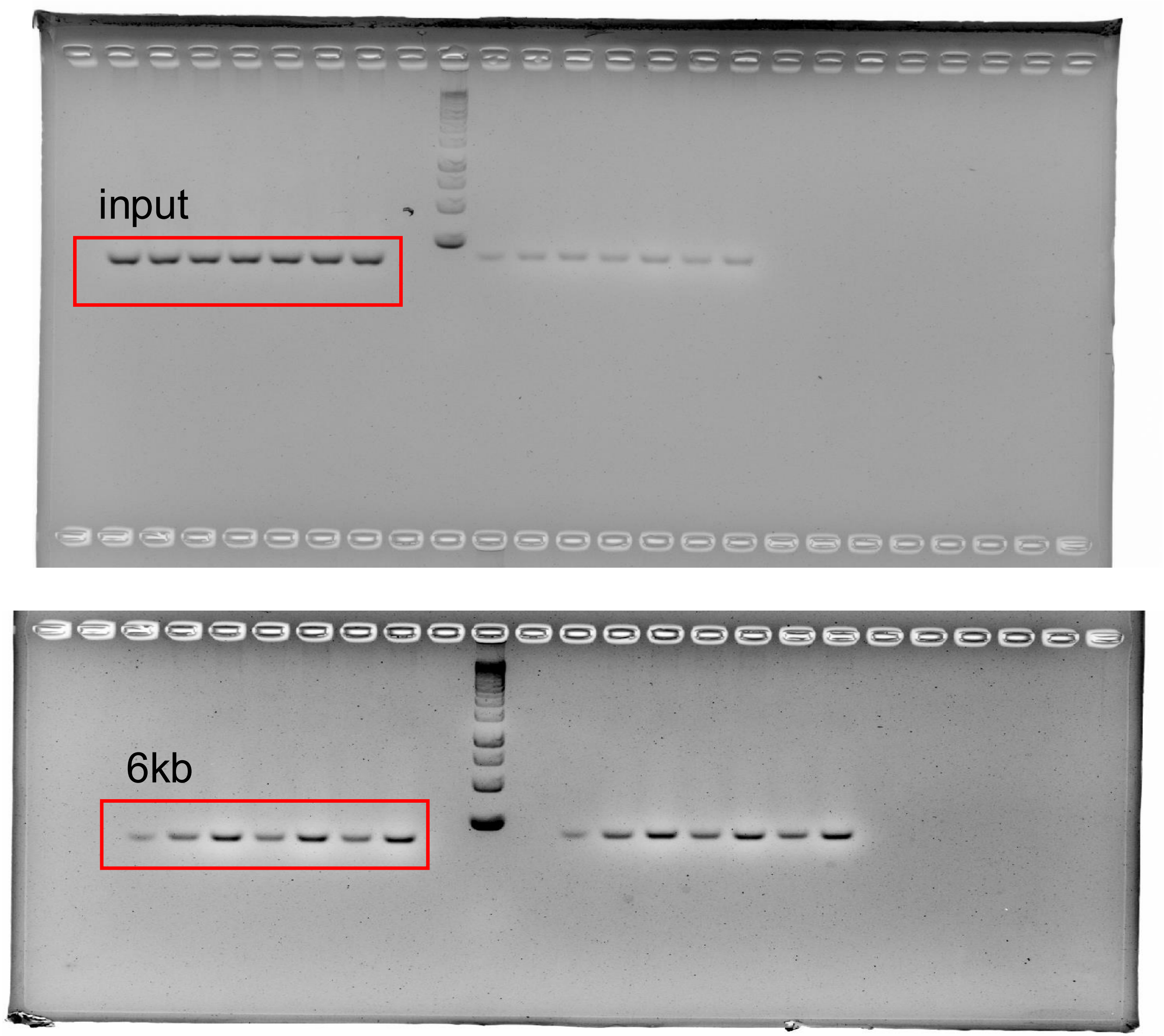

Fig. S6B

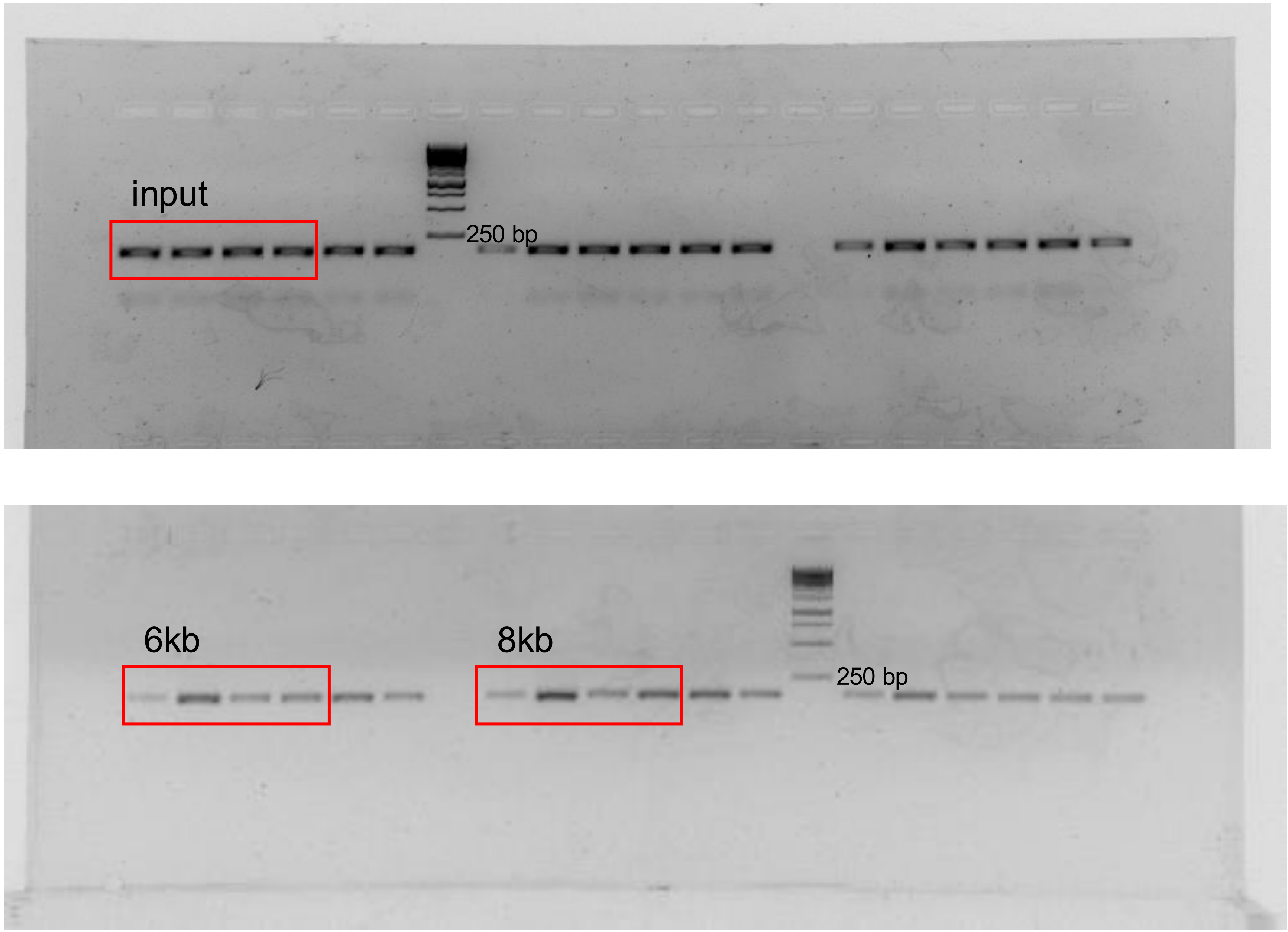

Fig. S6C

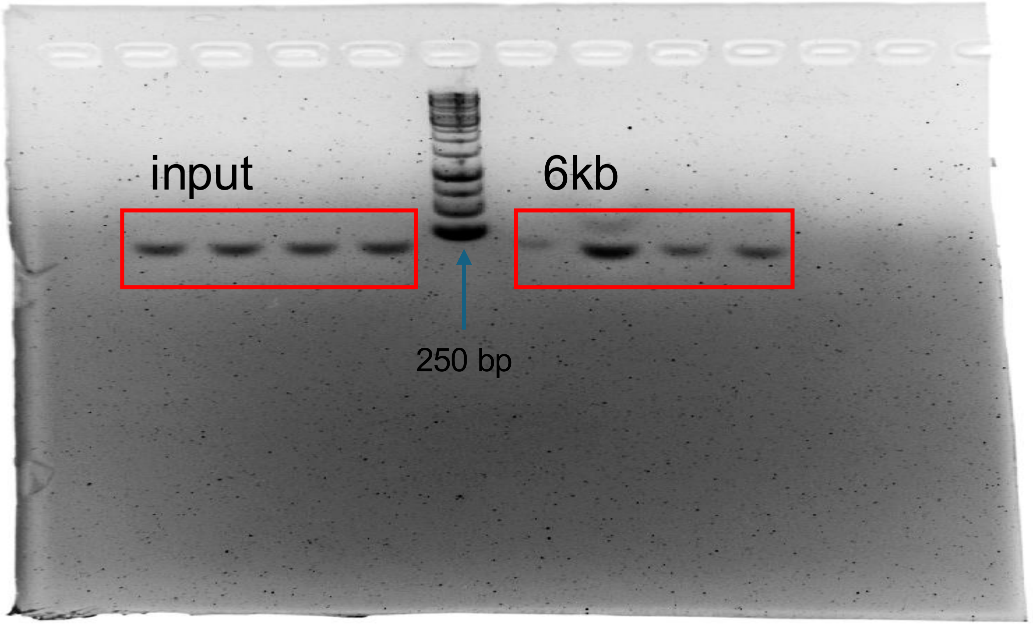

Supplement: gkag681_Supplemental_Files [file gkag681_supplemental_files.zip › source data.pdf]
