# Supplementary material for: CDW19S coordinates phasic end processing via distinct enzymatic activities
Source: Nucleic Acids Res. 2026 Jul 6;54(13):gkag681. doi: 10.1093/nar/gkag681 (PMC13335481; doi:10.1093/nar/gkag681)
Supplement: gkag681_Supplemental_Files [file gkag681_supplemental_files.zip › supple-clean-updated.pdf]

Supplementary Data

Figure S1. RIF1 and PTIP coordinate in phasic resection.

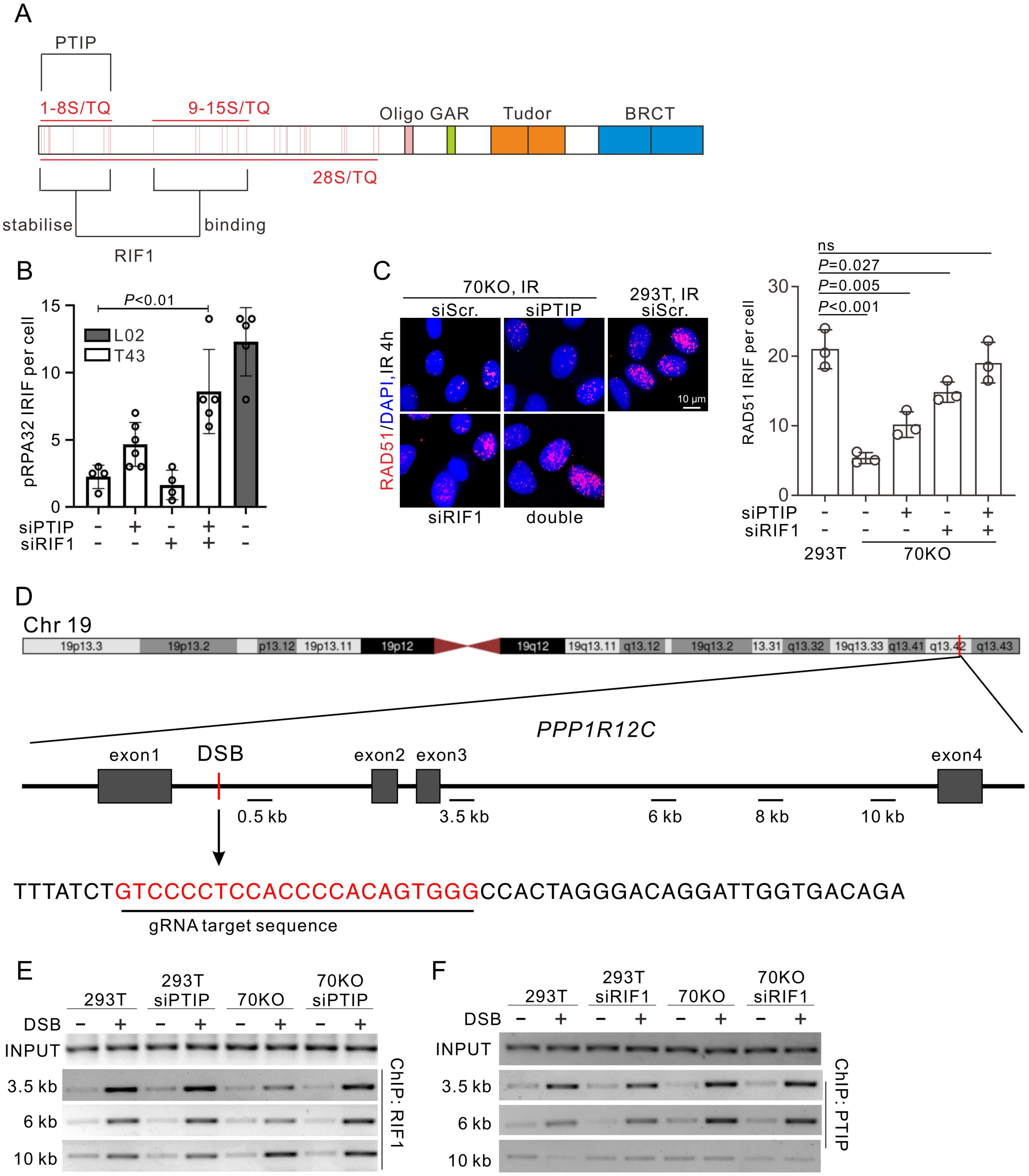

(**A**) Illustration of phosphor-binding of PTIP and RIF1 to the N-terminus of 53BP1. (**B**) pRPA32 IRIF assay in HBV+ T43 cells treated with individual or a combination of si*PTIP* and si*RIF1*. Parallel experiment carried out in parental line (L02, HBV free) was used as wild type control. (**C**) Immunofluorescence for RAD51 in *70KO* cells subjected to indicated siRNA treatments. (**D**)Schematic showing the site-specific DSB induced by CRISPR-gRNA at the human *PPP1R12C*

locus of chromosome 19. The gRNA targeting sequence in Intron 1 is highlighted in red. Upon transfection, CRISPR-gRNA complexes digest DNA within a range of 100 bp downstream of the target sequence. (**E-F**) ChIP analysis for endogenous RIF1 (E) and PTIP (F) in indicated cells. All histograms are presented as mean ± s.d. *P* values were calculated by a 2-way ANOVA test.

Figure S2. Effects of *RAP80* depletion on resection in CDW19S-deficient cells.

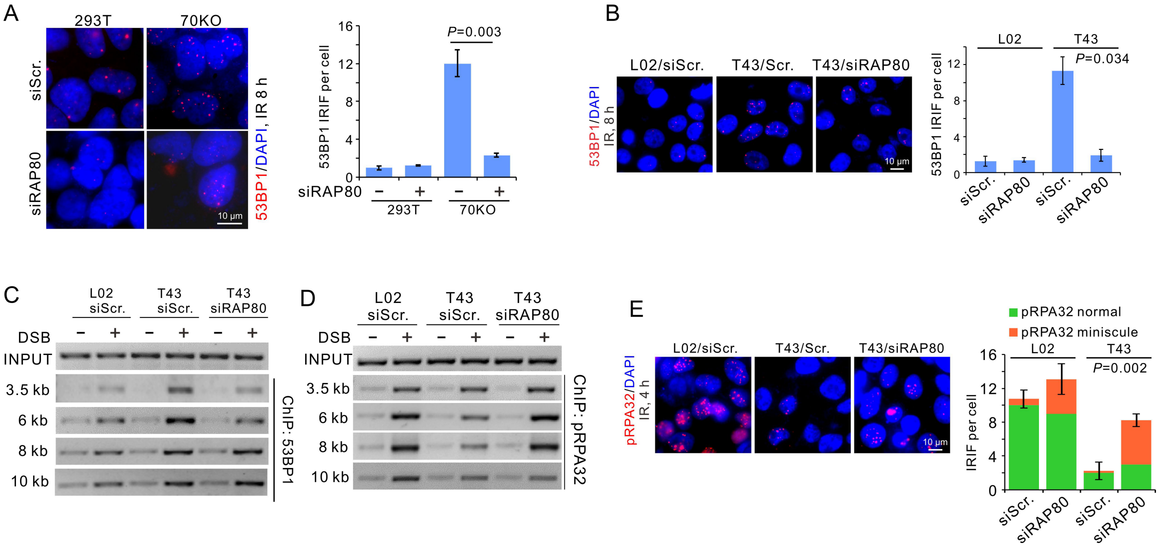

(**A-B**) Representative images and quantifications of 53BP1 IRIF in 293T/*70KO* (A) and L02/T43 (B) cells with si*RAP80* or not. (**C-D**) ChIP analysis for 53BP1 (C) and pRPA32 (D) in L02 and T43 cells upon si*RAP80* or not. (**E**) Representative images and quantifications of pRPA32 IRIF in L02 and T43 cells upon si*RAP80* or not, again notice the minuscule foci as with Fig. 2F. All histograms are presented as mean ± s.d. *P* values were calculated by a 2-way ANOVA test.

Figure S3. Identification and characterization of 6Kub in RAP80.

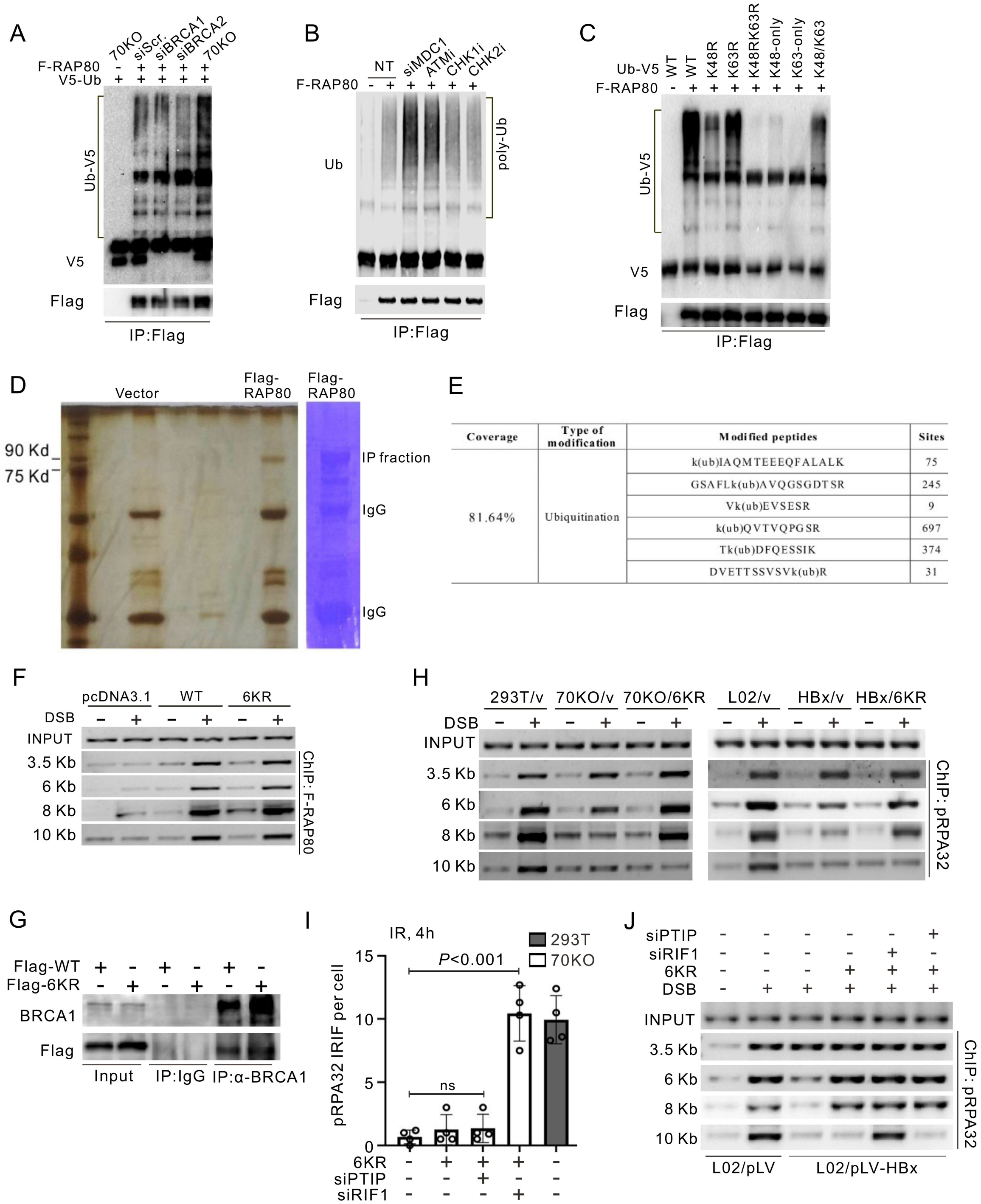

(**A**) Immunoblotting for RAP80 ubiquitin species after immunoprecipitation with Flag M2 beads in *70KO* or 293T cells transfected with si*BRCA1/2*. (**B**) RAP80 ubiquitination was detected in G2-synchronized 293T cells pre-treated with indicated siRNA or inhibitors including ATMi (KU-55933, 5 μM), CHK1i (LY2603618, 2.5 μM), or CHK2i (BML-277, 2.5 μM). (**C**) As in (A), ubiquitinated RAP80 was monitored in *70KO* cells co-expressing V5-tagged ubiquitin mutants. (**D**) Silver and

Coomassie blue staining for immunoprecipitated Flag-RAP80 from *70KO* cells. Bands of approximately 90 kD were excised and subjected to LC-MS/MS analysis. (**E**) Coverage of ubiquitinated peptides and lysines of RAP80 identified by MS/MS. (**F**) DSB-association of Flag-tagged RAP80 and 6KR mutant assayed by ChIP. (**G**) co-IP for wild-type RAP80-Flag or 6KR with BRCA1. BRCA1 was overexpressed from pLVX-IRIS-Green and pulled down, then detected with α-BRCA1 (Millipore). (**H**) ChIP assays for pRPA32 in *70KO* (left) and *HBx*-expressing L02 (right) cells upon $6KR^{EE}$. (**I**) Enumeration for pRPA32 IRIF in *70KO* or 293T cells treated with si*RIF1*, si*PTIP* and $6KR^{EE}$. (**J**) ChIP analysis for pRPA32 in L02 cells sunjected to si*RIF1*, si*PTIP,* and $6KR^{EE}$.

Figure S4. Reproduction of phasic resection at additional DSB sites.

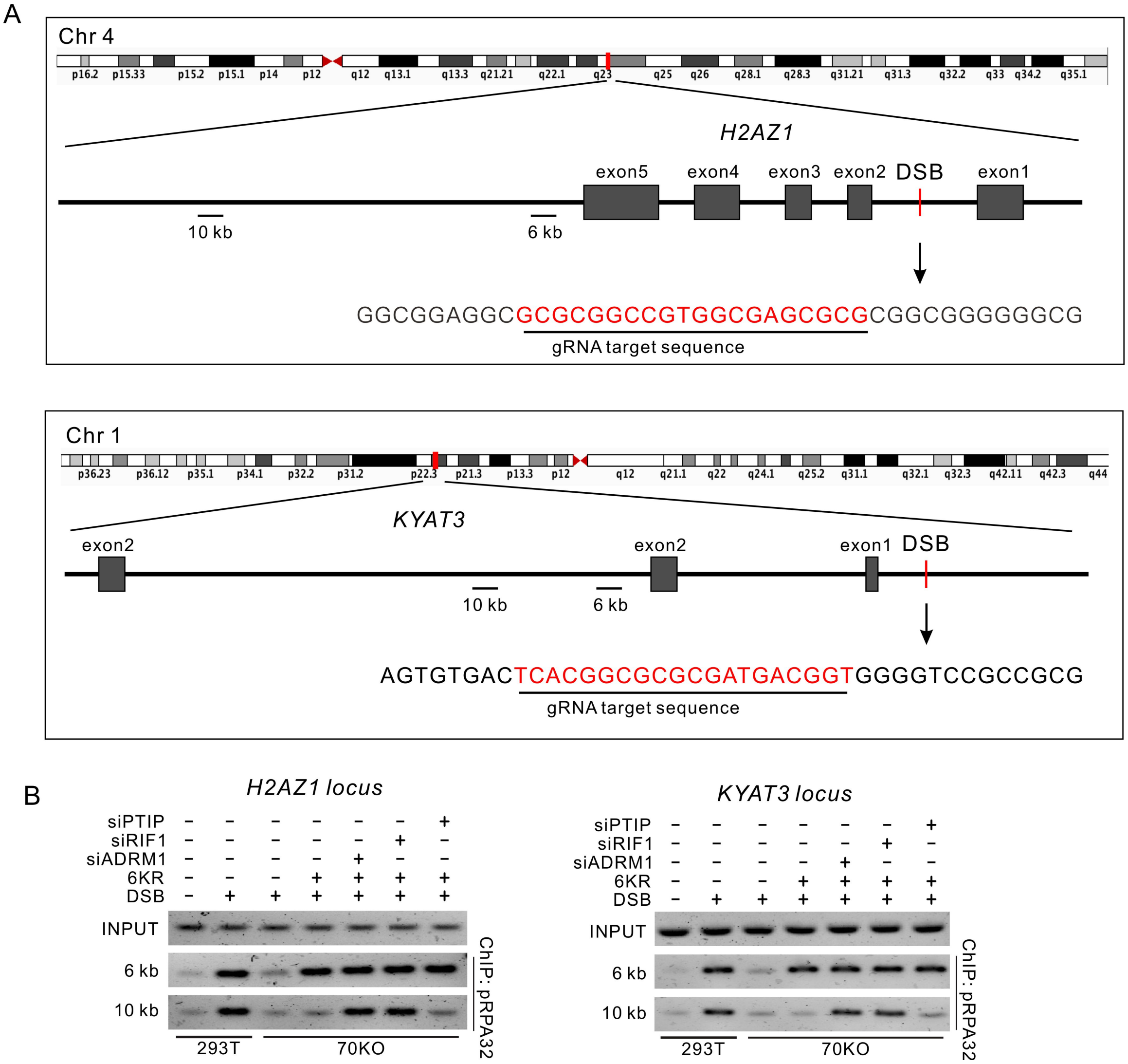

(**A**) Schematic showing the two additional DSB sites induced by CRISPR-gRNA: *H2AZ1* on chromosome 4 and *KYAT3* on chromosome 1, respectively. (**B**) ChIP assays for pRAP32 at DSBs of H2AZ1 (left) and KYAT3 (right) loci in 293T/70KO cells upon indicated treatments.

Figure S5. *6KR* causes hyper-resection in wild type cells.

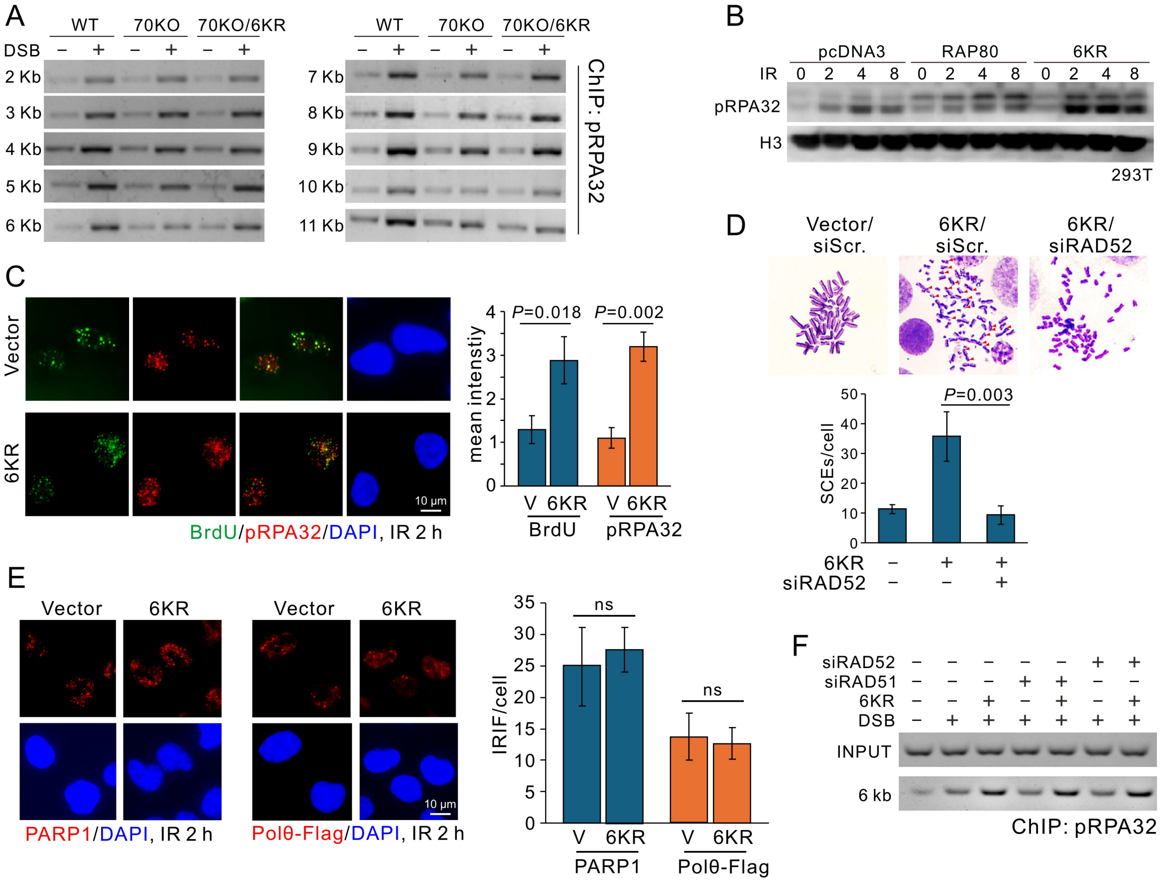

(**A**) ChIP analysis for pRPA32 in 293T/*70KO* cells upon *6KR* expression or not. (**B**) Immunoblotting for pRPA32 in 293T cells upon introducing pcDNA3 vector control or *RAP80* WT/*6KR* alleles. Cells were exposed to 10 Gy IR and harvested at the indicated time point. (**C**) Representative images and quantifications of pRPA32 and BrdU IRIF in L02 cells upon *6KR* expression or not. (**D**) Sister chromatid exchange (SCE) analysis in L02 cells with the indicated treatment. (**E**) As in (C), IRIF assays for PARP1 and Polθ were performed in L02 cells. For Polθ staining, cells were transfected with the pLVX-POLQ-Flag plasmid 48 hours before IR irradiation and then stained with an anti-Flag antibody. (**F**) ChIP assays for pRPA32 in L02 cells subjected to indicated treatments. All histograms are presented as mean ± s.d. P values were calculated by a 2-way *t*-test in (C) and (E), or 2-way ANOVA test in (D).

Figure S6. *POH1* deletion causes long-range resection defects.

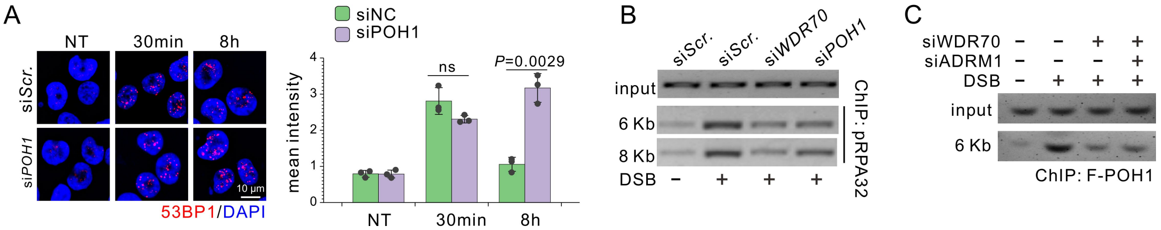

(**A**) 53BP1 IRIF assay in L02 cells upon si*POH1* or not. Histograms are presented as mean ± s.d. *P* values were calculated by a 2-tailed *t*-test. (**B**) ChIP analysis for pRPA32 showing si*POH1* in L02 cells displayed a long-range resection defect at 6 – 8 kb distal to DSB. (**C**) ChIP analysis for Flag-POH1 in L02 cells with individual or combined treatment of si*WDR70* and si*ADRM1*.

Table S1. Plasmids used in this study

Plasmids	Source
pcDNA3-HA-HBx	Previous study
pCMV-Flag-WDR70	Previous study
pLVX-puro-HA-HBx	Previous study
pLVX-shWDR70	Purchased from Genechem
pLVX-sh53BP1	Purchased from Genechem
pCMV-NHEJ	Gift from Prof. Jun Chen, Zhejiang University
pCMV-HR	Gift from Prof. Jun Chen, Zhejiang University
pCMV-SSA	Gift from Prof. Jun Chen, Zhejiang University
pMC1-1-p84-g1	gRNA vector for *PPP1R12C/p84* locus, Purchased from Viewsolid biotech
pMC1-1-p84-g0	gRNA vector (empty), Purchased from Viewsolid biotech
pLVX-G-Flag-RAP80	This study
pLVX-G-Flag-RAP80-6KR	This study
pcDNA3.1-RAP80	This study
pcDNA3.1-RAP80-6KR	This study
pcDNA3.1-HA-ubiquitin	Gift from Prof. Changan Jiang
pcDNA3.1-V5-ubiquitin	Gift from Prof. Changan Jiang
pcDNA3.1-V5-ubiquitin-K48	Gift from Prof. Changan Jiang
pcDNA3.1-V5-ubiquitin-K48K63	This study
pcDNA3.1-V5-ubiquitin-K63	Gift from Prof. Changan Jiang
pcDNA3.1-V5-ubiquitin-K48R	Gift from Prof. Changan Jiang
pcDNA3.1-V5-ubiquitin-K63R	Gift from Prof. Changan Jiang
pcDNA3.1-V5-ubiquitin-K48RK63R	Gift from Prof. Changan Jiang
pLVX-G-BRCA1	This study
pCDNA3.1-PTIP-3xFlag	Purchased from YouBio
pLVX-G-ADRM1-K99R	Previous study
pLVX-G-Flag-POH1	Previous study
EJ2-GFP-PURO	Purchased from NovoPro
pLVX-G-Flag-RAP80-siRNA^{R}	This study
pLVX-G-Flag-RAP80-6KR-siRNA^{R}	This study
pLVX-G-POLQ-Flag	This study

Table S2. siRNA used in this study

Gene	Sequence	Source
si*WDR70*	5’-CUGCCAGAAUGGAAGCAUA-3’	Ribobio, (Guangzhou, China)
si*DDB1*	5’-CCUGUUGAUUGCCAAAAAC-3’	Ribobio
si*53BP1*	5’-GGCCUUUGCCUCUCAACAA-3’	Ribobio
si*BRCA1*	5’- GGAACCUGUCUCCACAAAG -3’	Ribobio
si*UIMC1(RAP80)*	5’-GAACCAGUGUUACCUAGAC-3’	Ribobio
si*RIF1*	5’ -GACUCACAUUUCCAGUCAA-3’	Ribobio
si*PTIP*	5’-GCAGAUGUCUGAUAAGCAA -3’	Ribobio
si*BRCA2*	5’- GAAGAACAAUAUCCUACUA-3’	Ribobio
si*POH1*	5’-AUACCGUCAGAGUGAUUGA-3’	Ribobio
si*PSMD5*	5’-GGAUGACAGAAUCCUGGUU-3’	Ribobio
si*PSMD4*	5’-GCACCGACAAGGCAAGAAU-3’	Ribobio
si*PSMD7*	5’-UGGUCAUCAUUGAUGUGAA-3’	Ribobio
si*PSMC1*	5’-GGAGACCUAUGCAGAUAUU-3’	Ribobio
si*PSMD3*	5’-GCCGCAAAGUGUUACUAUU-3’	Ribobio

Table S3. Antibodies used in this study

Antibodies	Source
Rabbit anti-phosphor-Serine 33, RPA32	NOVUS, NB100-544
Mouse anti-α-Tubulin	Sigma, T6074
Rabbit anti-RAD51	Proteintech, 14961-1-AP
Rabbit anti-53BP1	Bethyl, A300-272A
Rabbit anti-phosphor-Serine 1524, BRCA1	Bethyl, A300-001A
HRP-conjugated anti-mouse IgG	DAKO, P0260
HRP-conjugated anti-rabbit IgG	DAKO, P0448
FITC- conjugated anti-mouse IgG	Sigma, F0257
CY3- conjugated anti- rabbit IgG	Sigma, C2306
Rabbit anti-UIMC1 (RAP80)	Protientech, 13642-1-AP
Rabbit anti-RIF1	NOVUS, NB100-1586
Rabbit anti-PTIP	NOVUS, NB100-577
Rabbit anti-BRCA1 (for IP and ChIP)	Millipore, 07-434
Mouse anti-γH2AX	Millipore, 05-636
Rat anti-HA	Roche, 11867423001
Rabbit anti-Flag	custom-made
Mouse anti-ubiquitin	PTMbio, PTM-5798
Rabbit anti-H3	ABclonal, AC070
Rabbit anti-RAD52	LSBio, LS-C176555
Mouse anti-V5	Thermo, R960-25
Rabbit anti-PARP1	HUABIO, ET1608-56
Rabbit anti-BrdU	Roche, clone BMG 6H8

Table S4. Primers used in this study

	Primers	Application
Primer 1	ATCATGGCCGACAAGCAGAAGAACG	efficiency of DSB repair, Normalize, forward
Primer 2	CGGCGGCGGTCACGAACTCC	efficiency of DSB repair, Normalize, reverse
Primer 3	TGACCACCCTGACCTACG	efficiency of DSB repair, HR and SSA repair, forward
Primer 4	CACCTTGATGCCGTTCTTCTGC	efficiency of DSB repair, repair, reverse
Primer 5	TCGGAGCAAGCTTGATTTAGGTGA	efficiency of DSB repair, NHEJ repair, forward
Primer 6	CTAACTTTGGCTCTTCACCT	Amplicon at 0.5 Kb, forward
Primer 7	GATGGAGAAAGAGAAAGGGA	Amplicon at 0.5 Kb, reverse
Primer 8	TCGCCAGTGCTTTTTCTTTT	Amplicon at 3.5 Kb, forward
Primer 9	GTTGGGGGATGATGAAAATG	Amplicon at 3.5 Kb, reverse
Primer 10	TCCTGCAGAAATTGCTCATAAC	Amplicon at 6 Kb, forward
Primer 11	ACGGCTGAGGGTCTTTCCAGT	Amplicon at 6 Kb, reverse
Primer 12	GACCAGCCTGGCCAACATG	Amplicon at 8 Kb, forward
Primer 13	CTGTTGCCCAGGCTGGAGTG	Amplicon at 8 Kb, forward
Primer 14	ATGGCTCATGCCTGTAATCC	Amplicon at 10 Kb/ Total DNA, forward

Primer 15	CAGCCTCCCAAGTAGCTGAG	Amplicon at 10 Kb/ Total DNA, reverse
Primer 16	GACGACGATAAGGAATTCATGCCACGGAGAAAGAAAAAAGTT	RAP80 to pLVX-G, forward
Primer 17	TAGTCTCGAGGAATTCTCAGAATTTTCTCCTTCTTCCTC	RAP80 to pLVX-G, reverse
Primer 18	GACGACGATAAGGAATTCATGCCACGGAGAAAGAAA AAAGTAGGGAAGTC	RAP80-K9R, forward
Primer 19	GATGCATTCATTGTGATATCCGATAGTGATGGAGAG	RAP80-K31R, forward
Primer 20	GGATATCACAATGAATGCATCCTCAAGTCTACGCTT CCTCCTCACACTGAC	RAP80-K31R, reverse
Primer 21	GCCAAAAGAAGAATCGCACAGATGACAGAAGAAGAACAG	RAP80-K75R, forward
Primer 22	CATCTGTGCGATTCTTCTTTTGGCCAAACACTTTGC	RAP80-K75R, reverse
Primer 23	TCTGCCTTTCTCAGAGCTGTCCAGGGTAGCGGGGAC	RAP80-K245R, forward
Primer 24	GACAGCTCTGAGAAAGGCAGAACCCCTCCCAGTAC	RAP80-K245R, reverse
Primer 25	GCATCTGACTGGCACTCAAAAACCAGAGATTTCCAGGAAAGC	RAP80-K374R, forward
Primer 26	TTTTGAGTGCCAGTCAGATGCCCTAGACTCCTGCCTCTC	RAP80-K374R, reverse
Primer 27	TCAGAAGCCACAGATTGCTTAGTGGACTTTAAAAGACAAGTTAC	RAP80-K697R, forward
Primer 28	TAAGCAATCTGTGGCTTCTGAAATGGAAACAAAAGA	RAP80-K697R, reverse
Primer 29	GACGACGATAAGGAATTCATGGATTTATCTGCTCTTCGCGTTG	BRCA1 to pLVX-G, forward
Primer 30	TAGTCTCGAGGAATTCTCAGTAGTGGCTGTGGGGGATCTGGGG	BRCA1 to pLVX-G, reverse
Primer 31	CTCGATTCTACGGGCGGATCCATGCAGATCTTCGTCAGAACG	ubiquitin-K48K63 to pcDNA3.1-V5, forward

Primer 32	CGTCGTATGGGTATCTAGA TCAACCACCTCTTAGTCTTAAGACAAGATGTAA GGTCGACTCTTTCTG	ubiquitin-K48K63 to pcDNA3.1-V5, reverse
Primer 33	AGGCTAGAGAGGTTCTCTACCCTGC	Amplicon at 6 Kb, H2AZ1 locus, forward
Primer 34	GTCATATTCAAGAAATCATTGCCAG	Amplicon at 6 Kb, H2AZ1 locus, reverse
Primer 35	GGTGGTGCATGCCTGTAGTCCCAGC	Amplicon at 10 Kb, H2AZ1 locus, forward
Primer 36	CTTCTAATTAGCATGCAGTGGGTC	Amplicon at 10 Kb, H2AZ1 locus, reverse
Primer 37	CAGGGAGAAGTGTTAGGAATGGAG	Amplicon at 6 Kb, KYAT3 locus, forward
Primer 38	CCTTACATTACTGATGAGTTTGTTCAAAC	Amplicon at 6 Kb, KYAT3 locus, reverse
Primer 39	TTGGTGTCCATTTGCATGAAATGG	Amplicon at 10 Kb, KYAT3 locus, forward
Primer 40	AAGTACACAGGCAACAAAGAGTACG	Amplicon at 10 Kb, KYAT3 locus, reverse
